# An Integrated Carbon Nitride‐Nickel Photocatalyst for the Amination of Aryl Halides Using Sodium Azide

**DOI:** 10.1002/anie.202203176

**Published:** 2022-04-12

**Authors:** Arjun Vijeta, Carla Casadevall, Erwin Reisner

**Affiliations:** ^1^ Yusuf Hamied Department of Chemistry University of Cambridge Cambridge CB2 1EW UK

**Keywords:** Carbon Nitride, Green Chemistry, Heterogeneous Catalysis, Organic Synthesis, Photocatalysis

## Abstract

The synthesis of primary anilines via sustainable methods remains a challenge in organic synthesis. We report a photocatalytic protocol for the selective synthesis of primary anilines via cross‐coupling of a wide range of aryl/heteroaryl halides with sodium azide using a photocatalyst powder consisting of nickel(II) deposited on mesoporous carbon nitride (Ni‐mpg‐CN_*x*_). This heterogeneous photocatalyst contains a high surface area with a visible light‐absorbing and adaptive “built‐in” solid‐state ligand for the integrated catalytic Ni site. The method displays a high functional group tolerance, requires mild reaction conditions, and benefits from easy recovery and reuse of the photocatalyst powder. Thereby, it overcomes the need of complex ligand scaffolds required in homogeneous catalysis, precious metals and elevated temperatures/pressures in existing protocols of primary anilines synthesis. The reported heterogeneous Ni‐mpg‐CN_*x*_ holds potential for applications in the academic and industrial synthesis of anilines and exploration of other photocatalytic transformations.

## Graphical abstract

A heterogeneous photocatalytic method is reported for the selective synthesis of primary anilines via cross‐coupling of a wide range of aryl/heteroaryl halides with sodium azide using nickel deposited on a mesoporous carbon nitride material. This procedure removes the need for sophisticated ligands, precious metals, and elevated temperatures/pressures, and it takes place under mild reaction conditions with a recyclable catalyst.

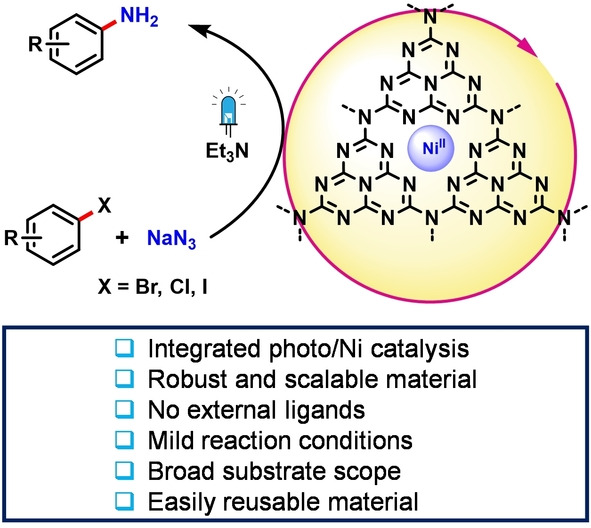

Anilines are ubiquitous motifs in pharmaceuticals, natural products, dyes, agrochemicals and functional materials,[^1^, ^2^] resulting in a persistent demand for simple, selective, and green methods for their preparation. Traditionally, primary anilines are synthesized by a “nitration‐hydrogenation pathway”, but this method produces large amounts of environmentally toxic waste and suffers from functional group incompatibilities due to its harsh experimental conditions (Scheme 1a).^[3]^ Alternatively, complex and sensitive primary anilines can be prepared by homogeneous transition metal catalysis such as palladium‐catalyzed (Hartwig‐Buchwald and Chan‐Lam) and copper catalyzed (Ullmann) cross‐coupling reactions of aryl halides with ammonia/ammonium salts (Scheme 1b).[^4^, ^5^, ^6^, ^7^, ^8^] These direct cross‐coupling approaches have already become important laboratory methods, but the requirement of air‐sensitive metal complexes and synthetically demanding ligand systems, strong base and elevated temperature (>80 °C) make them less desirable. In addition, the non‐recyclability and instability of these molecular catalysts limit the practical use at scale.

**Scheme 1:**
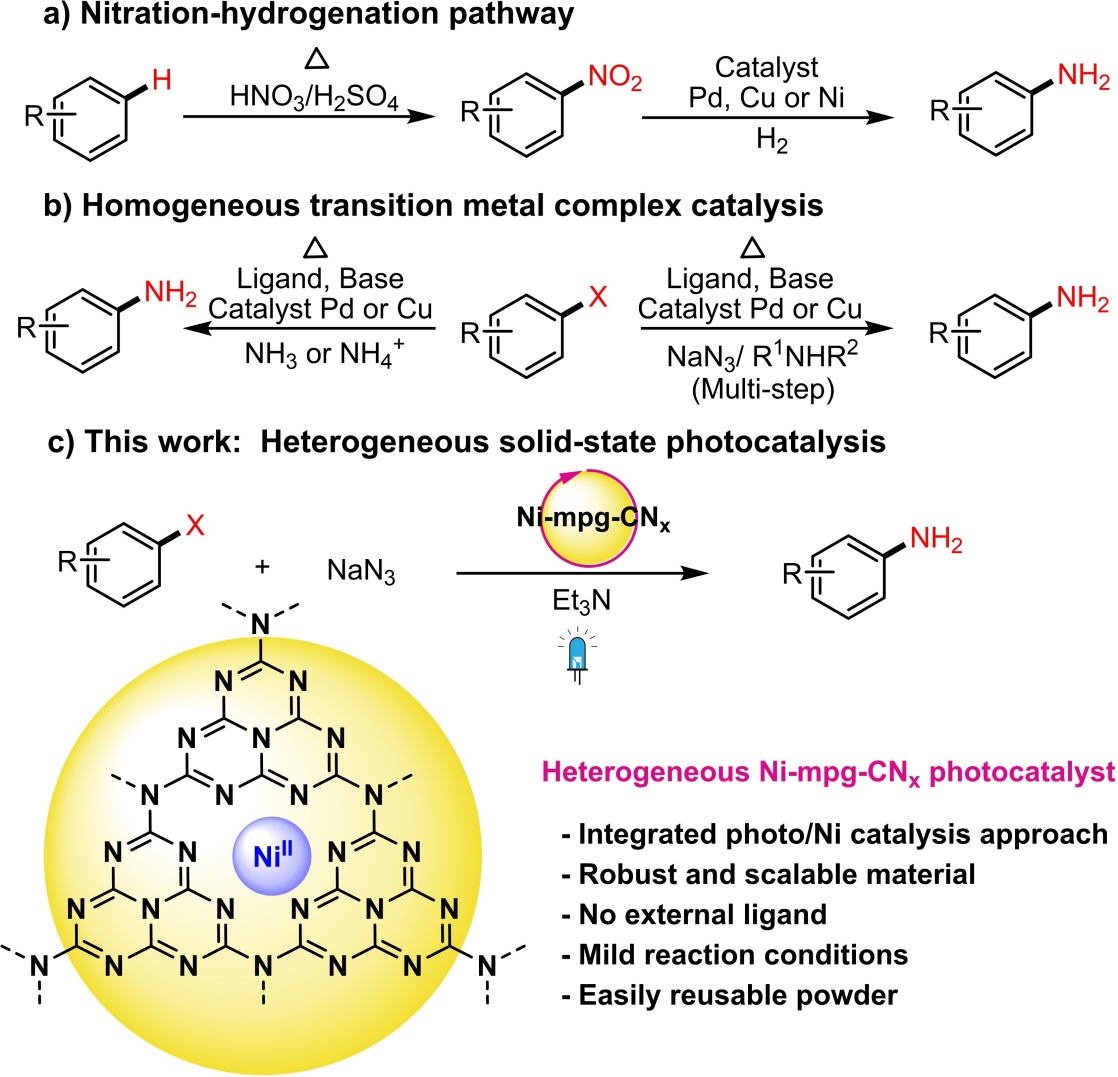
Methods for primary aniline synthesis.

The direct utilization of ammonia in these methods is another challenge and includes limitations from (*i*) catalyst poisoning by ammonia‐induced displacement of ancillary ligands from the metal complex, (*ii*) uncontrolled multiarylation arising from the competition of the reactive aniline product versus ammonia as a contending amine source and (*iii*) the requirement of significant ammonia pressure (3–4 bars).[^9^, ^10^] Other amine sources such as azides, silyl amines, imines and amides have been employed as alternatives, but the resulting coupling products often require thermal activation and/or an additional reaction step (cleavage or reduction) to release the free anilines while also producing unwanted side products.[^11^, ^12^, ^13^, ^14^, ^15^] Hence, there is considerable interest for simple, scalable and sustainable approaches for primary aniline synthesis.

Photocatalysis, especially in combination with transition metal catalysis (dual catalysis), is emerging as an energy‐efficient and sustainable strategy for chemical synthesis under mild conditions. It has been extensively studied for the synthesis of secondary or tertiary anilines,[^16^, ^17^, ^18^, ^19^] but is underexplored for the preparation of primary anilines. The rare reports include direct oxidative coupling of electron rich arenes with ammonia or ammonium salts by employing strongly oxidizing photocatalysts, such as acridinium salts or quinolinium ions based molecular catalysts and boron carbon nitride semiconductor.[^20^, ^21^, ^22^, ^23^] These methods feature mild reaction conditions with reduced waste generation, but their scope is limited to electron‐rich anilines affording only moderate yields with compromised regioselectivity. Recently, an alternative pathway presenting condensation of ammonia with cyclohexyl ketones, followed by dehydrogenation using an Ir‐photosensitizer and a Co‐catalyst to generate primary anilines has been reported, but the method relies on the availability of the cyclohexyl ketones.^[24]^ The development of a photocatalytic method to access electron‐deficient primary anilines still remains a challenge.

Herein, we report a simple and efficient photocatalytic method for the cross‐coupling of aryl halides with sodium azide to prepare primary anilines under mild conditions (Scheme 1c). The method employs Ni‐deposited mesoporous carbon nitride (Ni‐mpg‐CN_*x*_) as an integrated heterogeneous photocatalyst, displays broad substrate scope and represents the first fully heterogeneous photocatalytic system for the synthesis of primary anilines. Reactivity and kinetic studies as well as density functional theory (DFT) calculations are reported to provide mechanistic insights into the function of Ni‐mpg‐CN_*x*_.

Graphitic carbon nitride (CN_*x*_) is an emerging material in organic photocatalysis, which can be attributed to its inexpensive, robust and easy/scalable synthesis alongside the tunable optical properties.[^25^, ^26^, ^27^, ^28^] The heterogeneous nature of CN_*x*_ allow easy isolation and reuse, which is a significant advantage over homogeneous photocatalysts.[^29^, ^30^, ^31^, ^32^] To overcome associated challenges of the use of CN_*x*_ in dual catalysis with molecular co‐catalysts, e.g., with Ni polypyridyl complexes,[^33^, ^34^, ^35^, ^36^, ^37^] we and others have recently introduced Ni‐deposited carbon nitride as a fully heterogeneous photocatalytic system to perform C−O cross‐coupling reactions.[^38^, ^39^] Ni‐mpg‐CN_*x*_ has been synthesized and characterized according to a previously reported procedure and details are presented in the Supporting Information (see Figures S1–S4 for transmission electron microscopy (TEM), powder X‐ray diffraction, attenuated total reflectance infrared and UV/Vis spectroscopy).^[39]^ Inductively coupled plasma optical emission spectrometry (ICP‐OES) confirmed that 5.9 wt.% of Ni was deposited on the mpg‐CN_*x*_ (Table S1).

The amination was first performed with 4‐bromobenzonitrile as a model substrate and sodium azide as the amine source under visible light irradiation with the Ni‐mpg‐CN_*x*_ powder. Different solvents, bases, temperatures, concentrations of substrates and photocatalyst (Table S2–S6) were studied and the optimized conditions are given in Table 1 (entry 1). The desired aniline product **1** was obtained in 88 % yield upon irradiating (LED, *λ*=447±20 nm, 2.4 W at 700 mA) for 24 h,^[40]^ using a methanol:water (5 : 3) mixture containing triethyl amine as a base/electron donor under N_2_ at 60 °C. Only minor amounts of the dehalogenated product **1** **a**, the corresponding phenol **1** **b** and solvent coupled aryl ether product **1** **c** were observed as side products.

**Table 1:** Standard conditions and control experiments for photocatalytic amination.^[a]^

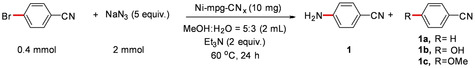						
						
Entry	Deviation	Conversion [%]	**1** [%]	**1a** [%]	**1b** [%]	**1c** [%]
1	none	>99	88 (84)^[b]^	5	5	traces
2	Ni‐CN_*x*_	20	16	traces	traces	n.d.^[c]^
3	Ni‐^NCN^CN_*x*_	27	5	21	traces	n.d.
4	mpg‐CN_*x*_+5 wt % NiCl_2_^[d]^	82	63	12	5	n.d.
5	mpg‐CN_*x*_+1 wt % NiCl_2_	80	60	12	5	traces
6	No Ni‐mpg‐CN_*x*_	n.d.	n.d.	n.d.	n.d.	n.d.
7	No mpg‐CN_*x*_	n.d.	n.d.	n.d.	n.d.	n.d.
8	No NiCl_2_	12	5	5	traces	traces
9	No light	n.d.	n.d.	n.d.	n.d.	n.d.
10	No Et_3_N	27	17	<5	6	n.d.
11	MeCN : H_2_O (5 : 3); no donor	2	traces	traces	n.d.	n.d.
12	MeCN : H_2_O (5 : 3)	53	41	8	<5	n.d.

[a] Conditions: 4‐bromobenzonitrile (0.4 mmol), Ni‐mpg‐CN_*x*_ (10 mg), NaN_3_ (2 mmol), Et_3_N (0.8 mmol) in 2 mL methanol:water (5 : 3) after 24 h irradiation (*λ*=447±20 nm) at 60 °C under N_2_. Yields determined by ^1^H NMR spectroscopy using 1,3,5‐trimethoxybenzene as internal standard, except for [b], where the value in bracket shows isolated yield. [c] n.d.=not detected. [d] NiCl_2_ wt.% refers to the weight percentage of mpg‐CN_*x*_.

Investigation of other Ni‐deposited CN_*x*_ materials with similar optical and electronic properties (see Supporting Information for details), but decreased surface area, showed a significantly lower yield of **1** under comparable conditions (Table 1, entries 2, 3).^[41]^ Moreover, higher selectivity for **1** **a** was observed with Ni‐doped cyanamide functionalized carbon nitride (Ni‐^NCN^CN_*x*_), indicating the specific structural environment alongside a high surface area is contributing to the better performance of Ni‐mpg‐CN_*x*_ to generate **1**.[^42^, ^43^] Performing the reaction with mpg‐CN_*x*_ and separate addition of NiCl_2_ for the in situ formation of Ni‐mpg‐CN_*x*_ resulted in good activity and only a 20 % decrease in conversion yield (Table 1, entries 4, 5).^[39]^ Exclusion control experiments confirmed the necessity of all components (Table 1, entries 6–10). The significant yield of **1** (17 %) in the absence of triethylamine can be explained by the strongly oxidizing nature of CN_*x*_, which is sufficient to oxidize the solvent methanol (potential of mpg‐CN_*x*_ valence band=+1.7 V vs. SHE; conduction band=−1.0 V, and methanol *E*_ox_=+1.54 V).[^27^, ^44^] This interpretation is supported by replacing methanol by acetonitrile (an inert solvent) in the absence/presence of triethylamine (entries 11, 12, Table 1).

After optimizing the conditions, amination scope with various aryl and heteroaryl bromides was examined (Table 2). The reaction was amenable to several *para*‐substituted electron‐withdrawing groups, including nitrile, ketone, ester, amide, sulfone and trifluoromethyl groups and resulted in good to excellent yields (65–86 %) of aniline products (**1**–**7**). These electron deficient primary anilines were not accessible with the previously reported photocatalytic methods.[^21^, ^24^] Moreover, thermal primary aniline synthesis using ammonia, which often requires strong inorganic bases, displays either no reactivity or compromised yield with base sensitive functional groups such as esters, amides and nitriles.[^45^, ^46^] The *ortho*‐ and *meta*‐nitrile substituted anilines **8**, **9** were also synthesizable with 74–76 % yield. Selective formation of monosubstituted anilines **10**, **11** was observed starting from dibromoarenes, as the electron‐rich nature of the products was unfavorable to further amination. In general, the synthesis of electron‐withdrawing substituted anilines was preferred, although amination of aryl bromides with electron‐neutral and electron‐donating groups (**12**–**15**) was also achieved with slower kinetics and incomplete conversion (even after 60 h). Furthermore, the reaction showed good to excellent reactivity towards bromo heteroarenes, including indanone, phthalide, quinoline, isoquinoline, benzothiazole and pyridine derivatives, resulting in anilines **16**–**23**. The reaction could also be used for the synthesis of valuable pharmaceutical compounds, including benzocaine **24** and sulfanilamide **25**.[^47^, ^48^]

**Table 2:**
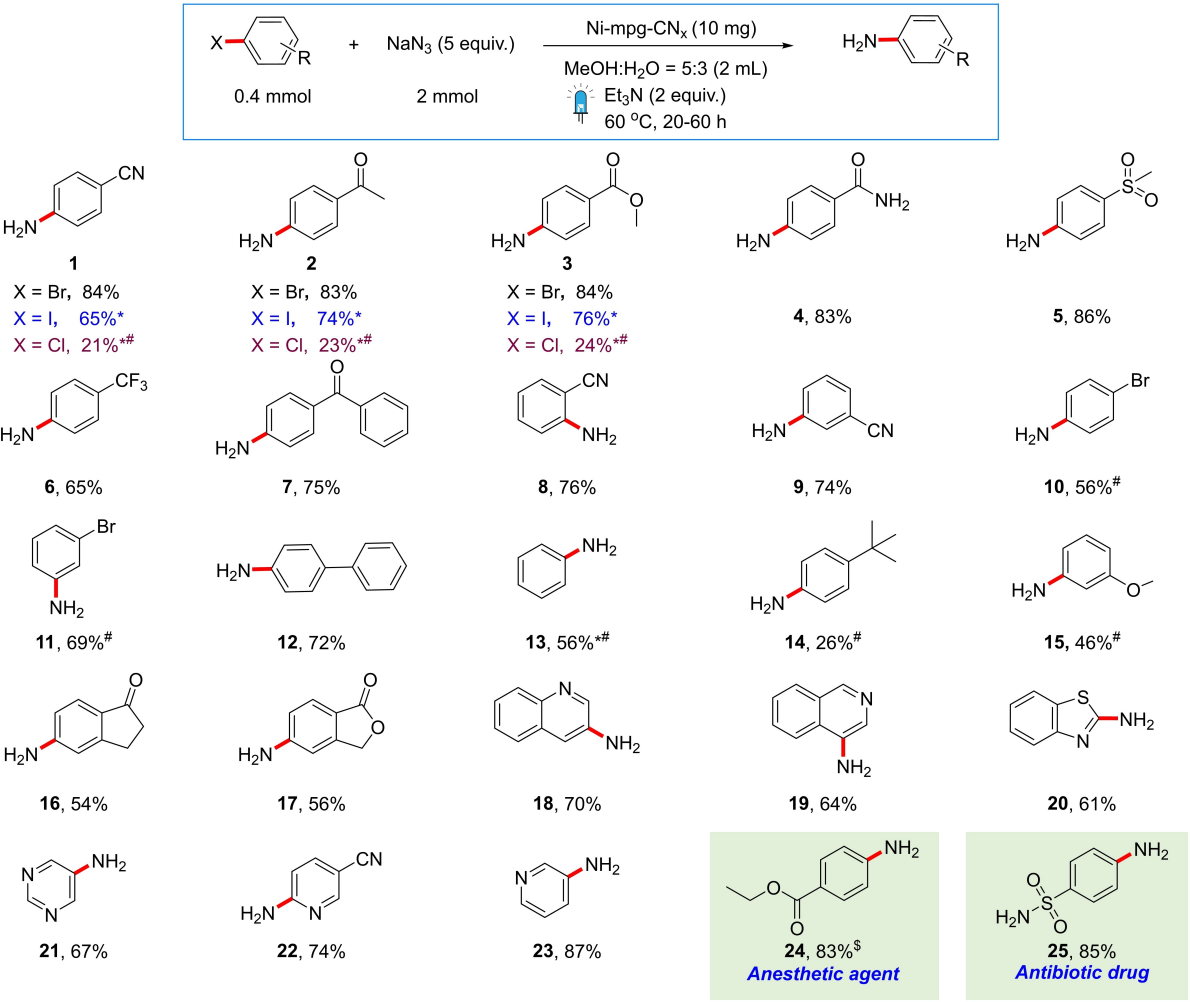
Substrate scope of photocatalytic amination.^[a]^

[a] Isolated yields given. * NMR yields, ^#^ reaction incomplete after 60 h and ^$^ ethanol instead of methanol used in solvent mixture.

Application to other aryl halides, including aryl iodides and chlorides, was also feasible for the coupling reaction. Although iodoarenes led to a significantly higher yield for the dehalogenated product (up to 20 % yield), anilines (**1**–**3**) could be obtained in moderate to good yields. Furthermore, the reaction kinetics was drastically affected by the stronger C−Cl bond, a common trend observed in Ni‐catalysis, resulting in less than 25 % yield of the product (**1**–**3**).^[35]^

The Ni‐mpg‐CN_*x*_ powder allowed simple reuse of the photocatalyst (Figure 1a, b; Supporting Information Figure S5). After completion of the cross‐coupling reaction between 4‐bromobenzonitrile and sodium azide (24 h), the photocatalyst powder was recovered by centrifugation. The recovered material was reused for several further photocatalytic cycles, each lasting 24 h, to generate coupling product **1** without any extra loading of catalytic components (Ni or mpg‐CN_*x*_). Although a gradual decrease in activity was observed, 75 % of aniline **1** was still obtained after the 4^th^ cycle. This result can be rationalized by the gradual leaching of Ni from the material (quantified by ICP‐OES) after each cycle (i.e., 4.2 % of total deposited Ni leached after the 1^st^ cycle and overall 17.6 % after the 4^th^ cycle, Table S1), which also resulted in a darker recovered material, potentially due to formation of small Ni aggregates as observed by high‐angle annular dark‐field scanning transmission electron microscopy (HAADF‐STEM) (Figure 1b).[^33^, ^38^] The leached Ni is suggested to be inactive according to photocatalytic reactivity studies performed in the presence of metallic Hg, additional NiCl_2_ and excess ethylenediaminetetraacetic acid (EDTA) (Figure 1c). No change in reaction rate was observed upon addition of Hg^0^ (>2000 equiv), which can poison catalytic Ni particles by the formation of an amalgam, suggesting that colloidal Ni, if formed, is not the active species.^[49]^ The oxidative addition of the substrate to Ni has been reported as the rate determining step,^[39]^ and the presence of more active Ni species should enhance the coupling rate. However, addition of external NiCl_2_ resulted in the same reaction rate as under standard conditions, suggesting that only the deposited Ni is active. Moreover, when the starting Ni‐mpg‐CN_*x*_ was mixed with a chelating agent such as EDTA all Ni (deposited and soluble) was sequestrated by the EDTA as confirmed by ICP‐OES (Table S1), preventing any catalytic reaction to proceed. The experiments support that the deposited cationic Ni sites on the mpg‐CN_*x*_ matrix are responsible for the catalytic reaction. The easy isolation and reuse of the material shows potential for sustainable and cost‐effective primary anilines preparation.

**Figure 1:**
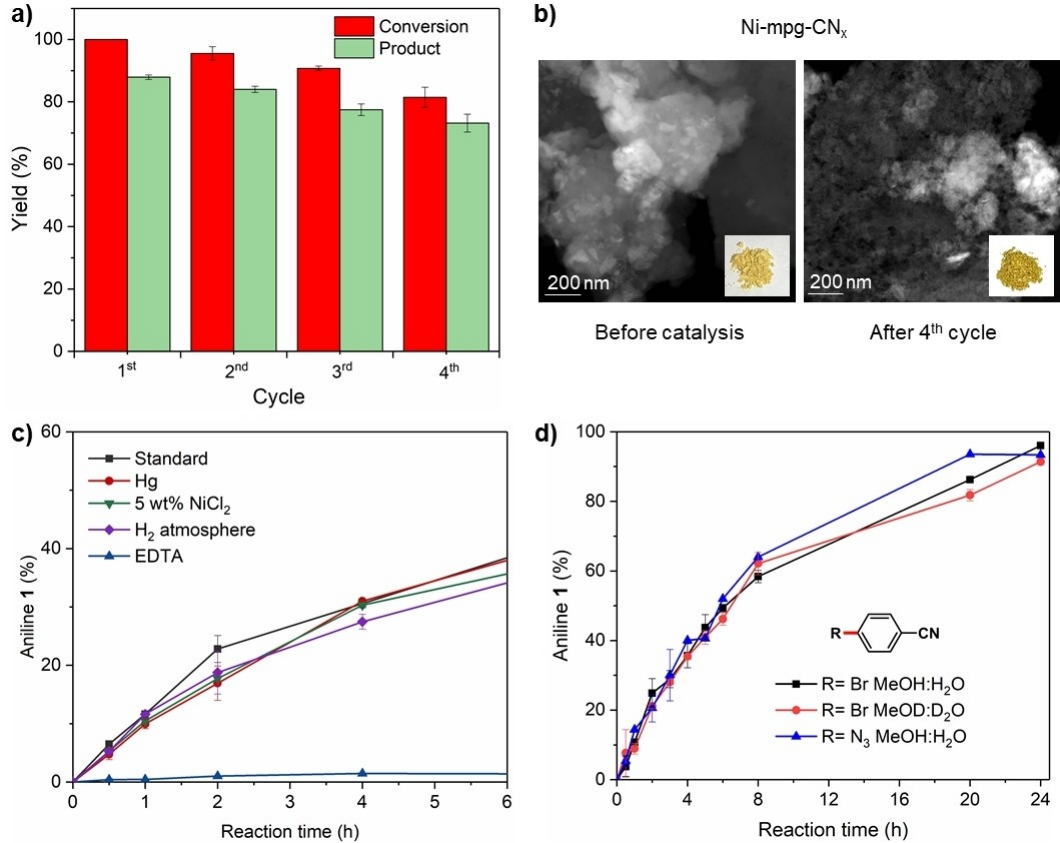
a) Recycling experiment performed under standard condition for 24 hours. b) HAADF‐STEM images and color (inset) of Ni‐mpg‐CN_*x*_ before catalysis and after the 4^th^ cycle. c) Reactivity studies under different conditions. d) Kinetics of photocatalytic amination in different conditions, NaN_3_ has not been used with 4‐azidobenzonitrile (blue trace). Yields determined by ^1^H NMR spectroscopy.

Next, to understand the mechanism (Scheme 2), reactivity, kinetic and DFT studies were performed. An aryl azide intermediate can be expected in the coupling reaction, but it was not observed by analyzing the reaction crude by ^1^H NMR spectroscopy (Figure S6). Thus, experiments were performed with 4‐azidobenzonitrile as the substrate under various conditions to investigate the potential role of azide as an amine source. A yield of 93 % was obtained for the reduced aniline **1** under standard conditions (without sodium azide), and only 4 % of **1** in the absence of light (Table S7). This indicates that photoredox‐mediated azide to amine reduction is efficient, which is consistent with a previous report using a Ru based photocatalyst.[^50^, ^51^] Kinetic studies starting with either 4‐bromobenzonitrile and 4‐azidobenzonitrile revealed the same rates of primary aryl amine **1** formation (Figure 1), which supports that a 4‐azidobenzonitrile intermediate would not accumulate in the coupling reaction.

**Scheme 2:**
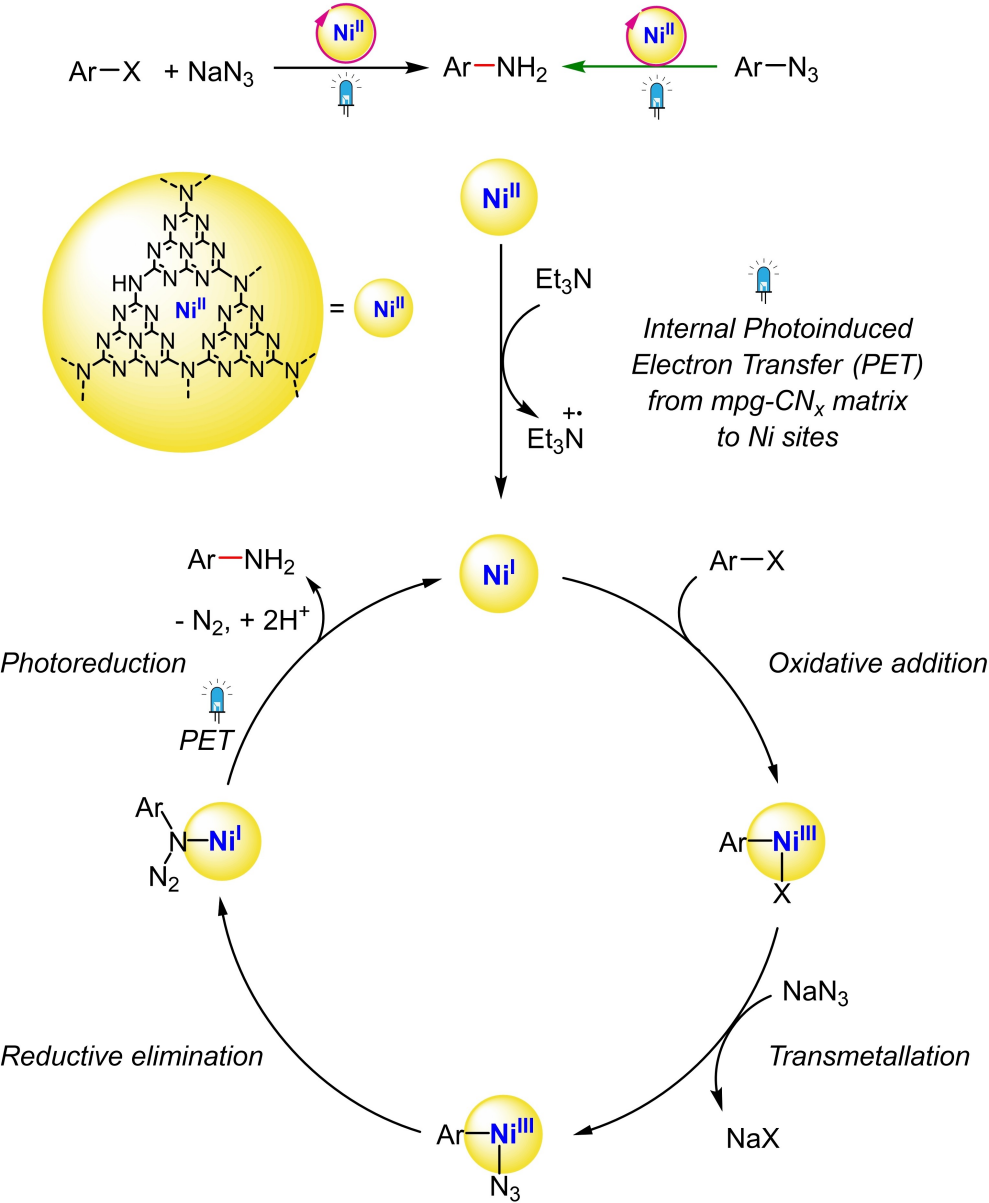
Proposed mechanism of photocatalytic amination reaction.

On the basis of mechanistic studies, which are supported by DFT calculations at the B3LYP/6‐31G*//B3LYP/cc‐pVTZ level of theory (see Supporting Information), and previous reports, we tentatively propose the catalytic cycle as shown in Scheme 2.[^39^, ^51^, ^52^, ^53^] Visible light irradiation of Ni‐mpg‐CN_*x*_ generates a photoexcited electron‐hole pair. The photogenerated holes in the valence band are quenched by triethylamine. Simultaneously, the photoexcited electrons are transferred from mpg‐CN_*x*_ matrix to the deposited Ni^II^ center to generate a Ni^I^ species. The formation of Ni^0^ upon consecutive reduction by mpg‐CN_*x*_ is thermodynamically and kinetically less favorable (see DFT calculations in Supporting Information), although the involvement of a Ni^0/II^ cycle has also been discussed previously.[^35^, ^54^] Then, an oxidative addition of the aryl halide is thermodynamically feasible (Δ*G* by DFT=1.7 kcal mol^−1^) generating a Ni^III^ species. This is followed by transmetallation generating a Ni^III^‐azide intermediate. The intermediate undergoes reductive elimination to generate Ni^I^ species coordinated with the aryl azide. Finally, a selective photoreduction occurs generating the aniline product and thus, the cycle completes. A speculative mechanism based on DFT calculations for the photoreduction step to generate a selective aniline is presented in the Supporting Information.

In conclusion, we report Ni‐mpg‐CN_*x*_ as an integrated heterogeneous photocatalyst for visible‐light driven synthesis of primary anilines by cross‐coupling of aryl halides with NaN_3_ as the amine source in the presence of an electron donor. The presented system demonstrates a broad substrate scope including aryl and heteroaryl bromides with substantial functional group tolerance. Additionally, the heterogeneous Ni‐mpg‐CN_*x*_ material is easily recovered from the reaction mixture and has been reused several times with only some loss in the activity. Kinetic studies and DFT calculations suggest Ni‐mpg‐CN_*x*_ also facilitates the reduction of aryl azide to selectively yield the primary anilines. We anticipate that this fully heterogeneous aniline synthesis protocol has potential for use in academic research and industry and that the Ni‐mpg‐CN_*x*_ photocatalyst holds promise in organic photocatalytic reactions.

## Conflict of interest

The authors declare no conflict of interest.

## Supporting information

As a service to our authors and readers, this journal provides supporting information supplied by the authors. Such materials are peer reviewed and may be re‐organized for online delivery, but are not copy‐edited or typeset. Technical support issues arising from supporting information (other than missing files) should be addressed to the authors.

Supporting Information

## Data Availability

Raw data related to this article are available at the University of Cambridge data repository: https://doi.org/10.17863/CAM.82740

## References

[1] P. Ruiz-Castillo , S. L. Buchwald , Chem. Rev. 2016, 116, 12564–12649. 27689804 10.1021/acs.chemrev.6b00512 PMC5070552

[2] T. Kahl , K.-W. Schröder , F. R. Lawrence , W. J. Marshall , H. Höke , R. Jäckh , Ullmann's Encyclopedia of Industrial Chemistry, Wiley-VCH, Weinheim, 2011, pp. 465–478.

[3] R. S. Downing , P. J. Kunkeler , H. van Bekkum , Catal. Today 1997, 37, 121–136.

[4] R. Dorel , C. P. Grugel , A. M. Haydl , Angew. Chem. Int. Ed. 2019, 58, 17118–17129; 10.1002/anie.201904795 31166642

[5] C. Sambiagio , S. P. Marsden , A. J. Blacker , P. C. McGowan , Chem. Soc. Rev. 2014, 43, 3525–3550. 24585151 10.1039/c3cs60289c

[6] J. Chen , J. Li , Z. Dong , Adv. Synth. Catal. 2020, 362, 3311–3331.

[7] I. P. Beletskaya , A. V. Cheprakov , Coord. Chem. Rev. 2004, 248, 2337–2364.

[8] X. Ribas , I. Güell , Pure Appl. Chem. 2014, 86, 345–360.

[9] G. D. Vo , J. F. Hartwig , J. Am. Chem. Soc. 2009, 131, 11049–11061. 19591470 10.1021/ja903049z PMC2823124

[10] A. Borzenko , N. L. Rotta-Loria , P. M. Macqueen , C. M. Lavoie , R. McDonald , M. Stradiotto , Angew. Chem. Int. Ed. 2015, 54, 3773–3777; 10.1002/anie.201410875 25573662

[11] Á. Georgiádes , S. B. Ötvös , F. Fülöp , Adv. Synth. Catal. 2018, 360, 1841–1849.

[12] S. Messaoudi , J. D. Brion , M. Alami , Adv. Synth. Catal. 2010, 352, 1677–1687.

[13] T. Ikawa , T. E. Barder , M. R. Biscoe , S. L. Buchwald , J. Am. Chem. Soc. 2007, 129, 13001–13007. 17918833 10.1021/ja0717414

[14] J. Barluenga , F. Aznar , C. Valdés , Angew. Chem. Int. Ed. 2004, 43, 343–345; 10.1002/anie.200352808 14705093

[15] K. G. Thakur , K. S. Srinivas , K. Chiranjeevi , G. Sekar , Green Chem. 2011, 13, 2326–2329.

[16] E. B. Corcoran , M. T. Pirnot , S. Lin , S. D. Dreher , D. A. Dirocco , I. W. Davies , S. L. Buchwald , D. W. C. Macmillan , Science 2016, 353, 279–283. 27338703 10.1126/science.aag0209 PMC5027643

[17] S. Singh , V. J. Roy , N. Dagar , P. P. Sen , S. R. Roy , Adv. Synth. Catal. 2021, 363, 937–979.

[18] I. Ghosh , L. Marzo , A. Das , R. Shaikh , B. König , Acc. Chem. Res. 2016, 49, 1566–1577. 27482835 10.1021/acs.accounts.6b00229

[19] A. Ruffoni , F. Juliá , T. D. Svejstrup , A. J. McMillan , J. J. Douglas , D. Leonori , Nat. Chem. 2019, 11, 426–433. 31011173 10.1038/s41557-019-0254-5

[20] N. A. Romero , K. A. Margrey , N. E. Tay , D. A. Nicewicz , Science 2015, 349, 1326–1330. 26383949 10.1126/science.aac9895

[21] D. A. Nicewicz , V. A. Pistritto , M. E. Schutzbach-Horton , J. Am. Chem. Soc. 2020, 142, 17187–17194. 32986412 10.1021/jacs.0c09296 PMC7720250

[22] Y. W. Zheng , B. Chen , P. Ye , K. Feng , W. Wang , Q. Y. Meng , L. Z. Wu , C. H. Tung , J. Am. Chem. Soc. 2016, 138, 10080–10083. 27467115 10.1021/jacs.6b05498

[23] M. Zheng , I. Ghosh , B. König , X. Wang , ChemCatChem 2019, 11, 703–706.

[24] S. U. Dighe , F. Juliá , A. Luridiana , J. J. Douglas , D. Leonori , Nature 2020, 584, 75–81. 32760044 10.1038/s41586-020-2539-7

[25] Y. Markushyna , C. A. Smith , A. Savateev , Eur. J. Org. Chem. 2020, 1294–1309.

[26] A. Savateev , I. Ghosh , B. König , M. Antonietti , Angew. Chem. Int. Ed. 2018, 57, 15936–15947; 10.1002/anie.201802472 30066478

[27] A. Vijeta , E. Reisner , Chem. Commun. 2019, 55, 14007–14010. 10.1039/c9cc07348e 31690891

[28] J. Shi , T. Yuan , M. Zheng , X. Wang , ACS Catal. 2021, 11, 3040–3047.

[29] J. Khamrai , I. Ghosh , A. Savateev , M. Antonietti , B. König , ACS Catal. 2020, 10, 3526–3532.

[30] S. Das , K. Murugesan , G. J. Villegas Rodríguez , J. Kaur , J. P. Barham , A. Savateev , M. Antonietti , B. König , ACS Catal. 2021, 11, 1593–1603.

[31] C. Cavedon , A. Madani , P. H. Seeberger , B. Pieber , Org. Lett. 2019, 21, 5331–5334. 31247752 10.1021/acs.orglett.9b01957 PMC6750873

[32] C. Wang , Q. Wan , J. Cheng , S. Lin , A. Savateev , M. Antonietti , X. Wang , J. Catal. 2021, 393, 116–125.

[33] S. Gisbertz , S. Reischauer , B. Pieber , Nat. Catal. 2020, 3, 611–620.

[34] I. Ghosh , J. Khamrai , A. Savateev , N. Shlapakov , M. Antonietti , B. König , Science 2019, 365, 360–366. 31346061 10.1126/science.aaw3254

[35] J. A. Malik , A. Madani , B. Pieber , P. H. Seeberger , J. Am. Chem. Soc. 2020, 142, 11042–11049. 32469219 10.1021/jacs.0c02848 PMC7467672

[36] Y. Qin , B. C. M. Martindale , R. Sun , A. J. Rieth , D. G. Nocera , Chem. Sci. 2020, 11, 7456–7461. 34123028 10.1039/d0sc02131h PMC8159281

[37] B. Pieber , J. A. Malik , C. Cavedon , S. Gisbertz , A. Savateev , D. Cruz , T. Heil , G. Zhang , P. H. Seeberger , Angew. Chem. Int. Ed. 2019, 58, 9575–9580; 10.1002/anie.201902785 31050132

[38] X. Zhao , C. Deng , D. Meng , H. Ji , C. Chen , W. Song , J. Zhao , ACS Catal. 2020, 10, 15178–15185.

[39] A. Vijeta , C. Casadevall , S. Roy , E. Reisner , Angew. Chem. Int. Ed. 2021, 60, 8494–8499; 10.1002/anie.202016511 PMC8048670 33559927

[40] A. Call , C. Casadevall , F. Acuña-Parés , A. Casitas , J. Lloret-Fillol , Chem. Sci. 2017, 8, 4739–4749. 30155221 10.1039/c7sc01276d PMC6100254

[41] H. Kasap , C. A. Caputo , B. C. M. Martindale , R. Godin , V. W. Lau , B. V. Lotsch , J. R. Durrant , E. Reisner , J. Am. Chem. Soc. 2016, 138, 9183–9192. 27337491 10.1021/jacs.6b04325 PMC4965840

[42] G. Filippini , F. Longobardo , L. Forster , A. Criado , G. Di Carmine , L. Nasi , C. D'Agostino , M. Melchionna , P. Fornasiero , M. Prato , Sci. Adv. 2020, 6, eabc9923. 33177092 10.1126/sciadv.abc9923 PMC7673726

[43] M. Melchionna , P. Fornasiero , ACS Catal. 2020, 10, 5493–5501.

[44] S. Roy , E. Reisner , Angew. Chem. Int. Ed. 2019, 58, 12180–12184; 10.1002/anie.201907082 PMC6771752 31273886

[45] R. A. Green , J. F. Hartwig , Angew. Chem. Int. Ed. 2015, 54, 3768–3772; 10.1002/anie.201500404 PMC4480680 25711163

[46] I. P. Beletskaya , A. V. Cheprakov , Organometallics 2012, 31, 7753–7808.

[47] J. M. Nusstein , M. Beck , Anesth. Prog. 2003, 50, 159–163. 14959903 PMC2007446

[48] K. A. Ford , Mol. Pharm. 2013, 10, 1171–1182. 23323940 10.1021/mp3004385

[49] V. Artero , M. Fontecave , Chem. Soc. Rev. 2013, 42, 2338–2356. 23165230 10.1039/c2cs35334b

[50] Y. Chen , A. S. Kamlet , J. B. Steinman , D. R. Liu , Nat. Chem. 2011, 3, 146–153. 21258388 10.1038/nchem.932 PMC3078041

[51] M. O. Konev , T. A. McTeague , J. W. Johannes , ACS Catal. 2018, 8, 9120–9124.

[52] N. A. Till , L. Tian , Z. Dong , G. D. Scholes , D. W. C. MacMillan , J. Am. Chem. Soc. 2020, 142, 15830–15841. 32786779 10.1021/jacs.0c05901

[53] J. Aragón , S. Sun , D. Pascual , S. Jaworski , J. Lloret-Fillol , Angew. Chem. Int. Ed. 2022, 61, e202114365; 10.1002/anie.202114365 35289039

[54] R. Sun , Y. Qin , S. Ruccolo , C. Schnedermann , C. Costentin , D. G. Nocera , J. Am. Chem. Soc. 2019, 141, 89–93. 30563318 10.1021/jacs.8b11262

