# Supplementary material for: An Integrated Carbon Nitride‐Nickel Photocatalyst for the Amination of Aryl Halides Using Sodium Azide
Source: Angew Chem Int Ed Engl. 2022 Apr 12;61(24):e202203176. doi: 10.1002/anie.202203176 (PMC9321912; doi:10.1002/anie.202203176)
Supplement: Supplementary file 1 — Supporting Information [file ANIE-61-0-s001.pdf]

Angewandte Chemie
Eine Zeitschrift der Gesellschaft Deutscher Chemiker

Supporting Information

# An Integrated Carbon Nitride-Nickel Photocatalyst for the Amination of Aryl Halides Using Sodium Azide

*A. Vijeta, C. Casadevall, E. Reisner**

# Supporting information

## Table of Contents

1. GENERAL INFORMATION ........ **S2**
2. COMPUTATIONAL STUDIES ........ **S4**
3. PRODUCTS CHARACTERIZATION ........ **S5**
4. SUPPORTING TABLES ........ **S11**
5. SUPPORTING FIGURES ........ **S16**
6. NMR SPECTRA OF PURIFIED PRODUCTS ........ **S23**
7. CARTESIAN COORDINATES. ........ **S48**
8. SUPPORTING REFERENCES. ........ **S52**

# 1. General information

Reagents used in this study were of the highest available purity acquired from commercial suppliers and used directly without any further purification, unless mentioned otherwise. Mesoporous, pristine and cyanamide functionalized carbon nitride were synthesized according to previously reported procedures.[1,2] Products were purified by flash column chromatography on silica gel 60 (0.040-0.063 mm mesh) from Material Harvest. Thin layer chromatography (TLC) was carried out on aluminum Merck Kieselgel 60 F254 sheets, visualized by ultraviolet irradiation (254 and 365). $^{1}H$ and $^{13}C$ NMR spectroscopy were recorded on a Bruker DPX 400 spectrometer at room temperature. Chemical shifts (δ) of $^{1}H$ and $^{13}C$ NMR spectra are given in ppm and the peaks were internally referenced against the residual solvent peak ($CDCl_3$ referenced at δ 7.26 ppm for $^{1}H$ and 77.16 ppm for $^{13}C$). NMR data were reported in the following form: Chemical shift, multiplicity, coupling constant and integration. Inductively coupled plasma-optical emission spectroscopy (ICP-OES) was carried-out in the Yusuf Hamied Department of Chemistry, University of Cambridge with a Perkin-Elmer ICP-OES chemical analyzer. Attenuated total reflection Fourier transform infrared (ATR-IR) spectra were recorded on a Nicolet iS50 spectrometer and reported in terms of absorption frequency ($cm^{-1}$). UV-vis diffuse reflection spectroscopy (DRS) of powder materials was carried out on an Agilent Cary 60 machine. Mass spectra were recorded on a Waters LCT premier Time of Flight mass spectrometer or Micromass Quadrupole-Time of Flight mass spectrometer. Reported mass values are within the error limits of 5 ppm.

### ICP-OES sample preparation

The nickel loading in the materials was determined by ICP-OES after digestion of the material (<1 mg) in concentrated $HNO_3$ (70%) (~1 mL) overnight and dilution to 10 mL with Milli-Q® water. The ICP-OES data are shown in Table S1.

### Preparation of mesoporous carbon nitride (mpg-$CN_x$)[1]

Cyanamide (3 g) was heated at 50 ºC until it completely melted and then a 40 wt.% dispersion of $SiO_2$ in water (7.5 g, Ludox HS) was added to form a homogeneous solution. The resultant transparent mixture was heated at 100 ºC to form a white solid. The white solid was ground and transferred to an alumina crucible and heated at a ramping rate of 2.3 °C $min^{-1}$ to reach a temperature of 550 ºC, and then kept at this temperature for a further 4 h under normal atmosphere (air). The resulting brown-yellow powder was treated with 4 M $NH_4HF_2$ for 24-48 h to remove the silica template. The powders were then washed three times with hot distilled water, ethanol, and acetone over suction filtration.

### Preparation of pristine carbon nitride ($CN_x$)[3]

In a 20 mL alumina crucible, 5 g of melamine was taken and heated at 550 ºC for 3 h at the ramping rate of 10 ºC per min under atmospheric condition. Pristine carbon nitride was obtained as yellow powder (3 g), which was grinded for 5-10 min using mortar and pestle till fine powder.

### Preparation of cyanamide functionalized carbon nitride ($^{NCN}CN_x$)[4]

A mixture of pristine $CN_x$ and KSCN (weight ratio 1:2) was prepared and heating first to 400 ºC for 1 hour followed by 500 ºC for 30 min with the ramping rate 30 ºC $min^{-1}$ under Ar atmosphere. After cooling naturally, the powder was washed with $H_2O$ and dried under vacuum at 60 ºC.

**Preparation of Ni deposited mpg-$CN_x$ (Ni-mpg-$CN_x$)**[5]

In a 20 mL microwave vial charged with a magnetic bead, mpg-$CN_x$ (0.3 g) was suspended in dry acetonitrile (12 mL), purged with $N_2$ and ultrasonicated for 10 min. Anhydrous $NiCl_2$ (50 mg) and anhydrous triethylamine (0.15 mL) were added to the suspension and the mixture was purged again for 5 min under $N_2$. The suspension was stirred at room temperature for 30 min, followed by heating under microwave at 80 $^oC$ for 2 h. After cooling to room temperature, the resulting yellow solid was collected by filtration sequentially washed with acetonitrile, hot water, boiling ethanol, boiling methanol, boiling acetone, and dried under vacuum. The material was characterized with powder X-ray, attenuated transmission reflectance Infra-ray (ATR-IR) and UV-Vis diffuse reflectance (UV DRS), which matches with the reported literature (Figure S2-S4).[5]

**General procedure for photocatalytic C-N coupling reactions**

All photocatalytic experiments were performed using blue LED photoreactors ($\lambda$ = 447 ± 20 nm, 1.03 W @ 700mA per LED) as a light source.[6] In a 10 mL borosilicate photoreactor vial charged with four glass beads (6 mm), Ni-mpg-$CN_x$ (10 mg), aryl halide (0.4 mmol) and sodium azide (2 mmol, 5 equiv.) were added. The vial was crimped by a septum-aluminum cap and purged with $N_2$ atmosphere via “vacuum and $N_2$ refill” cycles (x3). Then, 2 mL of a degassed methanol:water mixture (5:3) was added as a solvent and purged with $N_2$ atmosphere, followed by addition of triethylamine (0.8 mmol, 2 equiv.) using a syringe. The reaction mixture was then shaken rapidly using an orbital stirrer and irradiated using blue LEDs at 60 $^oC$ (Figure S7). The reaction was monitored by TLC. Upon completion of the irradiation, the reaction mixture was purified by flash column chromatography. In case of the optimization study, 1,3,5-trimethoxybenzene (50 μmol) was added to the reaction mixture as internal standard and submitted for NMR in deuterated acetonitrile. A general representation of $^1$H NMR spectra used during optimization studies is presented in Figure S6.

**General procedure for the recovery of the material after catalytic studies**

After completion of the reaction, the mixture was centrifuged at 1000 rpm for 10 min. The recovered material was washed again with water and acetone, centrifuged and the collected solid dried overnight. More than 90% of the material can be recovered.

**Synthesis of 4-azido benzonitrile**[7]

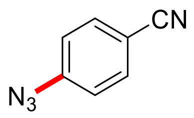


4-Aminobenzonitrile (5 mmol, 590 mg) was add to a 50 mL round bottom flask charged with a magnetic bead and purged with $N_2$. A solution of 1 M HCl (15 mL) was added, and the contents cooled to 0 $^oC$ using an ice bath. A solution of $NaNO_2$ (1.2 equiv.) in water (2mL) was added to the reaction mixture drop wise at <5 $^oC$ and stirred for 20 min. To the mixture, $NaN_3$ (1.5 equiv.) in water (2 mL) solution was added at 0 $^oC$ and the obtained suspension was stirred for 24 h. The solution was extracted with diethyl ether using separation and further washed with brine solution. The organic layer was dried over $MgSO_4$, filtered, and concentrated under reduced pressure. The residue was further purified by column chromatography (10 % EtOAc: Hexane) to give a pale-yellow powder with 86% yield (624 mg).

# 2. Computational studies

DFT calculations have been performed with the Gaussian09 software package.[8] First, geometry optimizations and frequency calculations of the ground state structure of a simplified model for the Ni-mpg-$CN_x$ system have been performed at the B3LYP/6-31G* level of theory (Figure S8).[5,9–11] Solvent effects (MeOH) are considered with the polarizable continuum model PCM-SMD of Truhlar and coworkers. [12–15] Additionally, the energy of the geometry optimized molecules was refined by single point calculation with the cc-pVTZ basis set for all atoms.[16,17]

Standard reduction potentials (E°) have been evaluated through the Nernst equation in standard state conditions using the Standard Hydrogen Electrode ($SHE$) as reference following the equation 1:

$$E^o = -\frac{\Delta G^o - \Delta G^o_{SHE}}{nF} \qquad \text{(Eq. 1)}$$

where $\Delta G^o$ is the free energy change associated with reduction at standard conditions, n is the number of electrons involved in the redox reaction, F is the Faraday constant and $\Delta G^o_{SHE}$ is the free energy change associated with the reduction of a proton (–4.28 eV).[18]

All energies given in this work are referred to Gibbs energies G in kcal $mol^{-1}$, unless otherwise noted. The nature of the stationary points was established by frequency calculations in the solvent-phase, in which minima have no imaginary frequencies.

**Redox potentials**

The possibility to have a first reduction of the Ni(II) center in Ni-mpg-$CN_x$ to Ni(I) after photoinduced electron transfer (PET) from the mpg-$CN_x$ matrix to the Ni site and then a subsequent reduction to Ni(0) by intramolecular PET was evaluated:

$$\mathbf{Ni^{II}}\text{-mpg-CN}_x \xrightarrow{E^o = 0.33\ V} \mathbf{Ni^{I}}\text{-mpg-CN}_x \xrightarrow{E^o = -1.51\ V} \mathbf{Ni^{0}}\text{-mpg-CN}_x$$

Considering that the potential of the $CN_x$ conduction band is –0.5 V vs. SHE, the reduction of Ni(II) to Ni(I) within the mpg-$CN_x$ matrix is possible (0.33 V *vs* SHE), but not a second reduction of the Ni(I) to Ni(0) (–1.51 V *vs* SHE). The calculated energy profile and the proposed catalytic cycle is represented in Figure S9-S10.

# 3. Products characterization

*The products are reported from the aryl bromide substrates, unless otherwise mentioned.*

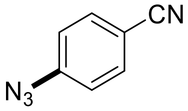

**4-azidobenzonitrile**

$^{1}H$ NMR (400 MHz, $CD_3CN$) δ 7.72 (d, *J* = 8.7 Hz, 2H), 7.19 (d, *J* = 8.7 Hz, 2H). $^{13}C$ NMR (101 MHz, $CD_3CN$) δ 146.05, 134.92, 120.85, 119.35, 108.81. IR (ATR- neat) $\tilde{\nu}$ $(cm^{-1})$ = 3223, 3095, 3043, 2432, 221, 2153, 2109, 1598, 1504, 1416, 1316, 1280, 1176,1126, 833. HRMS (ESI) calculated for $C_7H_5N_2^+$ [{(M – 2N)+H}$^+$] 117.0447, found 117.0453.

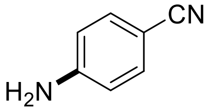

**4-Aminobenzonitrile**

The reaction was completed in 24 h and the desired product (yellow powder, 84% yield) was obtained by flash chromatography (20% ethyl acetate/hexane). $^{1}H$ NMR (400 MHz, $CDCl_3$) δ 7.41 (d, *J* = 8.7 Hz, 2H), 6.64 (d, *J* = 8.7 Hz, 2H), 4.16 (s, 2H). $^{13}C$ NMR (101 MHz, $CDCl_3$) δ 150.50, 133.95, 120.23, 114.57, 100.38. HRMS (ESI) calculated for $C_7H_7N_2^+$ [(M+H)$^+$] 119.0604, found 119.0604. Spectroscopic data were consistent with literature values.[19]

The reaction performed with 4-iodo benzonitrile was completed in 20 h. The reaction yielded 65% of the desired aniline product and the dehalogenated side product was observed with 25% NMR yield.

The reaction performed with 4-chloro benzonitrile was stopped after 60 h. The reaction proceeded to 48% conversion with 21% of the desired product. Side products resulted from hydrolysis of the nitrile group.

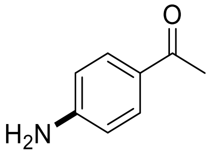

**1-(4-Aminophenyl)ethan-1-one**

The reaction was completed in 40 h and the desired product (brown powder, 83% yield) was obtained by flash chromatography (20% ethyl acetate/hexane). $^{1}H$ NMR (400 MHz, $CDCl_3$) δ 7.81 (d, *J* = 8.5 Hz, 2H), 6.65 (d, *J* = 8.5 Hz, 2H), 4.15 (s, 2H), 2.50 (s, 3H). $^{13}C$ NMR (101 MHz, $CDCl_3$) δ 196.60, 151.16, 130.93, 128.09, 113.89, 26.21. HRMS (ESI) calculated for $C_8H_{10}NO_2^+$ [(M+H)$^+$] 136.0762, found 136.0757. Spectroscopic data were consistent with literature values.[19]

The reaction performed with 4-iodo acetophenone was completed in 40 h. The reaction yielded 74% of the desired aniline product and the dehalogenated side product was observed with 20% NMR yield.

The reaction performed with 4-chloro acetophenone was stopped after 60 h. The reaction proceeded to 25% conversion with 23% of desired product.

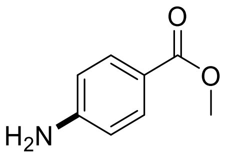 **Methyl 4-aminobenzoate**

The reaction was completed in 40 h and the desired product (brown powder, 84% yield) was obtained by flash chromatography (20% ethyl acetate/hexane). $^{1}H$ NMR (400 MHz, $CDCl_3$) δ 7.85 (d, $J$ = 8.8 Hz, 2H), 6.64 (d, $J$ = 8.8 Hz, 2H), 4.06 (s, 2H), 3.85 (s, 3H). $^{13}C$ NMR (101 MHz, $CDCl_3$) δ 167.29, 150.92, 131.74, 119.93, 113.95, 51.74. HRMS (ESI) calculated for $C_8H_{10}NO^+$ $[(M+H)^+]$ 152.0706, found 152.0700. Spectroscopic data were consistent with literature values.[19]

The reaction performed with methyl 4-Iodobenzoate was completed in 40 h. The reaction yielded 76% of the desired aniline product and the dehalogenated side product was observed with 16% NMR yield.

The reaction performed with methyl 4-chlorobenzoate was stopped after 60 h. The reaction proceeded 24% conversion with 22% of desired product.

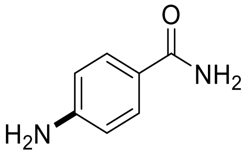 **4-aminobenzamide**

The reaction was completed in 40 h and the desired product (brown powder, 83% yield) was obtained by flash chromatography (ethyl acetate). $^{1}H$ NMR (400 MHz, DMSO-$d_6$) δ 7.57 (d, $J$ = 8.6 Hz, 2H), 7.49 (s, 1H), 6.81 (s, 1H), 6.51 (d, $J$ = 8.6 Hz, 2H), 5.62 (s, 2H). $^{13}C$ NMR (101 MHz, DMSO-$d_6$) δ 168.00, 151.47, 129.10, 121.07, 112.51. HRMS (ESI) calculated for $C_7H_9N_2O^+$ $[(M+H)^+]$ 137.0709, found 137.0705. Spectroscopic data were consistent with literature values.[7]

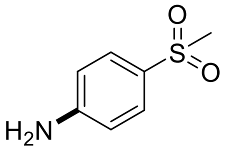 **4-(Methylsulfonyl)aniline**

The reaction was completed in 24 h and the desired product (yellow powder, 86% yield) was obtained by flash chromatography (40% ethyl acetate/hexane). $^{1}H$ NMR (400 MHz, $CDCl_3$) δ 7.67 (d, $J$ = 8.0 Hz, 2H), 6.70 (d, $J$ = 8.0 Hz, 2H), 4.26 (s, 2H), 3.00 (s, 3H). $^{13}C$ NMR (101 MHz, $CDCl_3$) δ 151.46, 129.44, 128.66, 114.10, 45.00. HRMS (ESI) calculated for $C_7H_{10}NO_2S^+$ $[(M+H)^+]$ 172.0427, found 172.0417. Spectroscopic data were consistent with literature values.[20]

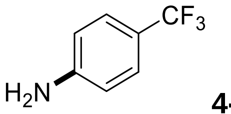 **4-(Trifluoromethyl)aniline**

The reaction was completed in 40 h and the desired product (brown powder, 65% yield) was obtained by flash chromatography (20% ethyl acetate/hexane). $^{1}H$ NMR (400 MHz, $CDCl_3$) δ 7.42 (d, $J$ = 8.6 Hz, 2H), 6.65 (d, $J$ = 8.6 Hz, 2H), 4.13 (s, 2H). $^{13}C$ NMR (101 MHz, $CDCl_3$) δ

150.42, 133.98, 120.19, 114.59, 100.55. HRMS (ESI) calculated for $C_7H_7F_3N^+$ $[(M+H)^+]$ 162.0525, found 162.0522.

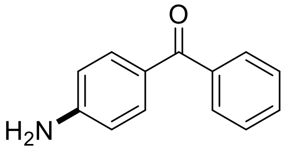


**4-Aminobenzophenone**

The reaction was completed in 40 h and the desired product (brown powder, 75% yield) was obtained by flash chromatography (20% ethyl acetate/hexane). $^1H$ NMR (400 MHz, $CDCl_3$) δ 7.74 – 7.71 (m, 4H), 7.54 (t, $J$ = 7.4 Hz, 1H), 7.45 (t, $J$ = 7.4 Hz, 2H), 6.70 (d, $J$ = 8.6 Hz, 2H), 4.31 (s, 2H). $^{13}C$ NMR (101 MHz, $CDCl_3$) δ 195.47, 150.65, 138.95, 133.06, 131.59, 129.68, 128.22, 127.84, 114.01. HRMS (ESI) calculated for $C_{13}H_{12}NO^+$ $[(M+H)^+]$ 198.0919, found 198.0919. Spectroscopic data were consistent with literature values.[19]

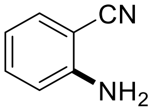


**2-Aminobenzonitrile**

The reaction was completed in 40 h and the desired product (brown powder, 76% yield) was obtained by flash chromatography (10% ethyl acetate/hexane). $^1H$ NMR (400 MHz, $CDCl_3$) δ 7.38 (dd, $J$ = 8.0, 1.6 Hz, 1H), 7.32 (ddd, $J$ = 8.4, 7.4, 1.6 Hz, 1H), 6.77 – 6.69 (m, 2H), 4.39 (s, 2H). $^{13}C$ NMR (101 MHz, $CDCl_3$) δ 149.71, 134.13, 132.48, 118.13, 117.73, 115.27, 96.18. HRMS (ESI) calculated for $C_7H_7N_2^+$ $[(M+H)^+]$ 119.0604, found 119.0600.

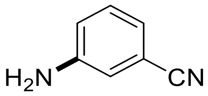


**3-Aminobenzonitrile**

The reaction was completed in 40 h and the desired product (brown powder, 74% yield) was obtained by flash chromatography (20% ethyl acetate/hexane). $^1H$ NMR (400 MHz, $CDCl_3$) δ 7.21 (dd, 1H), 7.00 (m, 1H), 6.89 (m,1H), 6.86 (m, 1H), 3.89 (s, 2H). $^{13}C$ NMR (101 MHz, $CDCl_3$) δ 147.08, 130.17, 122.07, 119.31,119.30 117.54, 113.02. HRMS (ESI) calculated for $C_7H_7N_2^+$ $[(M+H)^+]$ 119.0604, found 119.0599.

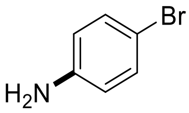


**4-Bromoaniline**

The reaction was stopped after 60 h and, 68% conversion was observed in proton NMR spectroscopy. The desired product (white powder, 56% yield) was obtained by flash chromatography (20% ethyl acetate/hexane). $^1H$ NMR (400 MHz, $CDCl_3$) δ 7.23 (d, $J$ = 8.6 Hz, 2H), 6.56 (d, $J$ = 8.6 Hz, 2H), 3.67 (s, 2H). $^{13}C$ NMR (101 MHz, $CDCl_3$) δ 145.52, 132.16, 116.85, 110.38. HRMS (ESI) calculated for $C_7H_7BrN^+$ $[(M+H)^+]$ 171.976 , found 171.9752. Spectroscopic data were consistent with literature values.[19]

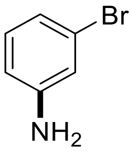


**3-Bromoaniline**

The reaction was stopped after 60 hours, and 80% conversion was observed in proton NMR spectroscopy. The desired product (yellow viscous liquid, 69% yield) was obtained by flash chromatography (15% ethyl acetate/hexane). $^{1}$H NMR (400 MHz, $CDCl_3$) δ 7.00 (dd, 1H), 6.91 – 6.79 (m, 2H), 6.61 – 6.57 (m, 1H), 3.70 (s, 2H). $^{13}$C NMR (101 MHz, $CDCl_3$) δ 147.89, 130.72, 123.15, 121.50, 117.95, 113.76. HRMS (ESI) calculated for $C_6H_7NBr^+$ [$(M+H)^+$] 171.9756, found 171.9761.

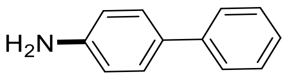


**4-Aminobiphenyl**

The reaction was completed in 60 h and the desired product (white powder, 72% yield) was obtained by flash chromatography (20% ethyl acetate/hexane). $^{1}$H NMR (400 MHz, $CDCl_3$) δ 7.54 (d, *J* = 7.9 Hz, 2H), 7.45 – 7.37 (m, 4H), 7.31 – 7.26 (m, 1H), 6.77 (d, *J* = 8.4 Hz, 2H), 3.73 (s, 2H). $^{13}$C NMR (101 MHz, $CDCl_3$) δ 145.96, 141.29, 131.72, 128.79, 128.15, 126.54, 126.39, 115.52. HRMS (ESI) calculated for $C_{12}H_{12}N^+$ [$(M+H)^+$] 170.0964, found 170.0959. Spectroscopic data were consistent with literature values.[19]

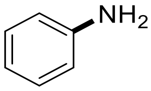


**Aniline**

The reaction was stopped after 60 hours, and 60% conversion was observed with 56% of the desired product in proton NMR spectroscopy. The product was obtained by flash chromatography (15% ethyl acetate/hexane). $^{1}$H NMR (400 MHz, $CDCl_3$) δ 7.21 – 7.12 (m, 2H), 6.82 – 6.75 (m, 1H), 6.73 – 6.63 (m, 2H), 3.65 (s, 2H). $^{13}$C NMR (101 MHz, $CDCl_3$) δ 146.49, 129.40, 118.66, 115.21. HRMS (ESI) calculated for $C_6H_8N^+$ [$(M+H)^+$ 94.0651, found 94.0653.

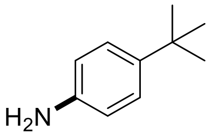


**4-(tert-butyl)aniline**

The reaction was stopped after 60 h and, 33% conversion was observed in proton NMR spectroscopy. The desired product (brown oil, 26% yield) was obtained by flash chromatography (20% ethyl acetate/hexane). $^{1}$H NMR (400 MHz, $CDCl_3$) δ 7.20 (d, *J* = 7.6 Hz, 2H), 6.71 (d, *J* = 7.6 Hz, 2H), 3.89 (s, 2H), 1.28 (s, 9H). $^{13}$C NMR (101 MHz, $CDCl_3$) δ 155.19, 142.46, 126.27, 115.70, 34.12, 31.65. HRMS (ESI) calculated for $C_{10}H_{16}N^+$ [$(M+H)^+$] 150.1283, found 150.1284. Spectroscopic data were consistent with literature values.[19]

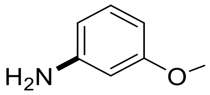


**3-methoxyaniline**

The reaction was stopped after 60 h and, 50% conversion was observed in proton NMR spectroscopy. The desired product (brown powder, 46% yield) was obtained by flash chromatography (20% ethyl acetate/hexane). $^{1}$H NMR (400 MHz, $CDCl_3$) δ 7.08 (dd, 1H), δ 6.41 – 6.25 (m, 3H), 3.77 (s, 3H), 3.77 (s, 2H). $^{13}$C NMR (101 MHz, $CDCl_3$) δ 160.90, 147.46, 130.27, 108.25, 104.40, 101.43, 55.24. HRMS (ESI) calculated for $C_7H_{10}NO^+$ [$(M+H)^+$] 124.0762, found 124.0760. Spectroscopic data were consistent with literature values.[19]

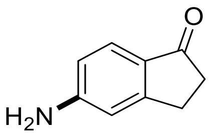

**5-Amino-2,3-dihydro-1H-inden-1-one**

The reaction was completed in 40 hours and the desired product (brown powder, 54% yield) was obtained by flash chromatography (20% ethyl acetate/hexane). $^{1}$H NMR (400 MHz, $CDCl_3$) δ 7.57 (d, *J* = 8.4 Hz, 1H), 6.61 (s, 1H), 6.60 (d, *J* = 8.4 Hz, 1H), 4.24 (s, 2H), 3.03 – 2.93 (m, 2H), 2.74 – 2.50 (m, 2H). $^{13}$C NMR (101 MHz, $CDCl_3$) δ 204.99, 158.50, 152.96, 128.19, 125.85, 114.73, 110.06, 36.48, 25.76. HRMS (ESI) calculated for $C_9H_{10}NO^+$ [$(M+H)^+$] 148.0757, found 148.0752.

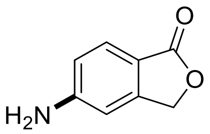

**5-Aminoisobenzofuran-1(3H)-one**

The reaction was completed in 40 hours and the desired product (brown powder, 56% yield) was obtained by flash chromatography (30% ethyl acetate/hexane). $^{1}$H NMR (400 MHz, Acetone-*d*$_6$) δ 7.49 (d, *J* = 8.4 Hz, 1H), 6.80 (d, *J* = 8.4 Hz, 1H), 6.73 (s, 1H), 5.13 (s, 2H), 2.82 (s, 2H). $^{13}$C NMR (101 MHz, Acetone-*d*$_6$) δ 171.42, 155.19, 151.05, 127.14, 115.91, 113.88, 105.87, 69.37. HRMS (ESI) calculated for $C_8H_8NO_2^+$ [$(M+H)^+$] 150.0550, found 150.0545.

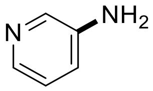

**Pyridin-3-amine**

The reaction was completed in 40 hours and the desired product (brown powder, 87% yield) was obtained by flash chromatography (2% methanol/ethyl acetate).$^{1}$H NMR (400 MHz, $CDCl_3$) δ 8.09 (s, 1H), 8.00 (d, *J* = 4.6 Hz, 1H), 7.05 (dd, *J* = 8.1, 4.6 Hz, 1H), 6.97 – 6.94 (m, 1H), 3.72 (s, 2H).$^{13}$C NMR (101 MHz, $CDCl_3$) δ 142.68, 139.98, 137.49, 123.85, 121.61. HRMS (ESI) calculated for $C_9H_{10}NO^+$ [$(M+H)^+$] 95.0604, found 95.06039. Spectroscopic data were consistent with literature values.[19]

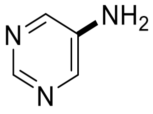

**Pyrimidin-5-amine**

The reaction was completed in 24 hours and the desired product (brown powder, 67% yield) was obtained by flash chromatography (2% methanol/ethyl acetate). $^{1}$H NMR (400 MHz, $CDCl_3$) δ 8.64 (s, 1H), 8.20 (s, 2H), 3.59 (s, 2H). $^{13}$C NMR (101 MHz, $CDCl_3$) δ 149.77, 143.05, 140.63. HRMS (ESI) calculated for $C_9H_{10}NO^+$ [$(M+H)^+$] 96.0556, found 96.0556.

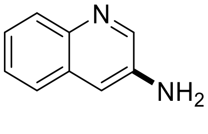

**Quinolin-3-amine**

The reaction was completed in 40 hours and the desired product (brown powder, 70% yield) was obtained by flash chromatography (60% ethyl acetate/hexane). $^{1}$H NMR (400 MHz, $CDCl_3$) δ 8.50 (s, 1H), 7.96 (d, *J* = 7.3 Hz, 1H), 7.57 (d, *J* = 8.7 Hz, 1H), 7.45 – 7.39 (m, 2H), 7.21 (s, 1H), 3.93 (s, 2H). $^{13}$C NMR (101 MHz, $CDCl_3$) δ 143.17, 142.76, 139.89, 129.27,

129.10, 127.07, 125.97, 125.72, 115.11. HRMS (ESI) calculated for $C_9H_9N_2^+$ [(M+H)$^+$] 145.0760, found 145.0756. Spectroscopic data were consistent with literature values.[19]

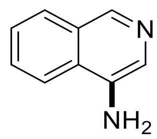


**Isoquinolin-4-amine**

The reaction was completed in 60 hours and the desired product (yellow powder, 64% yield) was obtained by flash chromatography (80% ethyl acetate/hexane). $^1$H NMR (400 MHz, $CDCl_3$) δ 8.75 (s, 1H), 8.04 (s, 1H), 7.92 (d, *J* = 8.2 Hz, 1H), 7.81 (d, *J* = 8.4 Hz, 1H), 7.66 (dd, 1H), 7.58 (dd,1H), 4.10 (s, 2H). $^{13}$C NMR (101 MHz, $CDCl_3$) δ 143.47, 136.88, 129.19, 128.78, 128.33, 127.98, 127.21, 126.26, 120.13. HRMS (ESI) calculated for $C_9H_9N_2^+$ [(M+H)$^+$] 145.0760, found 145.0763.

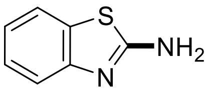


**Benzothiazol-2-amine**

The reaction was completed in 40 hours and the desired product (pale yellow powder, 61% yield) was obtained by flash chromatography (30% ethyl acetate/hexane). $^1$H NMR (400 MHz, $CDCl_3$) δ 7.58 (d, *J* = 8.0 Hz, 1H), 7.53 (d, *J* = 8.0 Hz, 1H), 7.30 (t, *J* = 8.0 Hz, 1H), 7.12 (t, *J* = 8.0 Hz, 1H), 5.70 (s, 2H). $^{13}$C NMR (101 MHz, $CDCl_3$) δ 166.26, 152.14, 131.65, 126.10, 122.39, 121.04, 119.24. HRMS (ESI) calculated for $C_7H_7N_2S^+$ [(M+H)$^+$] 151.0324, found 151.0328.

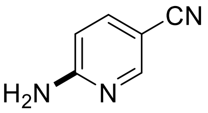


**6-Aminonicotinonitrile**

The reaction was completed in 50 hours and the desired product (white powder, 74% yield) was obtained by flash chromatography (80% ethyl acetate/hexane). $^1$H NMR (400 MHz, DMSO-$d_6$) δ 8.26 (d, *J* = 2.3 Hz, 1H), 7.63 (dd, *J* = 8.8, 2.3 Hz, 1H), 6.96 (s, 2H), 6.44 (d, *J* = 8.8 Hz, 1H). $^{13}$C NMR (101 MHz, DMSO-$d_6$) δ 161.60, 153.26, 139.39, 119.05, 107.81, 94.63. HRMS (ESI) calculated for $C_6H_6N_3^+$ [(M+H)$^+$] 120.0556, found 120.0552.

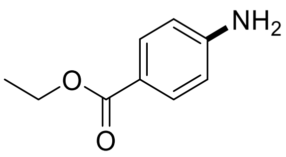


**Ethyl 4-aminobenzoate**

The reaction employed ethanol as a solvent instead of methanol and was completed in 50 hours. The desired product (white powder, 83% yield) was obtained by flash chromatography (20% ethyl acetate/hexane). $^1$H NMR (400 MHz, $CDCl_3$) δ 7.85 (d, *J* = 8.6 Hz, 2H), 6.63 (d, *J* = 8.6 Hz, 2H), 4.31 (q, *J* = 7.1 Hz, 2H), 4.07 (s, 2H), 1.35 (t, *J* = 7.1 Hz, 3H). $^{13}$C NMR (101 MHz, $CDCl_3$) δ 166.84, 150.90, 131.65, 120.16, 113.87, 60.41, 14.53. HRMS (ESI) calculated for $C_9H_{12}O_2N^+$ [(M+H)$^+$] 166.0863, found 166.0870.

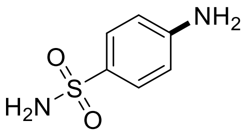

**4-Aminobenzenesulfonamide**

The reaction was completed in 50 hours and the desired product (white powder, 85% yield) was obtained by flash chromatography (ethyl acetate). $^{1}$H NMR (400 MHz, DMSO-$d_6$) δ 7.41 (d, $J$ = 8.7 Hz, 2H), 6.84 (s, 2H), 6.55 (d, $J$ = 8.7 Hz, 2H), 5.75 (s, 2H). $^{13}$C NMR (101 MHz, DMSO-$d_6$) δ 151.94, 130.05, 127.45, 112.47. HRMS (ESI) calculated for $C_6H_8N_2NaS^+$ [(M+Na)$^+$] 195.0199, found 195.0201.

## 4. Supporting Tables

**Table S1.** ICP-OES data for different Ni deposited carbon nitrides.

| Material | ICP-OES Ni content (wt %) |
|---|---|
| Ni-mpg-$CN_x$ #13 (used for initital optimization and scope) | 5.96 |
| Ni-$CN_x$ | 3.88 |
| Ni-$^{NCN}CN_x$ | 2.60 |
| Ni-mpg-$CN_x$ #14 (used for scope and recycling studies) | 6.98 |
| Ni-mpg-$CN_x$ #14 (recycled 1) | 6.68 |
| Ni-mpg-$CN_x$ #14 (recycled 2) | 5.75 |
| Ni-mpg-$CN_x$ #14 (recycled 3) | 5.74 |
| Ni-mpg-$CN_x$ #14 (recycled 4) | 5.39 |
| Ni-mpg-$CN_x$ #14 (reaction in EDTA) | 0.54 |

**Table S2.** Screening of different solvents.

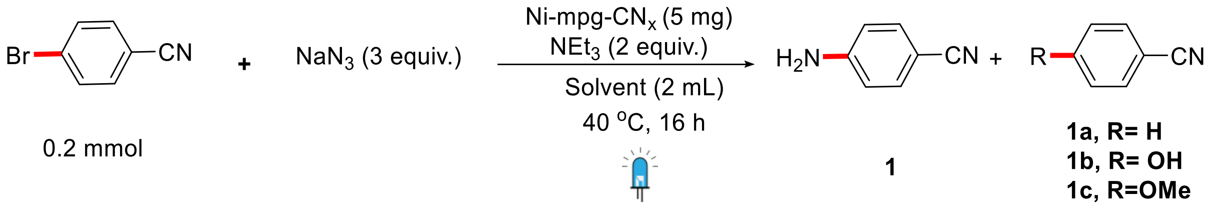


| Entry | Condition | Conversion | 1 | 1a | 1b | 1c |
|---|---|---|---|---|---|---|
| 1 | $CH_3CN$: $H_2O$ (3:1) | 7 | 6 | traces | n.d. | n.d. |
| 2 | $CH_3CN$: $H_2O$ (5:3) | 22 | 17 | <5 | traces | n.d. |
| 3 | $CH_3CN$: $H_2O$ (1:1) | 26 | 18 | <5 | 5 | n.d. |
| 4 | DME: $H_2O$ (5:3) | 21 | 15 | 6 | n.d. | n.d. |
| 5 | THF: $H_2O$ (5:3) | 9 | 7 | traces | n.d. | n.d. |
| 6 | Dioxane: $H_2O$ (5:3) | 27 | 20 | 5 | traces | n.d. |
| 7 | DMA: $H_2O$ (5:3) | 37 | 22 | 10 | 5 | n.d. |
| 8 | DMSO: $H_2O$ (5:3) | 59 | 32 | 17 | 10 | n.d. |
| 9 | DMSO | 31 | 23 | 5 | traces | n.d. |
| 10 | Ethanol: $H_2O$ (5:3) | 32 | 24 | 5 | traces | n.d. |
| 11 | Ethanol: $H_2O$ (3:1) | 27 | 18 | <5 | <5 | n.d. |
| 12 | Ethanol: $H_2O$ (1:1) | 23.5 | 18 | <5 | traces | n.d. |
| 13 | Ethanol | 12 | 10 | traces | n.d. | n.d. |
| 14 | Methanol: $H_2O$ (5:3) | 40 | 29 | 5 | <5 | traces |
| 15 | Methanol: $H_2O$ (3:1) | 38 | 26 | 8 | <5 | traces |
| 16 | Methanol: $H_2O$ (1:1) | 39 | 27 | <5 | 7 | n.d. |
| 17 | Methanol | 31 | 19 | 7 | <5 | traces |

Reaction conditions: 4-bromobenzonitrile = 0.2 mmol (100 mM), Ni-mpg- $CN_x$ = 5 mg, sodium azide = 0.6 mmol, triethyl amine = 0.4 mmol in different solvent solution (2 mL), 16 h irradiation at $\lambda$ = 447 ± 20 nm and 40 ºC under $N_2$. Note: All the yields are determined by $^1$H-NMR using 1,3,5-trimethoxybenzene (50 μmol) as internal standard

**Table S3.** Screening of different bases.

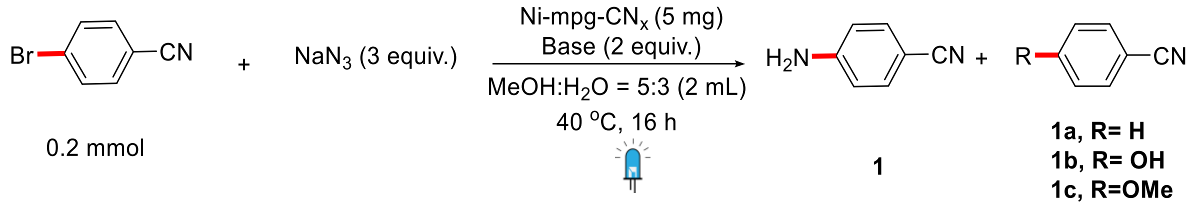


| Entry | Condition | Conversion | 1 | 1a | 1b | 1c |
|---|---|---|---|---|---|---|
| 1 | Triethyl amine | 40 | 29 | 5 | <5 | traces |
| 2 | DABCO | 15 | 12 | traces | traces | n.d. |
| 3 | DBU | 56 | 15 | 15 | 19 | 7 |
| 4 | *N, N*-Diisopropylethylamine | 46 | 28 | 8 | 8 | traces |
| 5 | N-*tert*-butylisopropylamine | 32 | 19 | 6 | 5 | traces |
| 6 | $Na_2CO_3$ | 19 | 14 | traces | traces | n.d. |
| 7 | $K_3PO_4$ | n.d. | n.d. | n.d. | n.d. | n.d. |
| 8 | No base | 16 | 13 | traces | traces | n.d. |
| 9 | DBU[a] | 13 | 7 | 6 | n.d. | n.d. |
| 10 | DBU[b] | 24 | 6 | 10 | n.d. | 8 |

Reaction conditions: 4-bromobenzonitrile = 0.2 mmol (100 mM), Ni-mpg- $CN_x$ = 5 mg, sodium azide = 0.6 mmol, base = 0.4 mmol in methanol: water (5:3) solvent solution (2 mL), 16 h irradiation at $\lambda$ = 447 ± 20 nm and 40 °C under $N_2$. Note: All the yields are determined by $^1$H-NMR using 1,3,5-trimethoxybenzene (50 μmol) as internal standard. [a] reaction performed in DMSO, [b] reaction is performed in ethanol. Abbreviations: DABCO is 1,4-diazabicyclo[2.2.2]octane; DBU is 1,8-Diazabicyclo[5.4.0]undec-7-ene.

**Table S4.** Varying amount of sodium azide and triethylamine

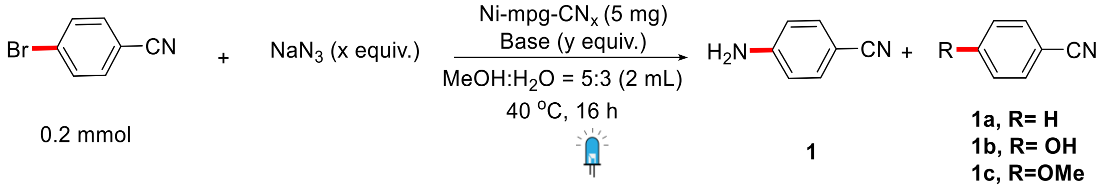


| Entry | Amount | Conversion | 1 | 1a | 1b | 1c |
|---|---|---|---|---|---|---|
| 1[a] | $Et_3N$ (2 eq) | 40 | 29 | 5 | <5 | traces |
| 2[a] | $Et_3N$ (3 eq) | 41 | 27 | 7 | 5 | traces |
| 3[a] | $Et_3N$ (5 eq) | 42 | 26 | 8 | 7 | traces |
| 4[b] | $NaN_3$ (2 eq) | 32 | 22 | 5 | <5 | traces |
| 5[b] | $NaN_3$ (3 eq) | 40 | 29 | 5 | <5 | traces |
| 6[b] | $NaN_3$ (5 eq) | 42 | 32 | 6 | <5 | traces |

Reaction conditions: 4-bromobenzonitrile = 0.2 mmol (100 mM), Ni-mpg- $CN_x$ = 5 mg in methanol: water (5:3) solvent solution (2 mL), 16 h irradiation at $\lambda$ = 447 ± 20 nm and 40 °C under $N_2$. Note: All the yields are determined by $^1$H-NMR using 1,3,5-trimethoxybenzene (50 μmol) as internal standard. [a] reaction performed with sodium azide = 0.6 mmol (3 equiv.), [b] reaction is performed with triethyl amine ($Et_3N$) = 0.4 mmol (2 equiv.).

**Table S5.** Screening of different temperature

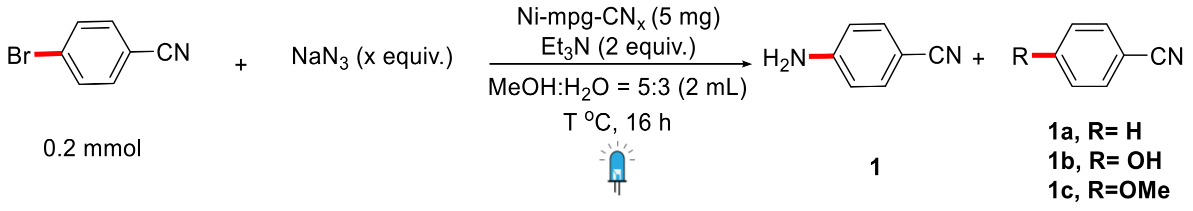

| Entry | Temperature | Conversion | 1 | 1a | 1b | 1c |
|---|---|---|---|---|---|---|
| 1 | 50 °C with $NaN_3$ (3 eq) | 54.5 | 38 | 8 | 7 | traces |
| 2 | 50 °C with $NaN_3$ (5 eq) | 59.5 | 46 | 7 | 5 | traces |
| 3 | 60 °C with $NaN_3$ (3 eq) | 66 | 49 | 8 | 7 | traces |
| 4 | 60 °C with $NaN_3$ (5 eq) | 83 | 70 | 5 | 7 | traces |
| 5[a] | 60 °C with $NaN_3$ (5 eq) | 77 | 65 | 5 | 5 | traces |

Reaction conditions: 4-bromobenzonitrile = 0.2 mmol (100 mM), Ni-mpg- $CN_x$ = 5 mg, sodium azide = x equiv., triethyl amine = 0.4 mmol in methanol: water (5:3) solvent solution (2 mL), 16 h irradiation at $\lambda$ = 447 ± 20 nm and 40 °C under $N_2$. Note: All the yields are determined by $^1$H-NMR using 1,3,5-trimethoxybenzene (50 μmol) as internal standard. [a] reaction performed with 4-bromobenzonitrile = 0.4 mmol (200 mM), Ni-mpg- $CN_x$ = 10 mg, sodium azide = 2 mmol (5 equiv.), triethyl amine = 0.8 mmol (2 equiv.) in methanol: water (5:3) solvent solution (2 mL).

**Table S6.** Varying amount of photocatalyst

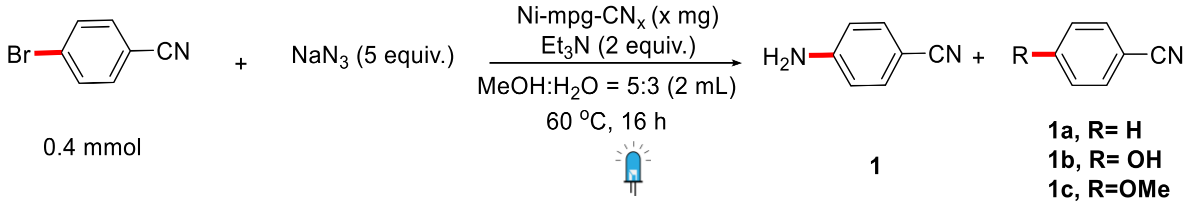

| Entry | amount | Conversion | 1 | 1a | 1b | 1c |
|---|---|---|---|---|---|---|
| 1 | 5 mg | 62 | 53 | <5 | <5 | traces |
| 2 | 8 mg | 72 | 61 | 5 | <5 | traces |
| 3 | 10 mg | 77 | 65 | 5 | 5 | traces |
| 4 | 12 mg | 80 | 68 | 6 | 5 | traces |

Reaction conditions: 4-bromobenzonitrile = 0.4 mmol (200 mM), Ni-mpg- $CN_x$ = x mg, sodium azide = 2 mmol (5 equiv.), triethyl amine = 0.8 mmol (2 equiv.) in methanol: water (5:3) solvent solution (2 mL), 16 h irradiation at $\lambda$ = 447 ± 20 nm and 60 °C under $N_2$. Note: All the yields are determined by $^1$H-NMR using 1,3,5-trimethoxybenzene (50 μmol) as internal standard.

**Table S7.** Investigation of aryl azide for aniline formation.

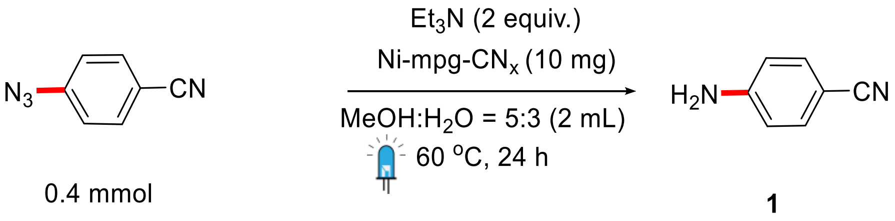


| Entry | Condition | Conversion | Product (1) |
|---|---|---|---|
| **1** | standard | 95% | 93% |
| **2** | No light | 16% | 4% |
| **3** | No Ni-mpg-$CN_x$ | 90% | 20% |
| **4** | $CH_3CN$ instead of MeOH | 5% | 3% |

Reaction conditions: 4-azidobenzonitrie = 0.4 mmol (200 mM), Ni-mpg- $CN_x$ = 10 mg, triethyl amine = 0.8 mmol (2 equiv.) in methanol: water (5:3) solvent solution (2 mL), 24 h irradiation at $\lambda$= 447 ± 20 nm and 60 ºC under $N_2$, unless otherwise mentioned. Note: All the yields are determined by $^1$H-NMR using 1,3,5-trimethoxybenzene as internal standard.

**Table S8.** Investigation of photocatalytic amination in different conditions.

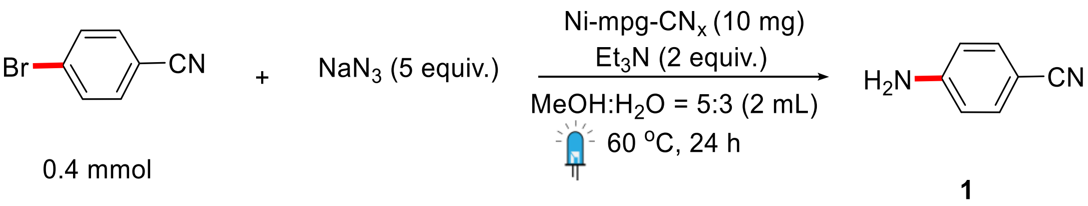


| Entry | Condition | Conversion | Product (1) | $H_2$ (mmol) |
|---|---|---|---|---|
| **1** | MeO**D**:$H_2O$ | 89% | 71% | - |
| **2** | MeOH:**D**$_2$O | 91% | 75% | - |
| **3** | MeO**D**:**D**$_2$O | 91% | 91% | - |
| **4** | $MeOH:H_2O$ | 93% | 88% | 0.12 |
| **5** | $MeOH:H_2O$(no substrate) | - | - | 4.27 |

Reaction conditions: 4-bromobenzonitrile = 0.4 mmol (200 mM), Ni-mpg- $CN_x$ = 10 mg, sodium azide = 2 mmol (5 equiv.), triethyl amine = 0.8 mmol (2 equiv.) in methanol: water (5:3) solvent solution (2 mL), 24 h irradiation at $\lambda$ = 447 ± 20 nm and 60 ºC under $N_2$, unless otherwise noted. Note: All the yields are determined by $^1$H-NMR using 1,3,5-trimethoxybenzene (50 µmol) as internal standard.

## 5. Supporting Figures

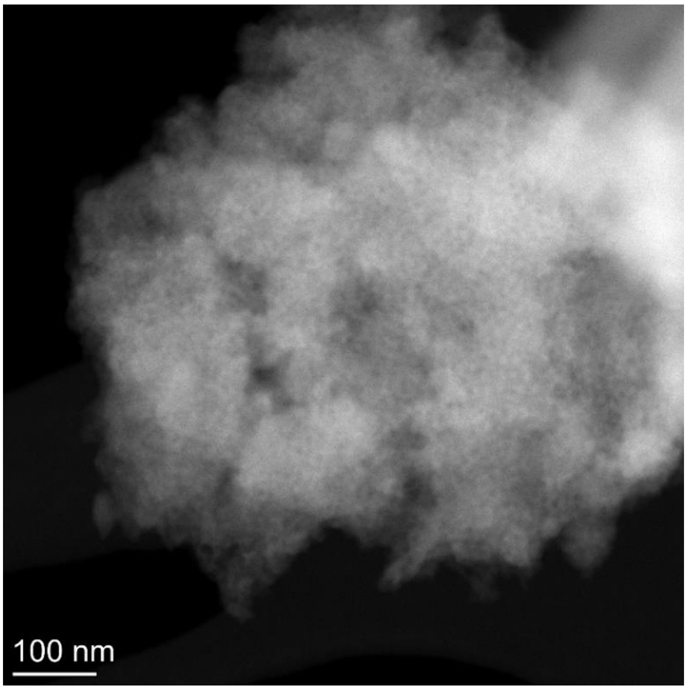


**Figure S1.** TEM image of Ni-mpg-$CN_x$.

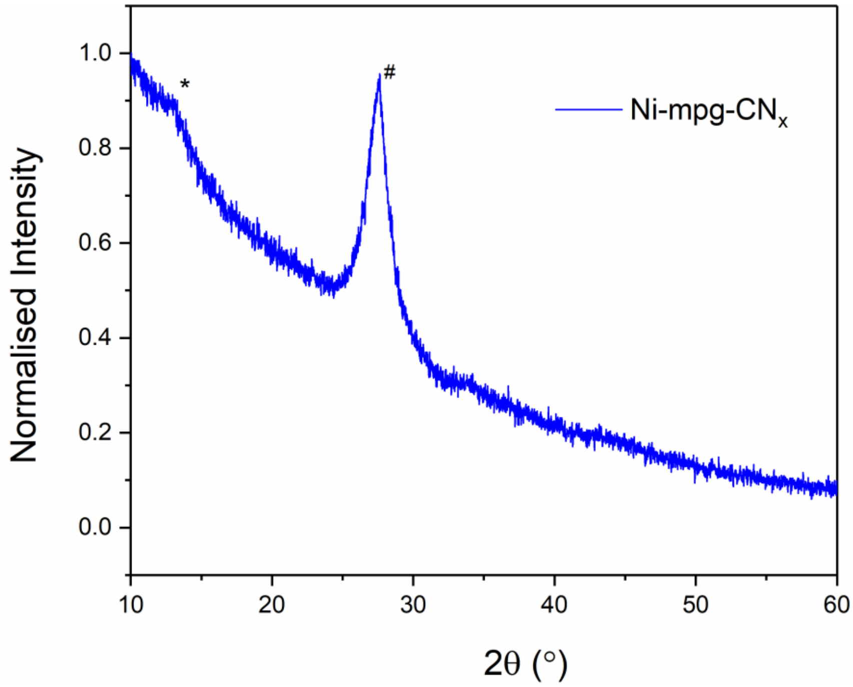


**Figure S2.** pXRD spectra of Ni-mpg-$CN_x$. The peaks present at 27° and 12° confirm the stacking and in-plane periodicity, respectively.[5]

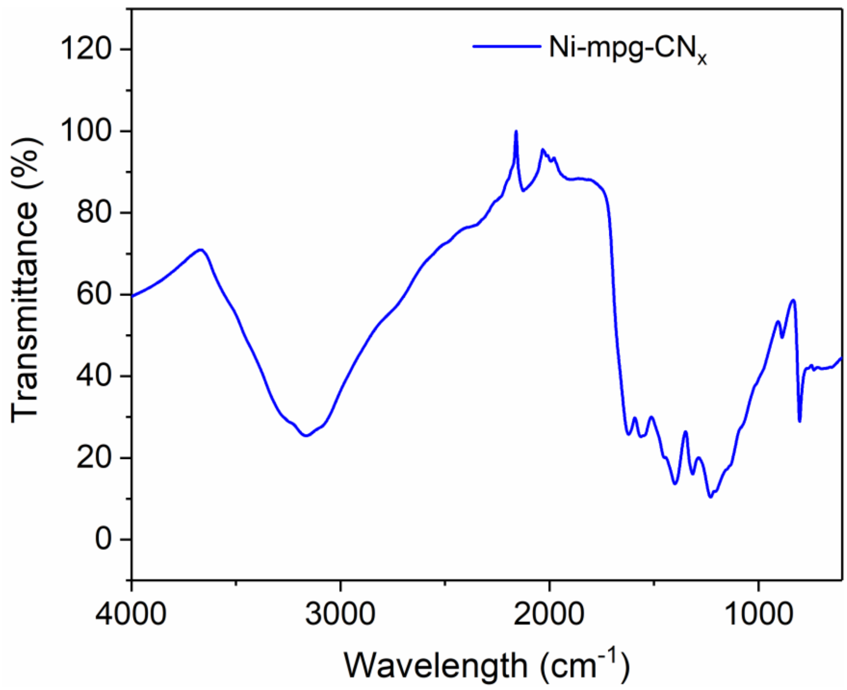


**Figure S3.** ATR-IR spectra of Ni-mpg-$CN_x$. The peak at 804 $cm^{-1}$ is consistent with the characteristic vibration of the heptazine core.

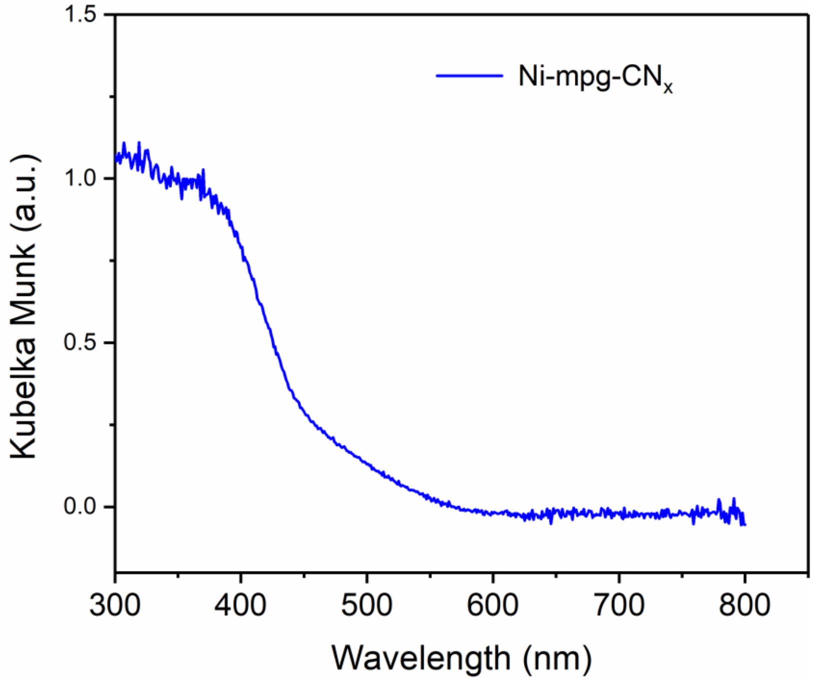


**Figure S4.** UV-Vis DRS spectrum of Ni-mpg-$CN_x$.

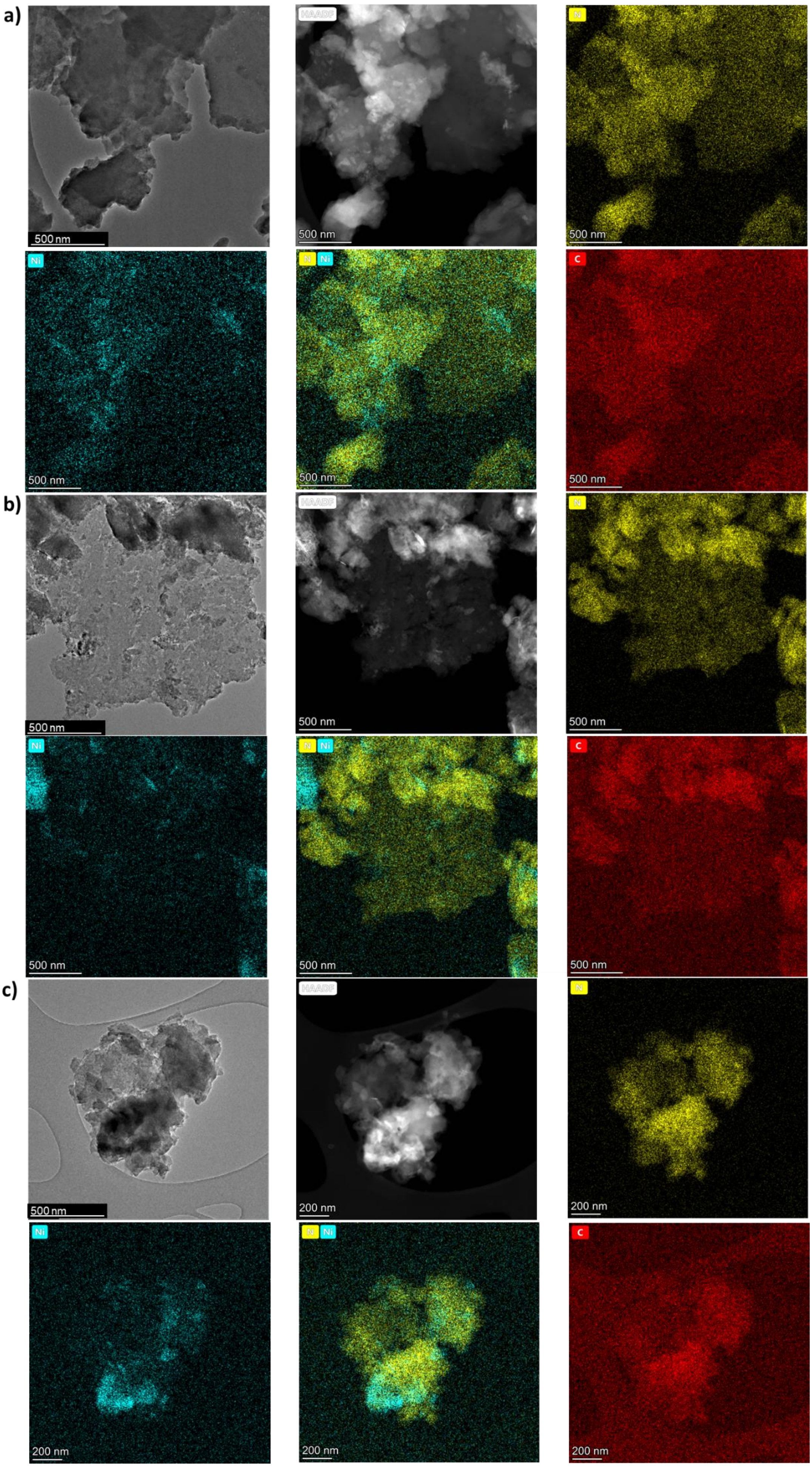
a)
b)
c)
HAADF
N
Ni
C
500 nm
200 nm

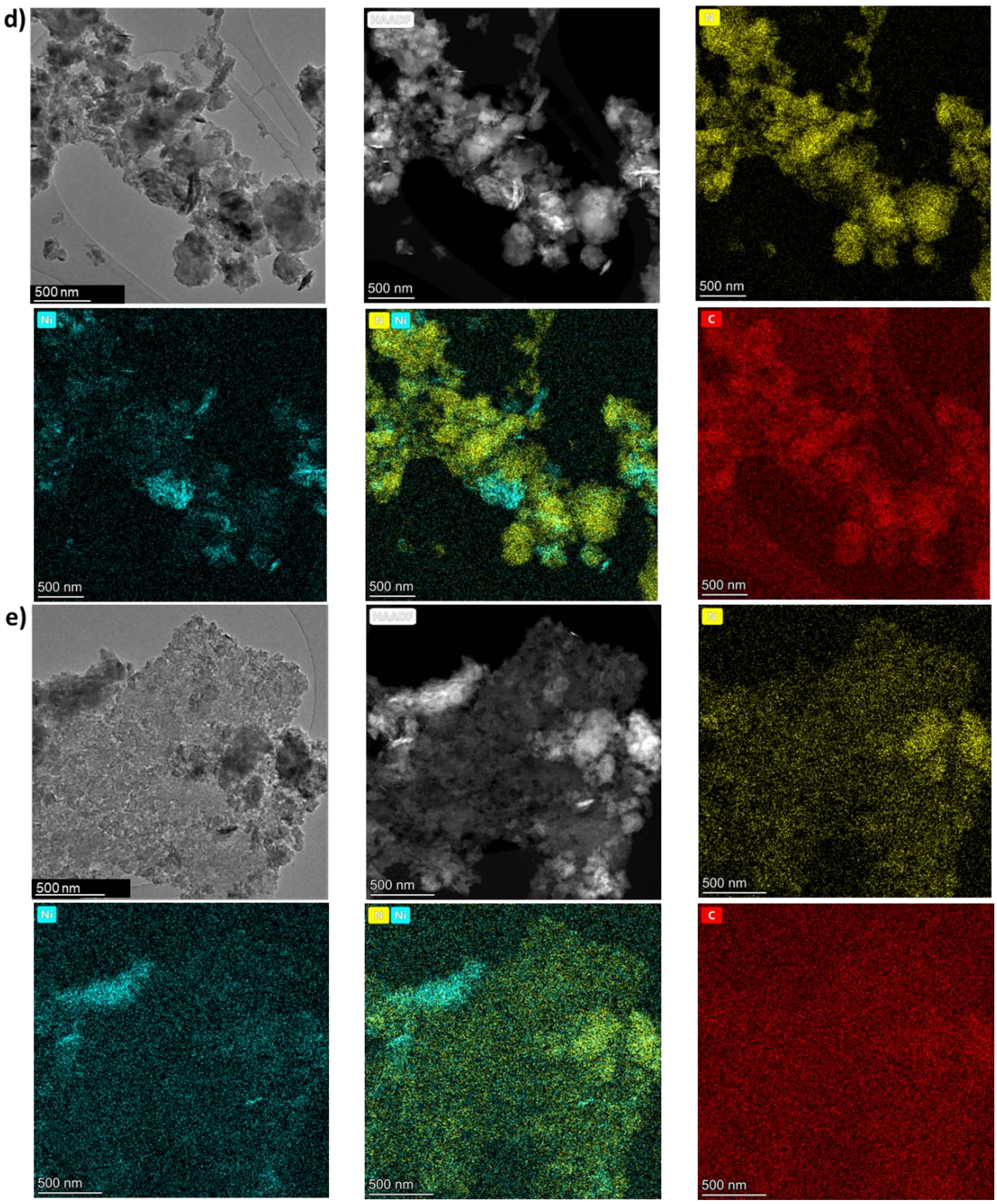


**Figure S5.** TEM and HAADF-STEM images and EDS mapping of the material before catalysis (a), and after each recycling cycle (1, *b;* 2, *c;* 3, *d;* 4, *e*).

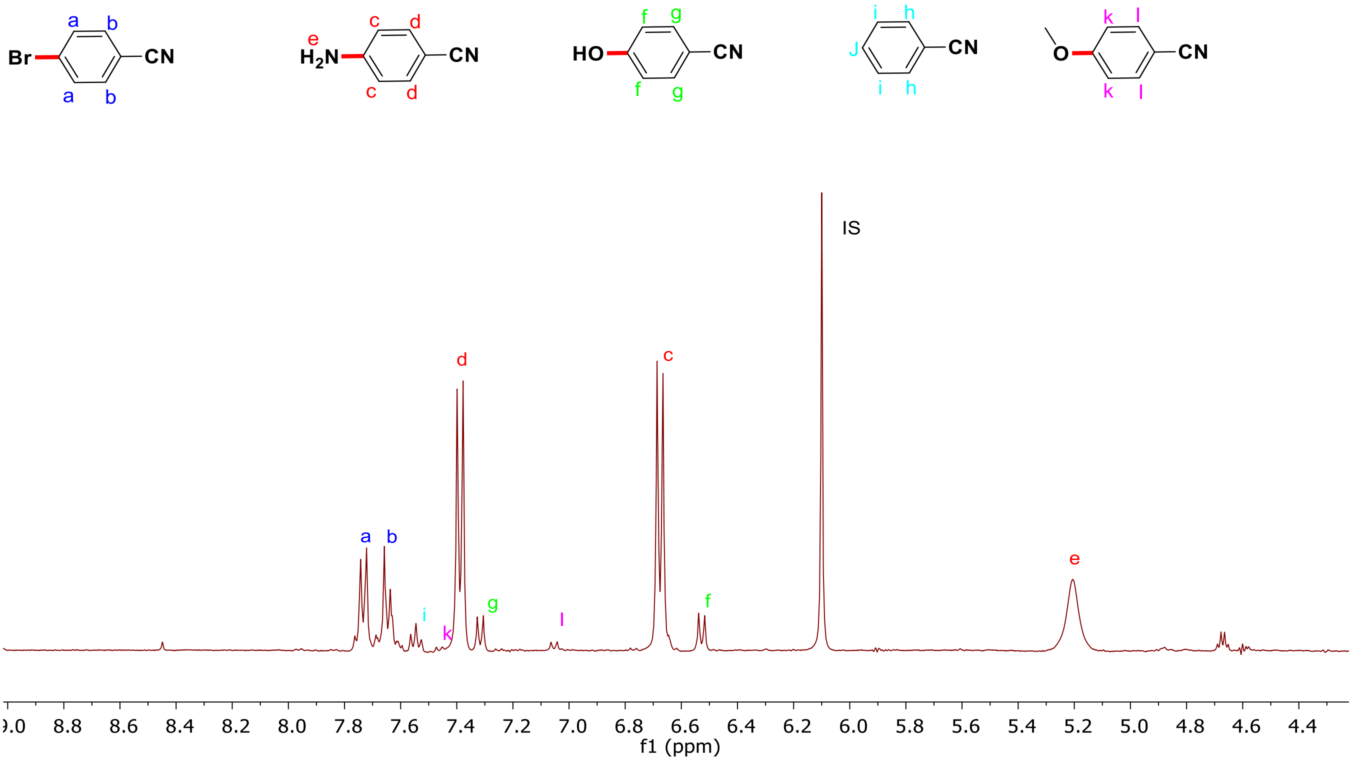


**Figure S6.** Representative $^1$H-NMR spectrum of reaction mixture. 1,3,5-trimethoxybenzene was used as an internal standard. Peak for the postulated aryl azide intermediate is absent (see text).

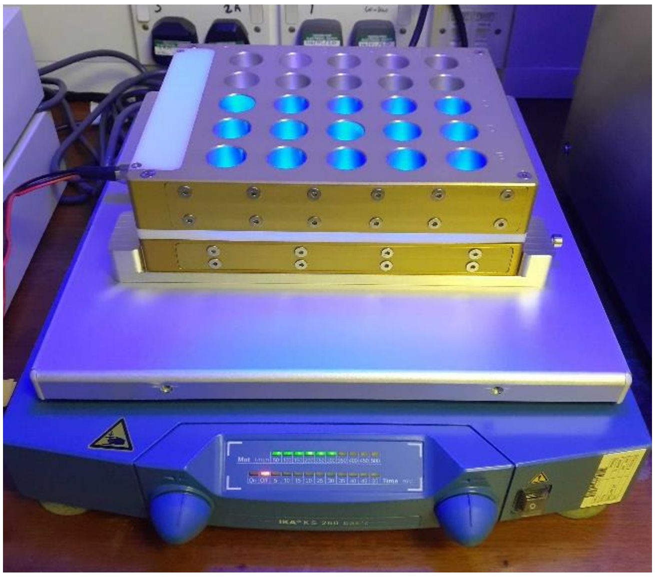

**Figure S7.** Representation of the photoreactor and setup used for the photocatalytic experiments.

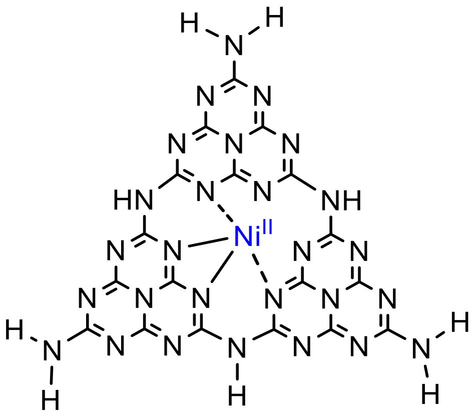

**Figure S8.** Simplified model system used for the computational studies.

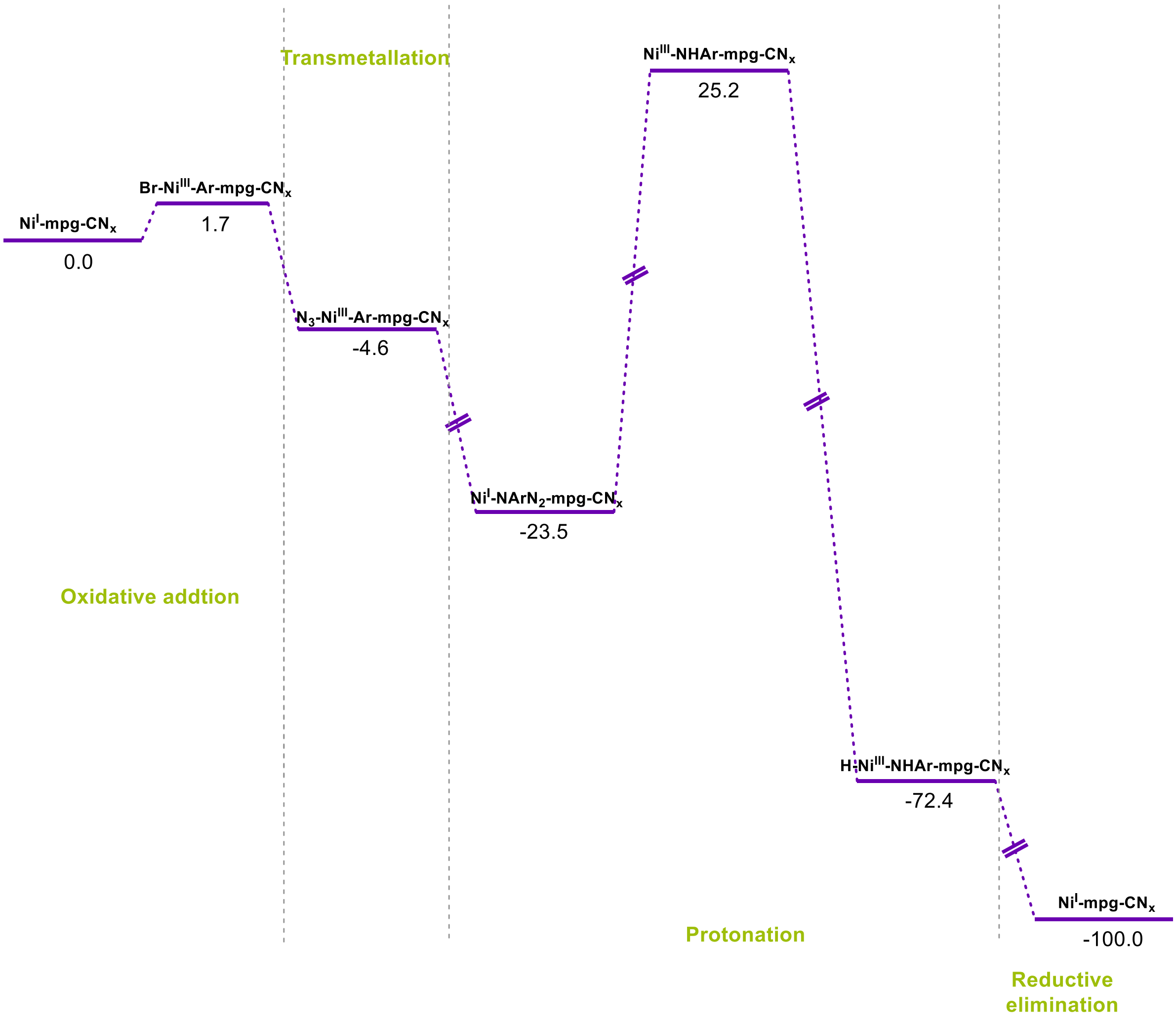


**Figure S9.** Energy profile for the C-N coupling catalytic cycle. Energies are in kcal·mol$^{-1}$. We observed $H_2$ production with the Ni-mpg-CN$_x$ as shown in Table S8, as such, speculated Ni-H intermediate can be involved in the photoreduction of Ar-$N_3$ to aniline. The energy profile supports this as the Ni-H formation is lower in energy than the protonation of aniline N by 4 kcal/mol.

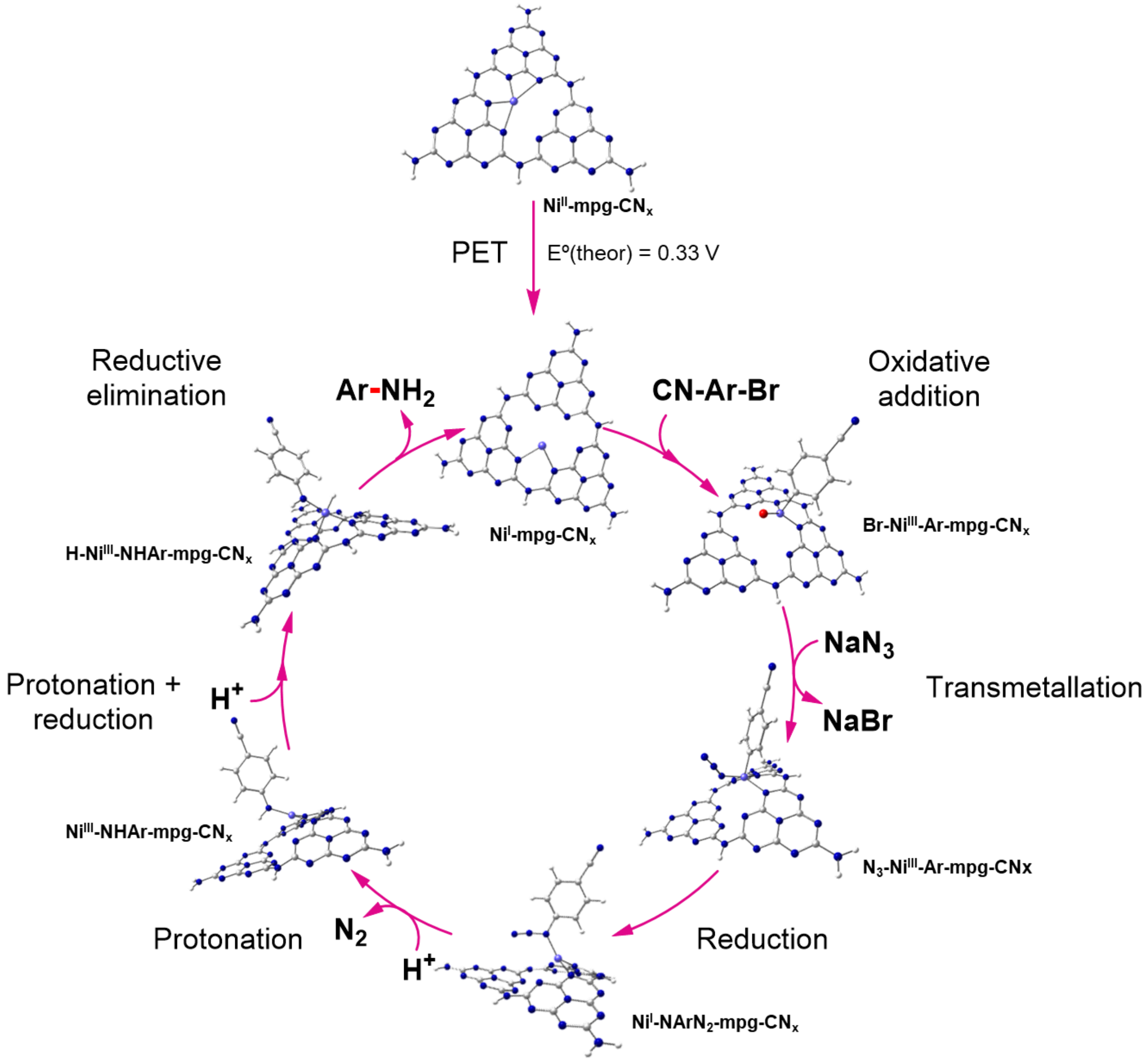


**Figure S10.** Summary of the computed C-N coupling catalytic cycle for Ni-mpg-$CN_x$ with theoretical redox values. Note: values represent the redox potential for the ET values [V *vs* SHE] at 298 K. Energies represent elementary steps and they are given in kcal $mol^{-1}$.

# 6. NMR spectra of purified products

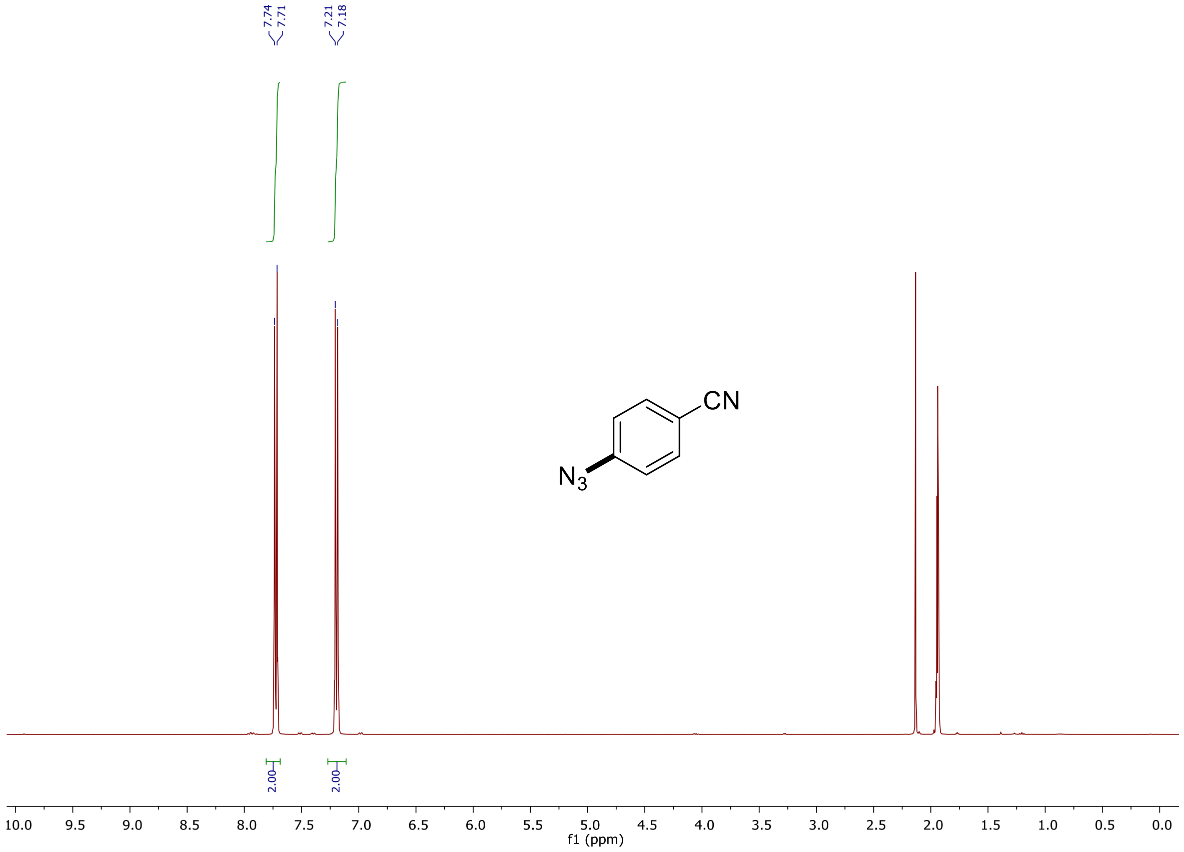


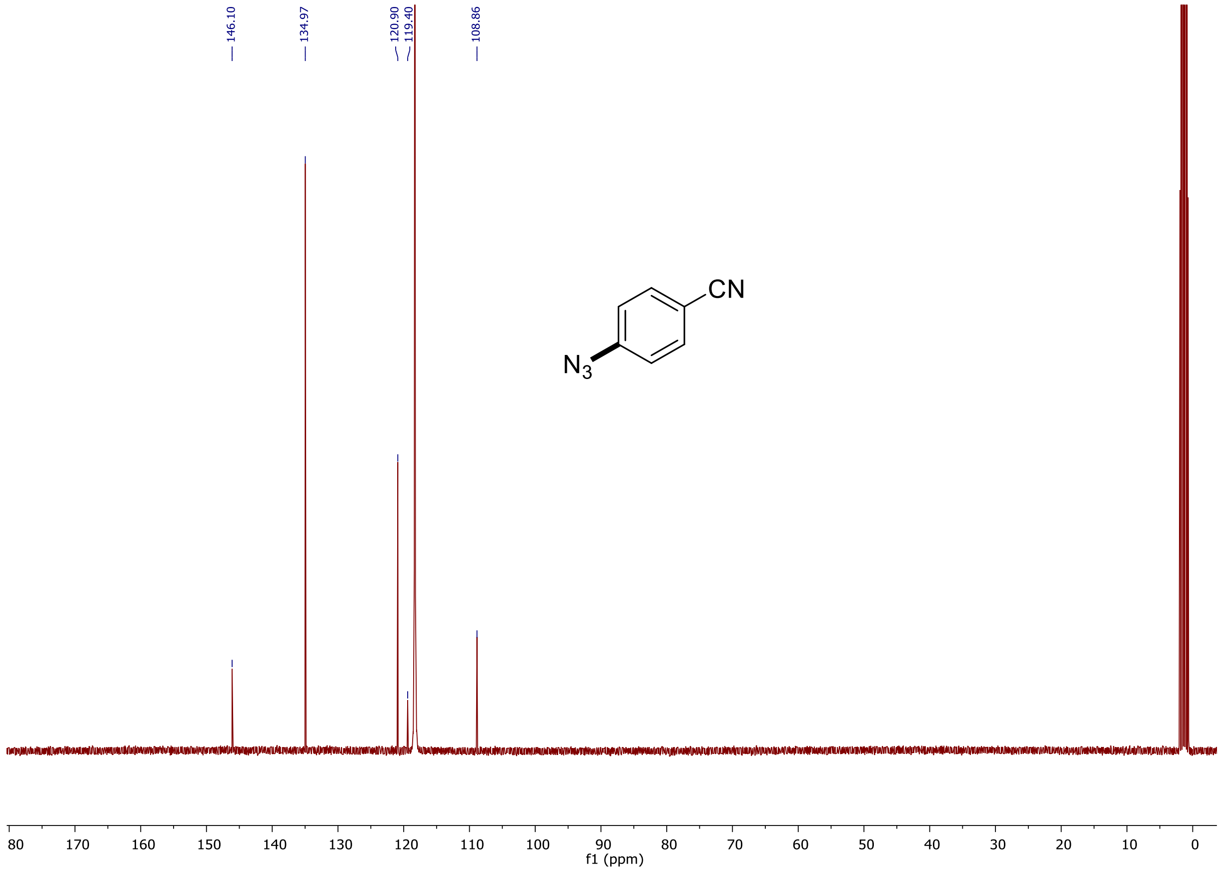

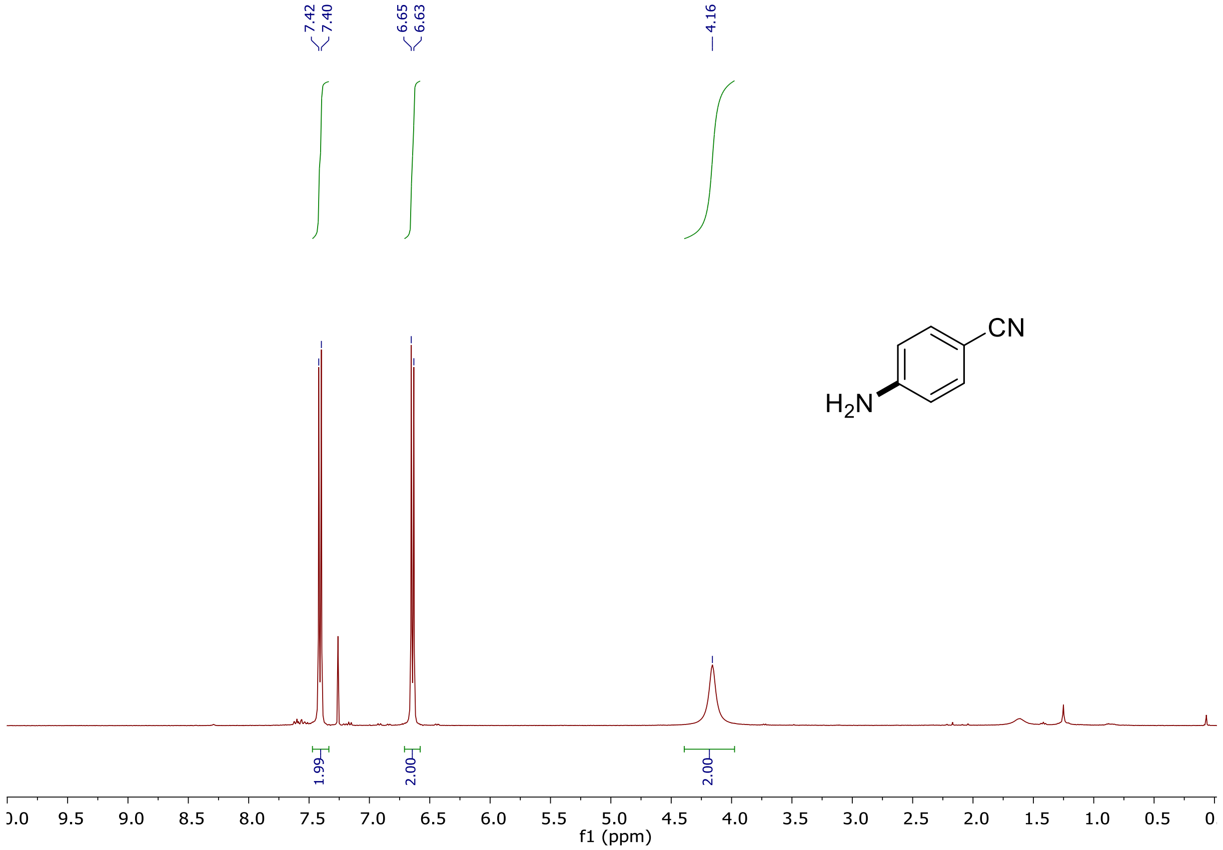
7.42
7.40
6.65
6.63
4.16
CN
H2N
1.99
2.00
2.00
f1 (ppm)

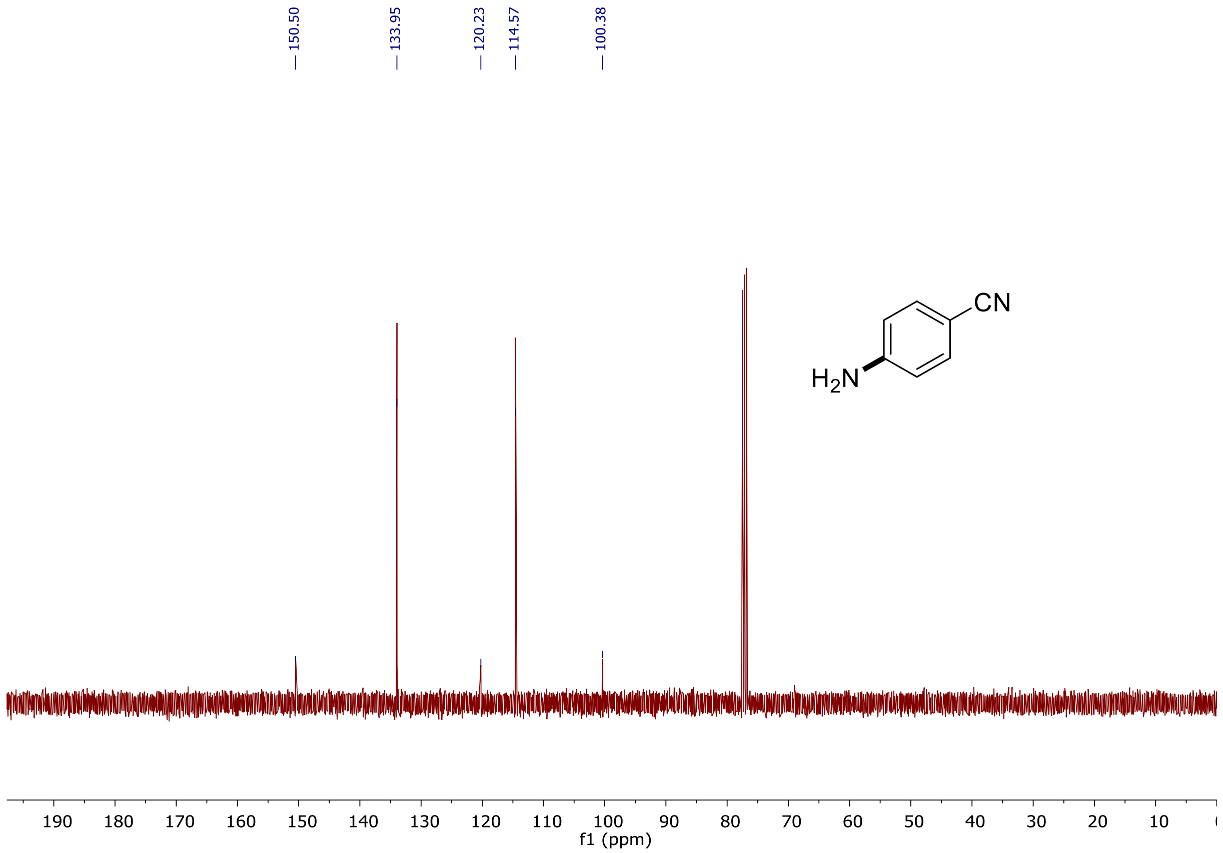
150.50
133.95
120.23
114.57
100.38
CN
H2N
f1 (ppm)

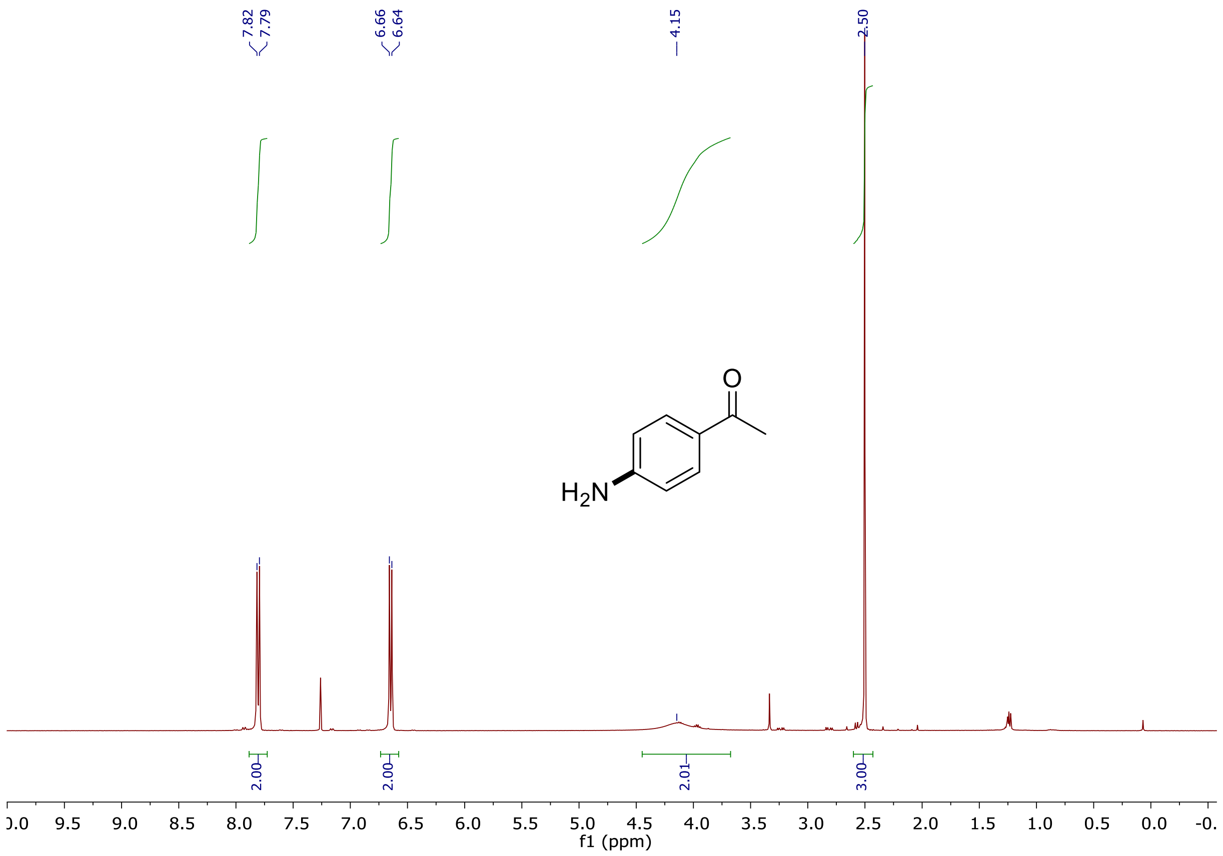
7.82
7.79
6.66
6.64
4.15
2.50
H2N
O
2.00
2.00
2.01
3.00
10.0 9.5 9.0 8.5 8.0 7.5 7.0 6.5 6.0 5.5 5.0 4.5 4.0 3.5 3.0 2.5 2.0 1.5 1.0 0.5 0.0 -0.
f1 (ppm)

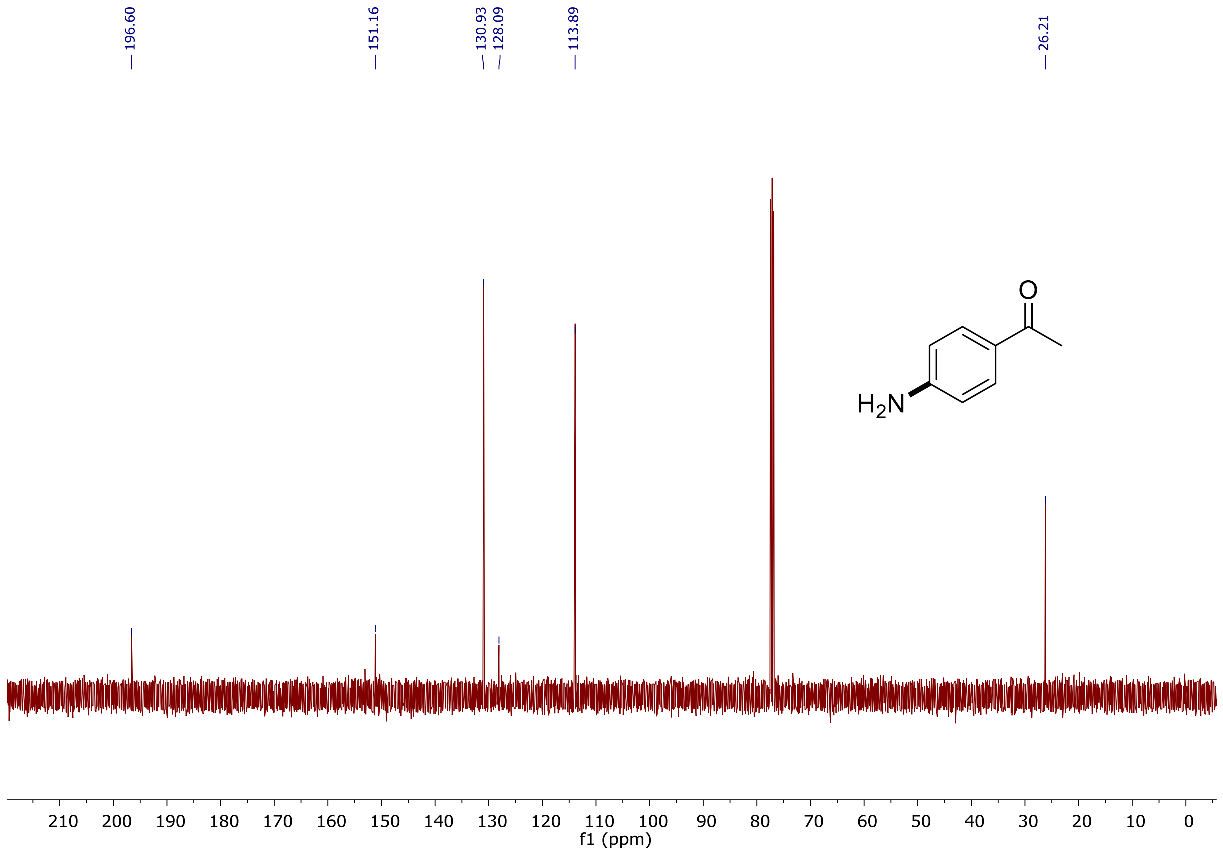
196.60
151.16
130.93
128.09
113.89
26.21
H2N
O
210 200 190 180 170 160 150 140 130 120 110 100 90 80 70 60 50 40 30 20 10 0
f1 (ppm)

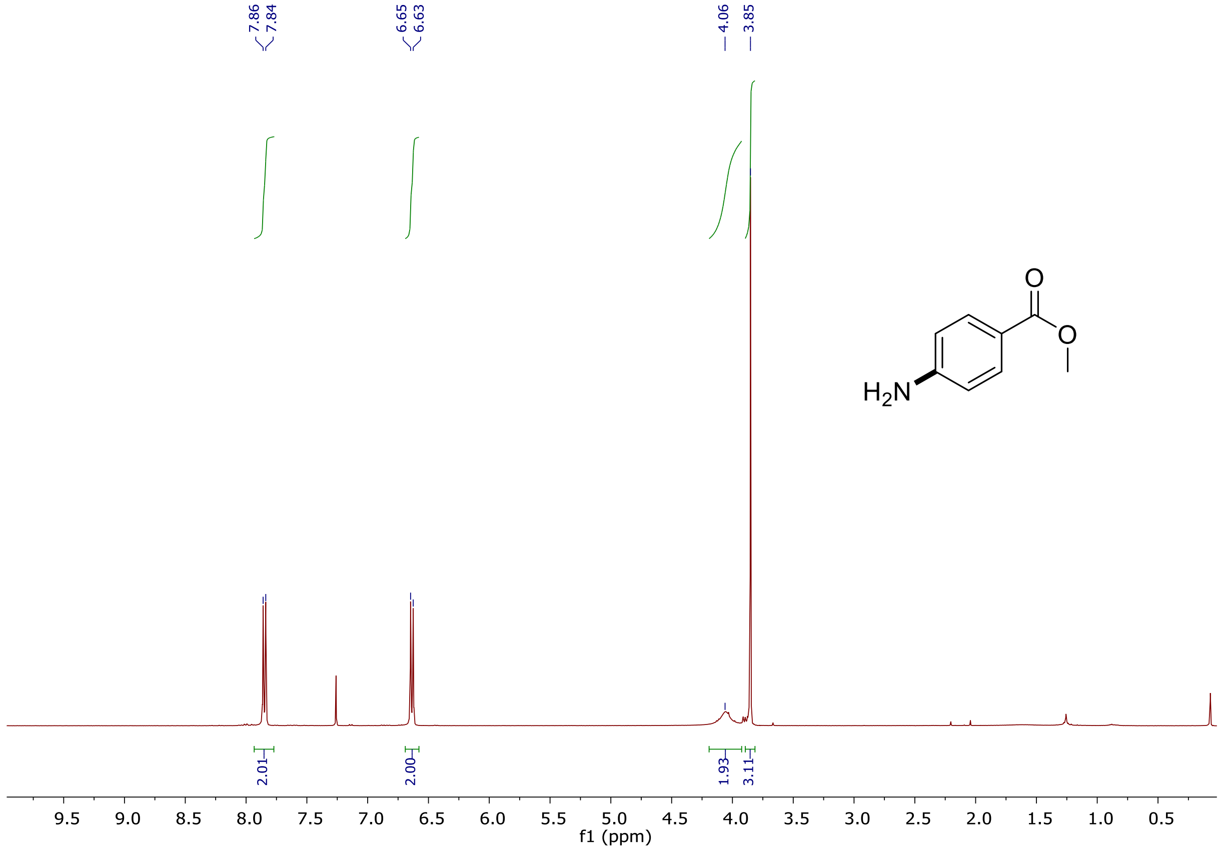

7.86
7.84
6.65
6.63
4.06
3.85
H2N
O
O
2.01
2.00
1.93
3.11
9.5 9.0 8.5 8.0 7.5 7.0 6.5 6.0 5.5 5.0 4.5 4.0 3.5 3.0 2.5 2.0 1.5 1.0 0.5
f1 (ppm)


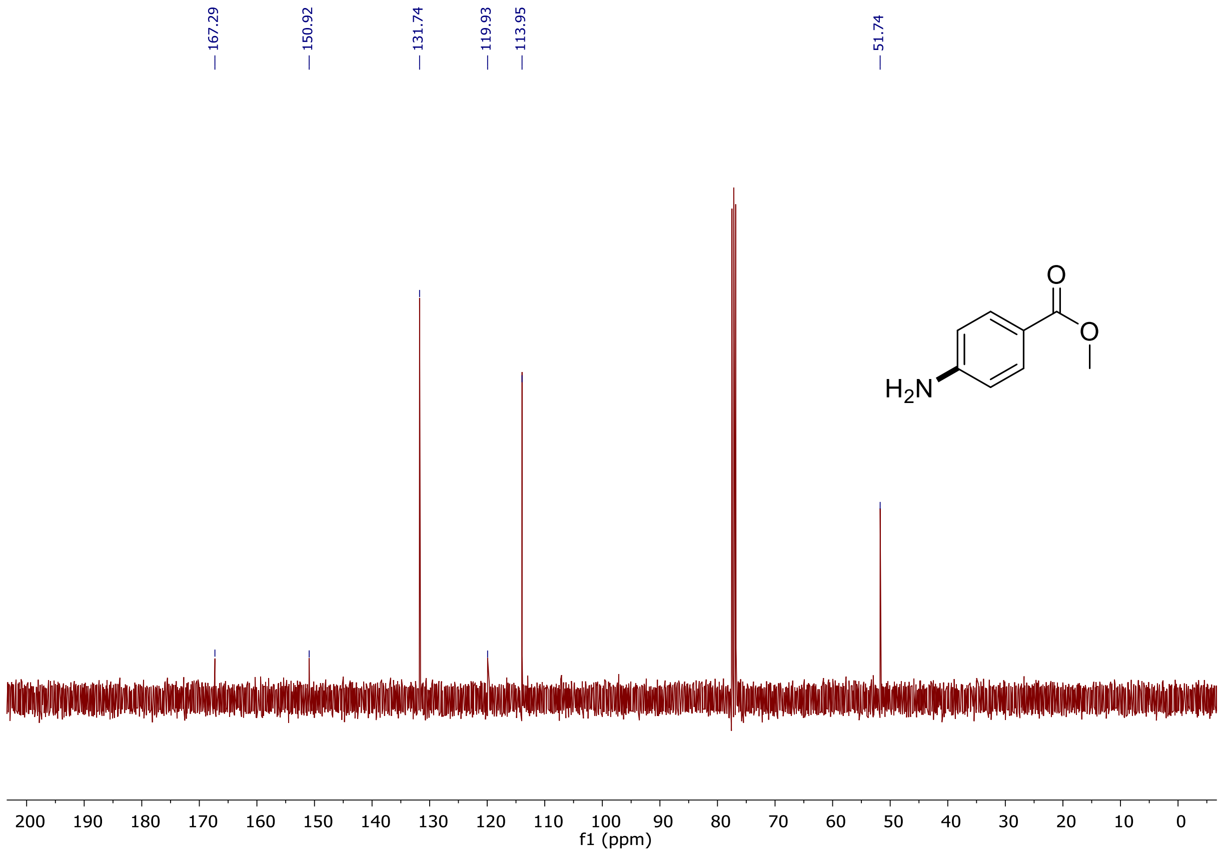

167.29
150.92
131.74
119.93
113.95
51.74
H2N
O
O
200 190 180 170 160 150 140 130 120 110 100 90 80 70 60 50 40 30 20 10 0
f1 (ppm)

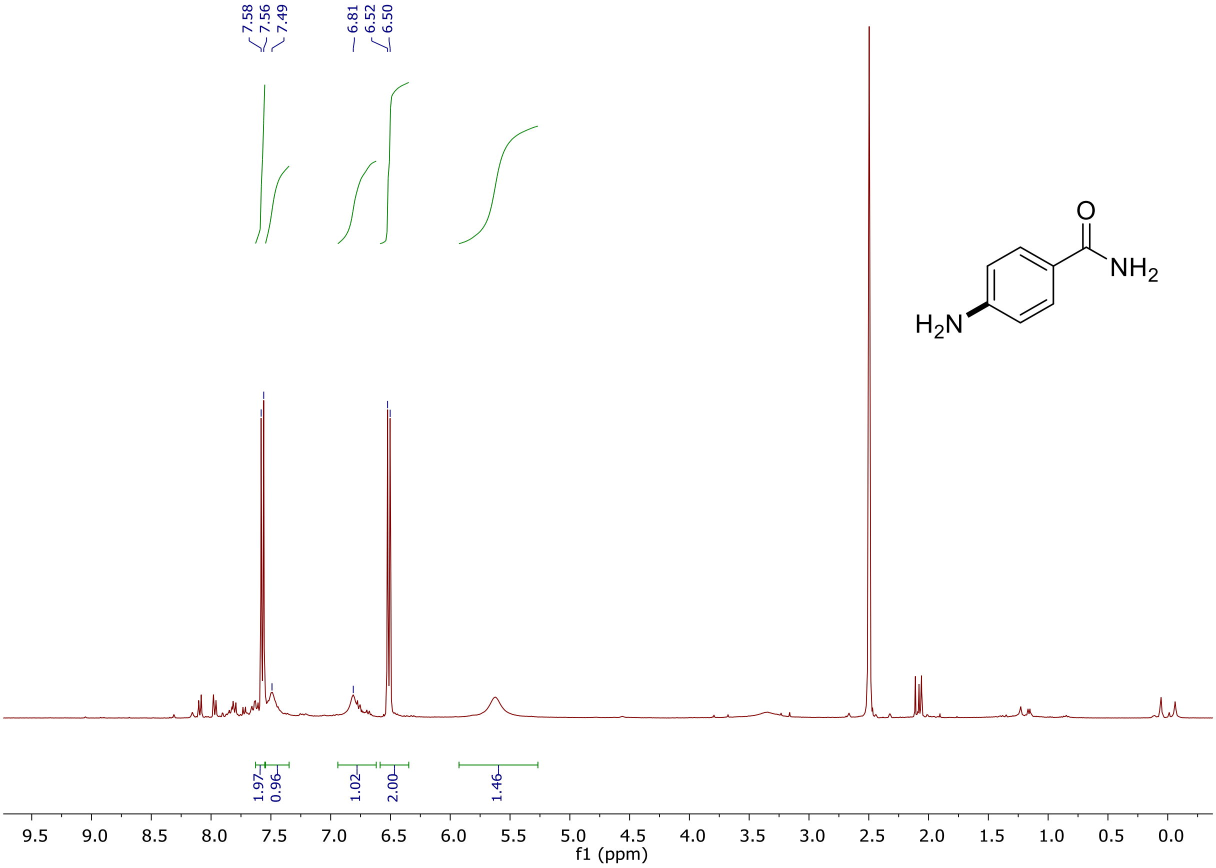
7.58
7.56
7.49
6.81
6.52
6.50
1.97
0.96
1.02
2.00
1.46
9.5 9.0 8.5 8.0 7.5 7.0 6.5 6.0 5.5 5.0 4.5 4.0 3.5 3.0 2.5 2.0 1.5 1.0 0.5 0.0
f1 (ppm)
O
NH2
H2N

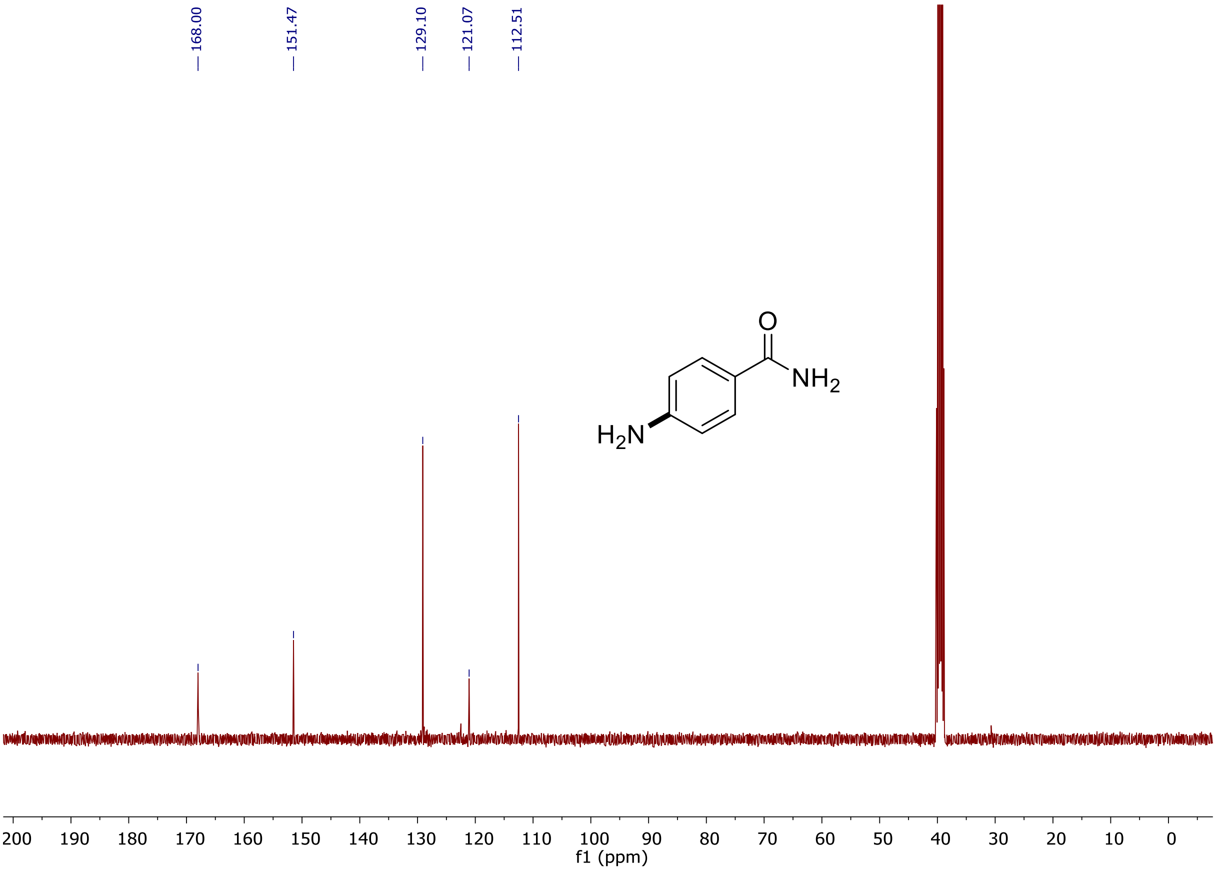
168.00
151.47
129.10
121.07
112.51
O
NH2
H2N
200 190 180 170 160 150 140 130 120 110 100 90 80 70 60 50 40 30 20 10 0
f1 (ppm)

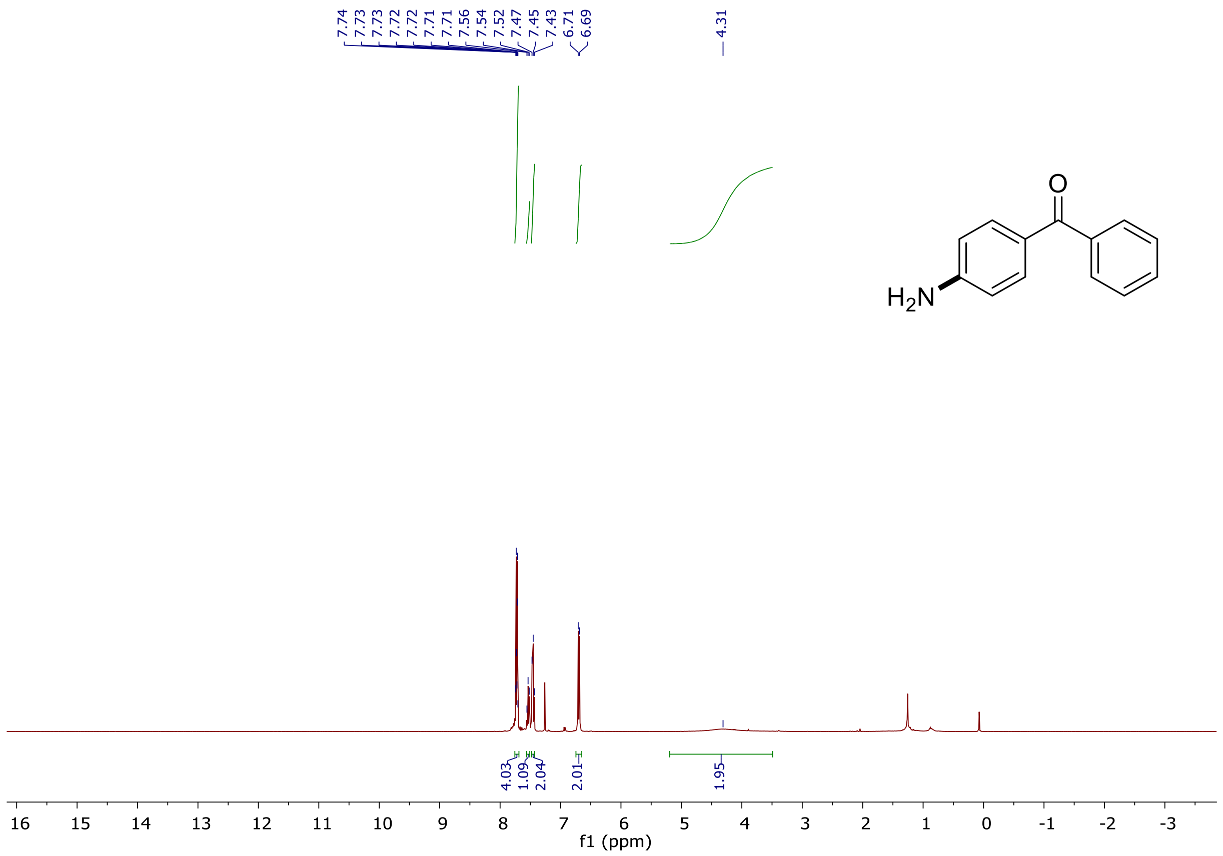
7.74
7.73
7.73
7.72
7.72
7.71
7.71
7.56
7.54
7.52
7.47
7.45
7.43
6.71
6.69
4.31
4.03
1.09
2.04
2.01
1.95
f1 (ppm)
H2N
O

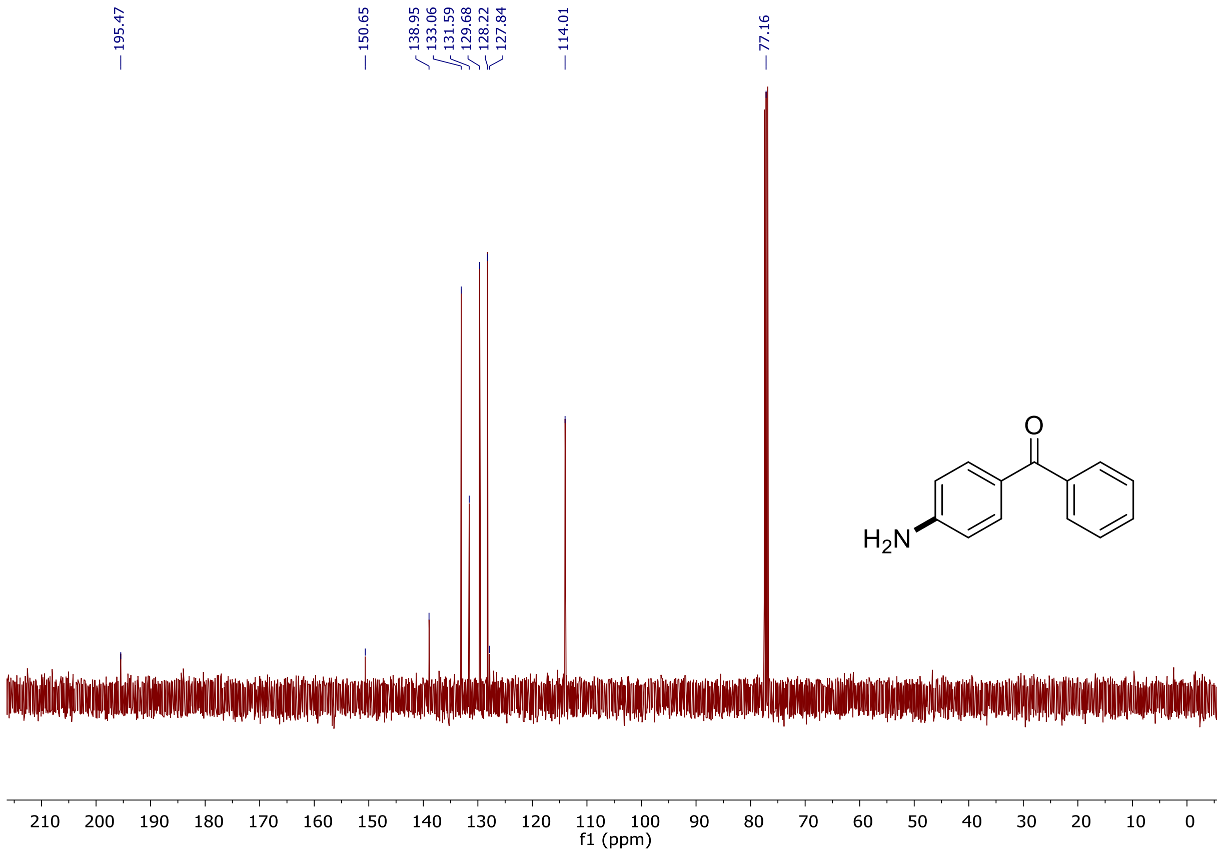
195.47
150.65
138.95
133.06
131.59
129.68
128.22
127.84
114.01
77.16
f1 (ppm)
H2N
O

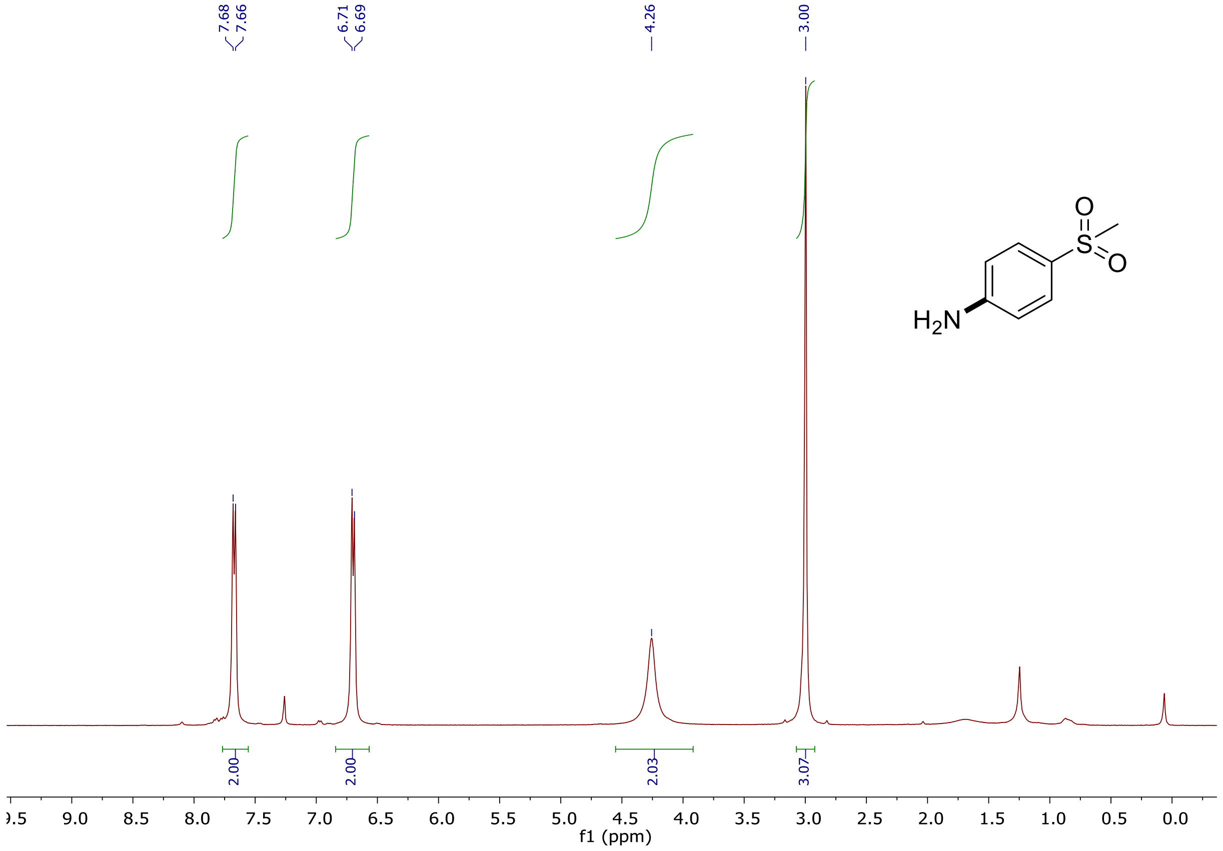
7.68
7.66
6.71
6.69
4.26
3.00
H2N
S
O
O
2.00
2.00
2.03
3.07
9.5
9.0
8.5
8.0
7.5
7.0
6.5
6.0
5.5
5.0
4.5
4.0
3.5
3.0
2.5
2.0
1.5
1.0
0.5
0.0
f1 (ppm)

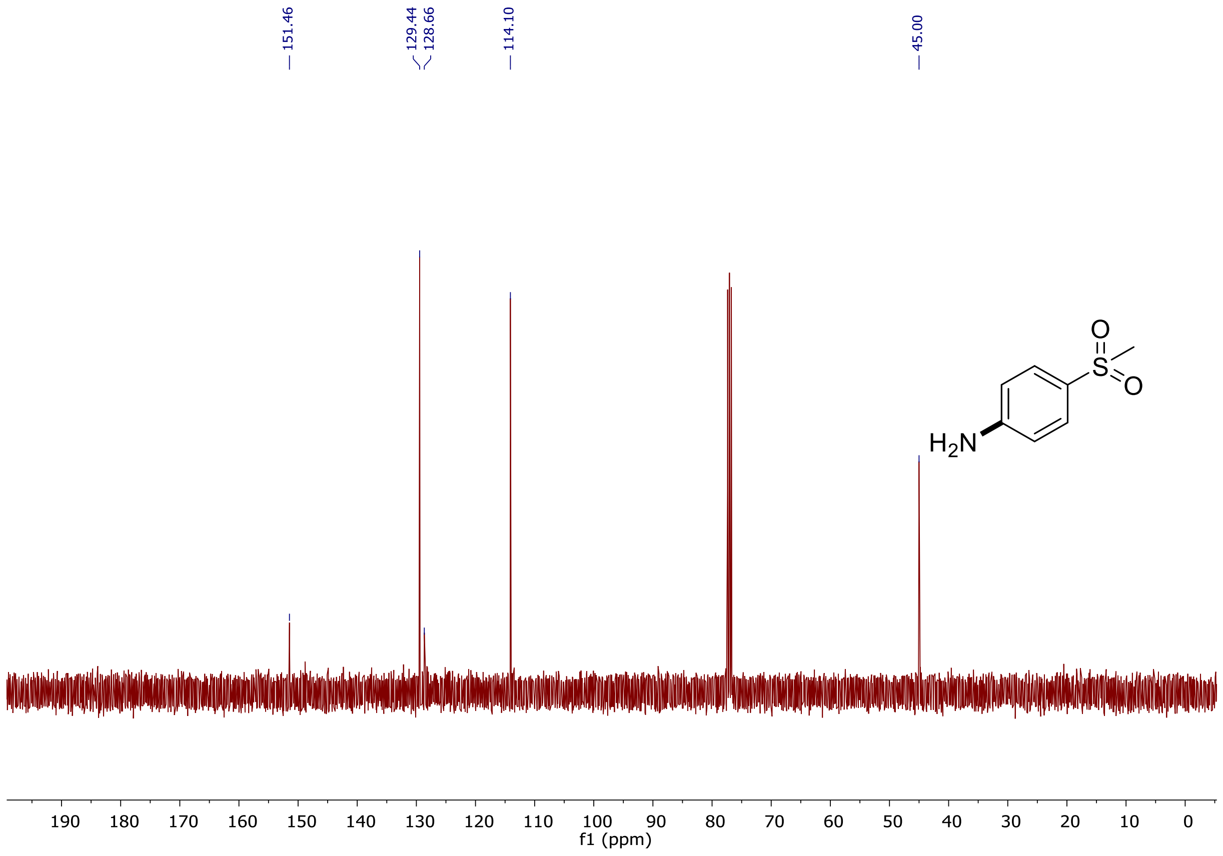
151.46
129.44
128.66
114.10
45.00
H2N
S
O
O
190
180
170
160
150
140
130
120
110
100
90
80
70
60
50
40
30
20
10
0
f1 (ppm)

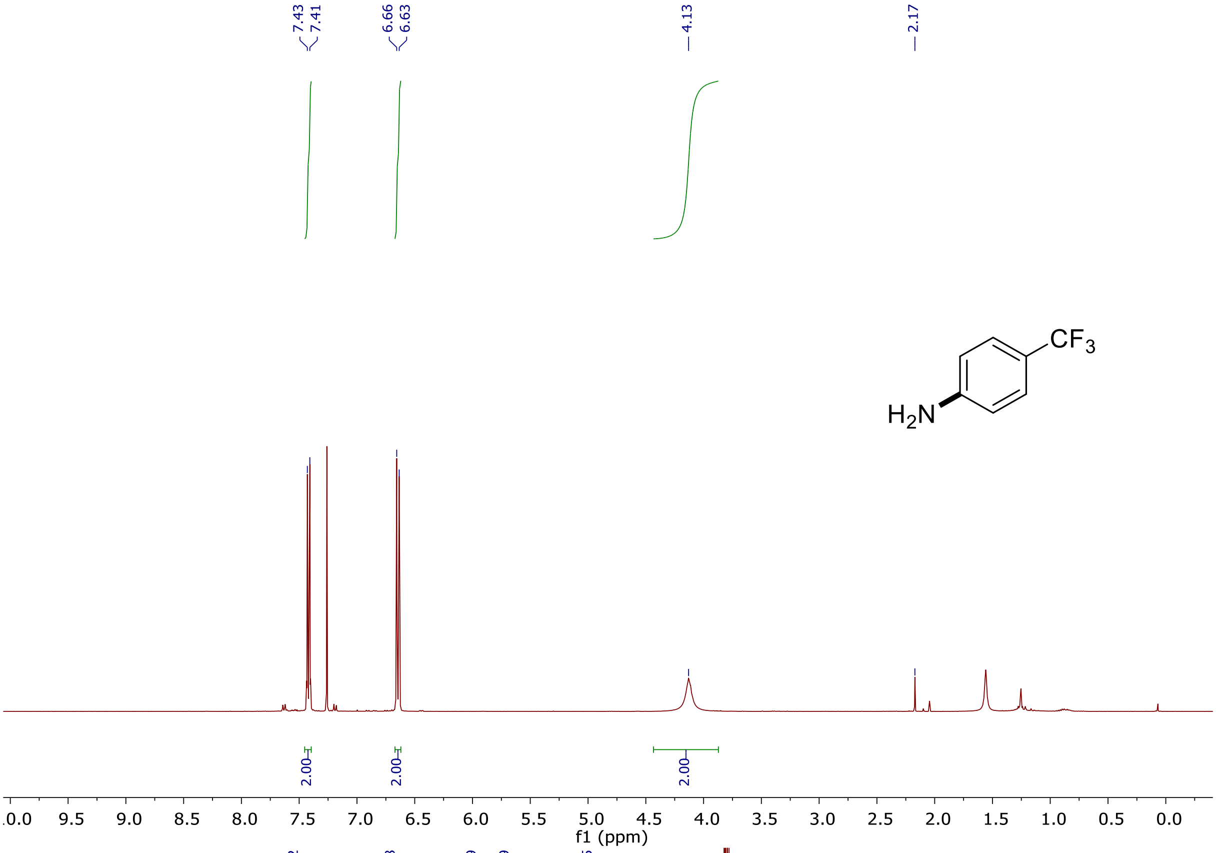
7.43
7.41
6.66
6.63
4.13
2.17
CF3
H2N
2.00
2.00
2.00
f1 (ppm)

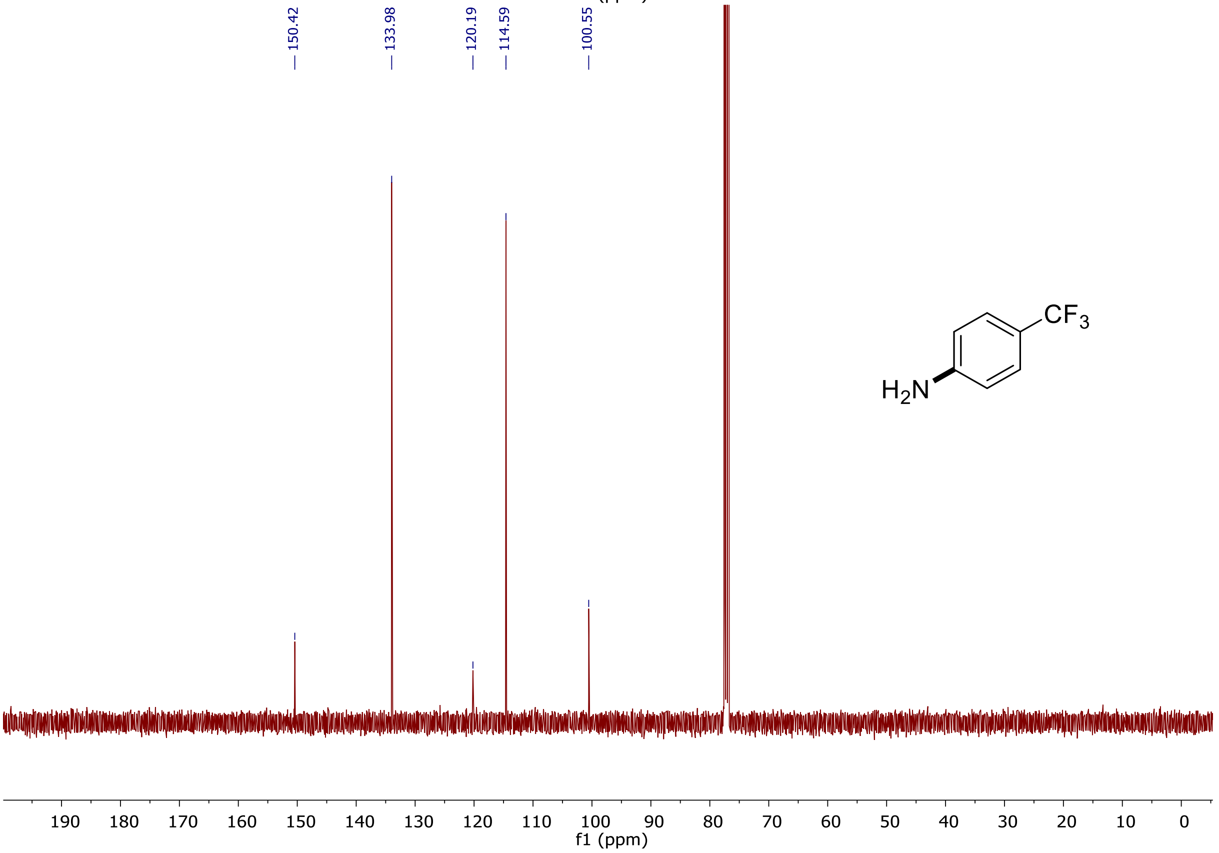
150.42
133.98
120.19
114.59
100.55
CF3
H2N
f1 (ppm)

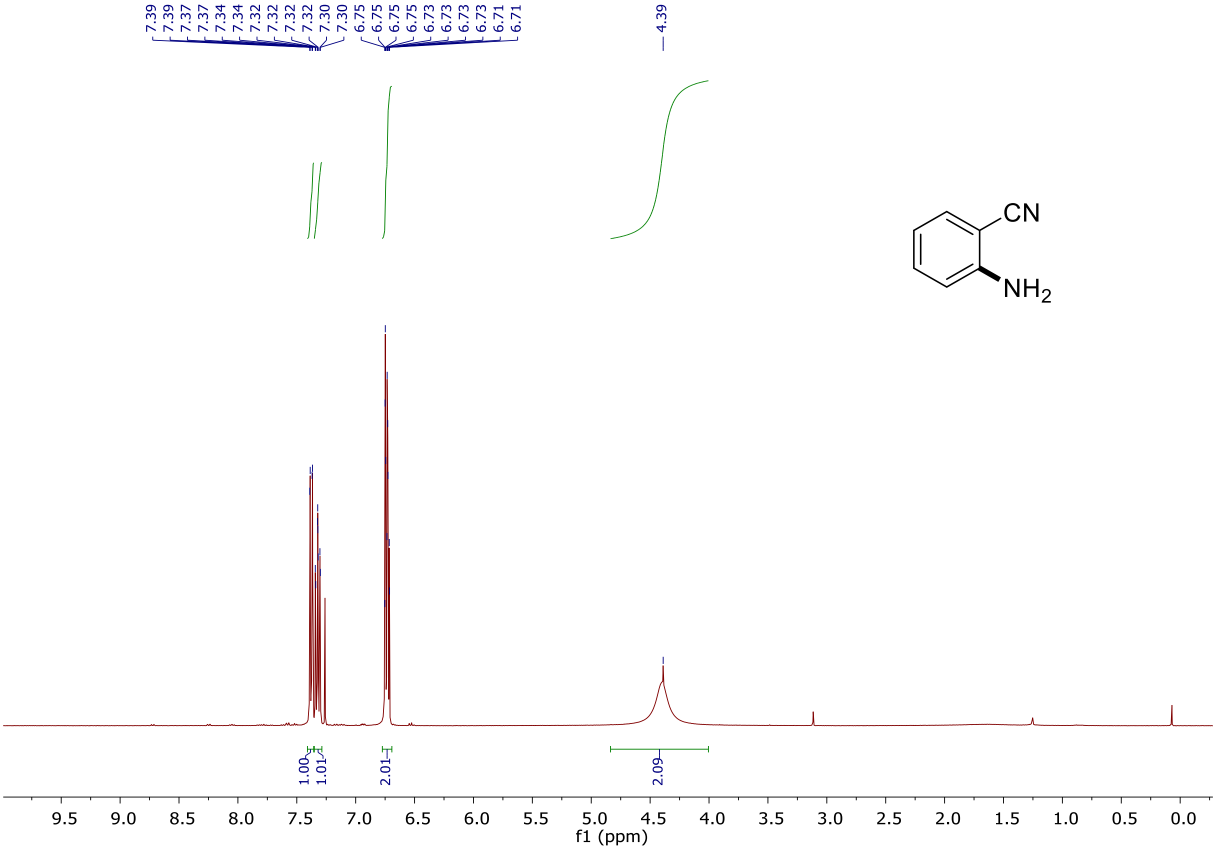
7.39
7.39
7.37
7.37
7.34
7.34
7.32
7.32
7.32
7.32
7.30
7.30
6.75
6.75
6.75
6.75
6.73
6.73
6.73
6.73
6.71
6.71
4.39
CN
NH2
1.00
1.01
2.01
2.09
9.5 9.0 8.5 8.0 7.5 7.0 6.5 6.0 5.5 5.0 4.5 4.0 3.5 3.0 2.5 2.0 1.5 1.0 0.5 0.0
f1 (ppm)

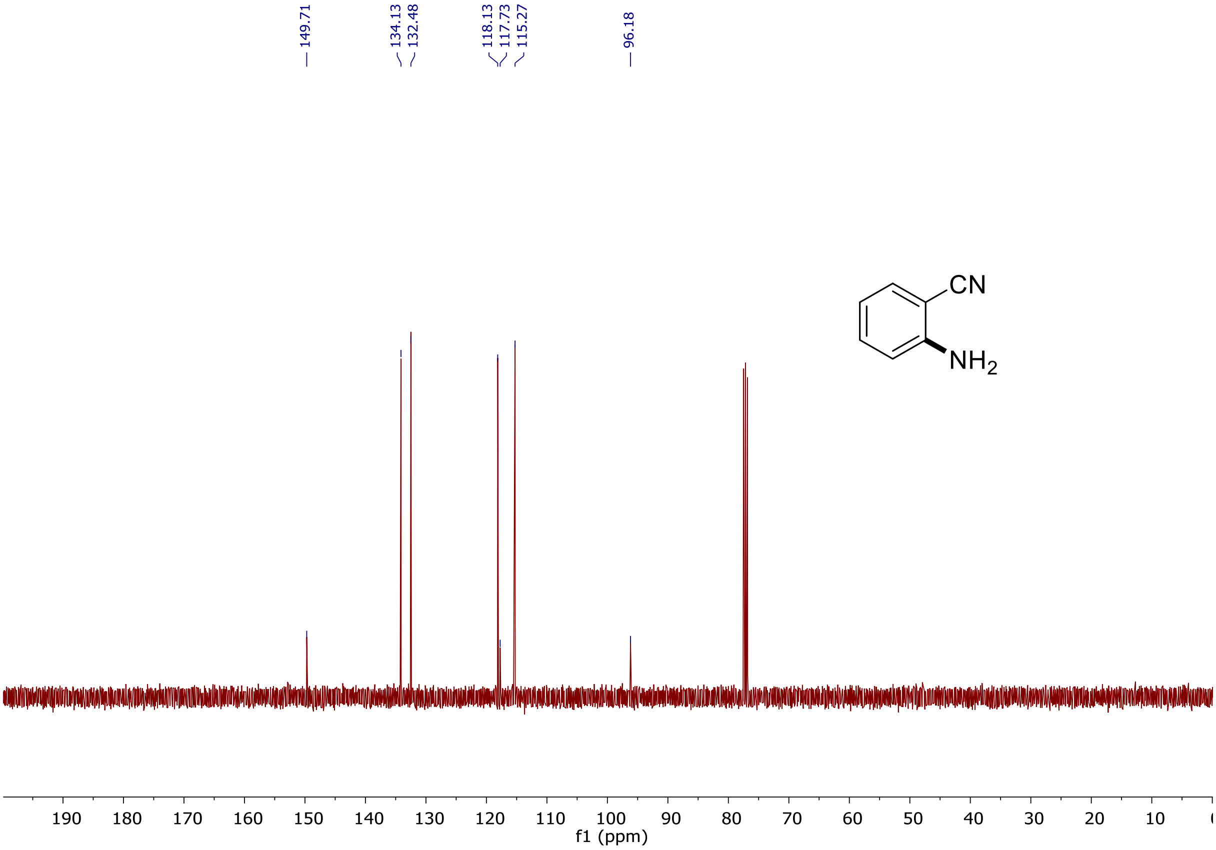
149.71
134.13
132.48
118.13
117.73
115.27
96.18
CN
NH2
190 180 170 160 150 140 130 120 110 100 90 80 70 60 50 40 30 20 10
f1 (ppm)

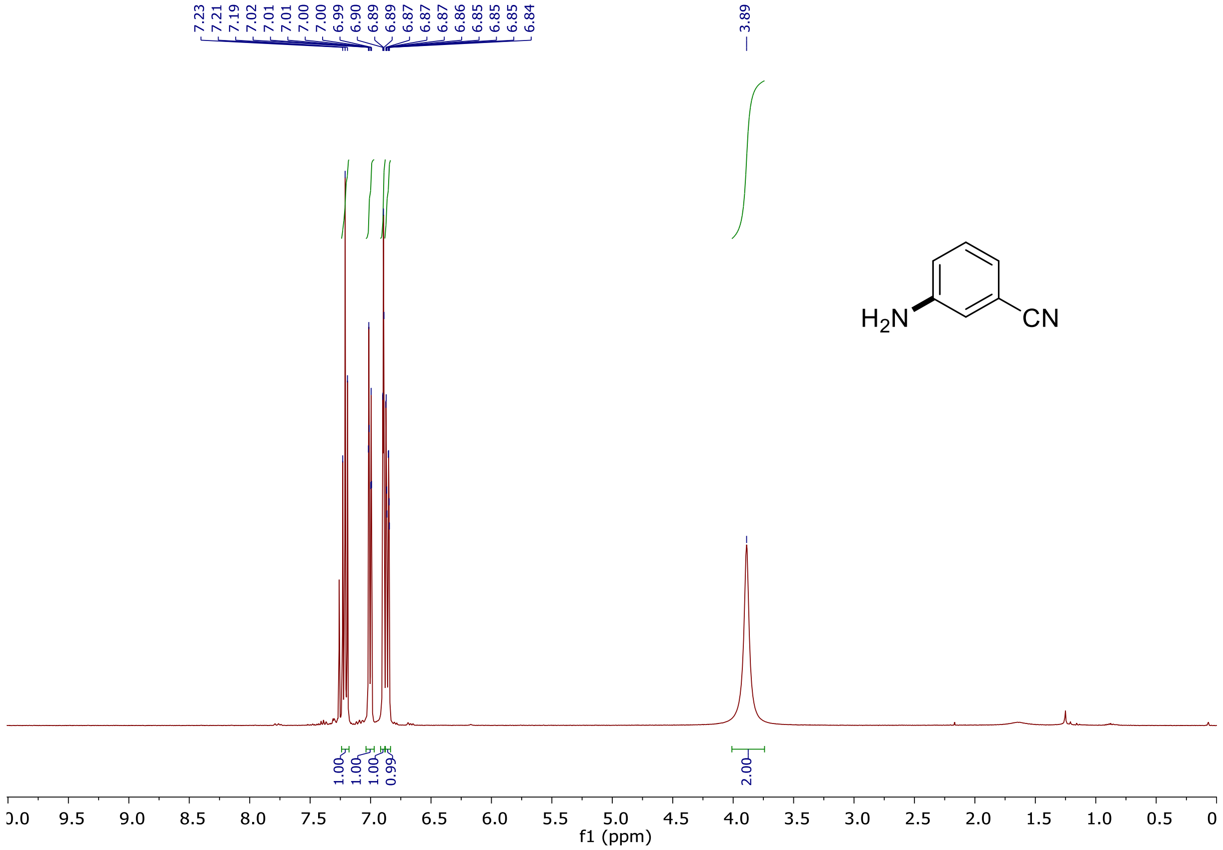
7.23
7.21
7.19
7.02
7.01
7.01
7.00
6.99
6.90
6.89
6.89
6.87
6.87
6.86
6.85
6.85
6.84
3.89
H2N
CN
1.00
1.00
1.00
0.99
2.00
f1 (ppm)

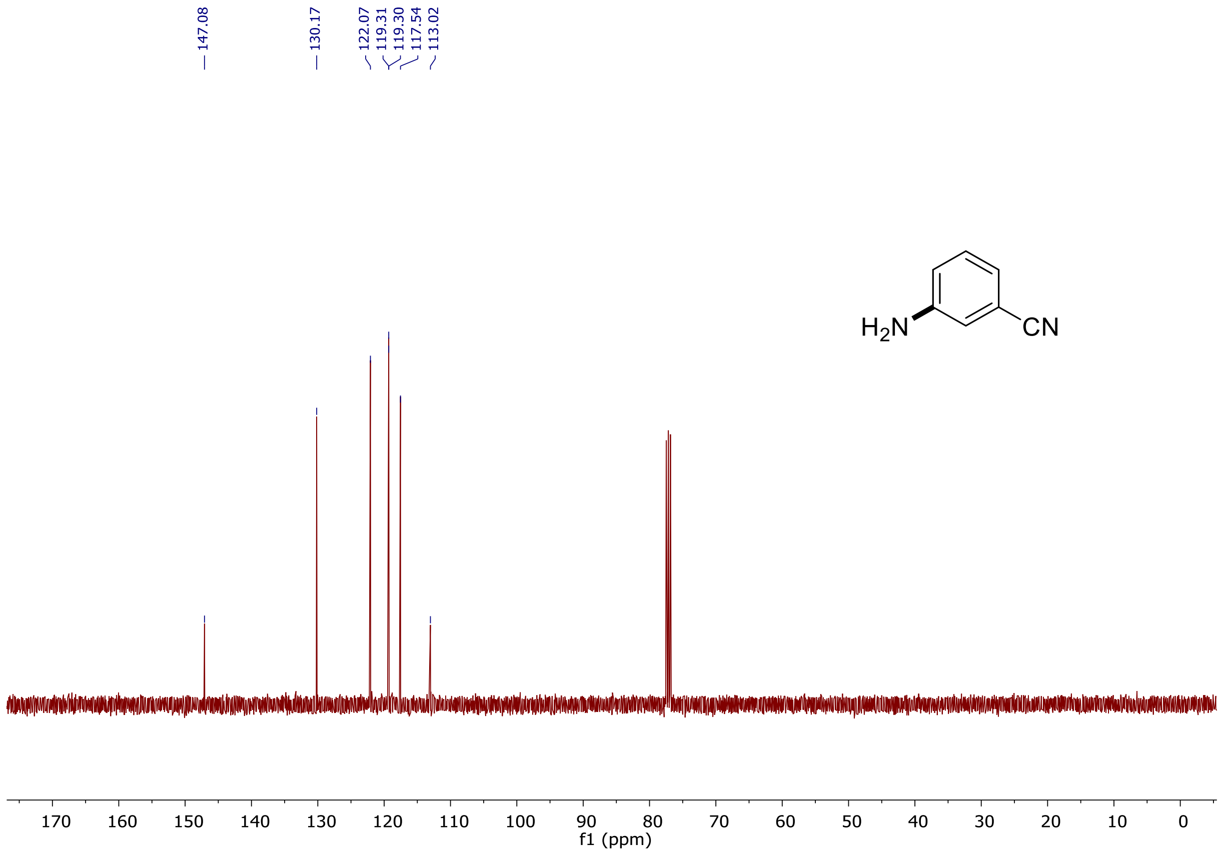
147.08
130.17
122.07
119.31
119.30
117.54
113.02
H2N
CN
f1 (ppm)

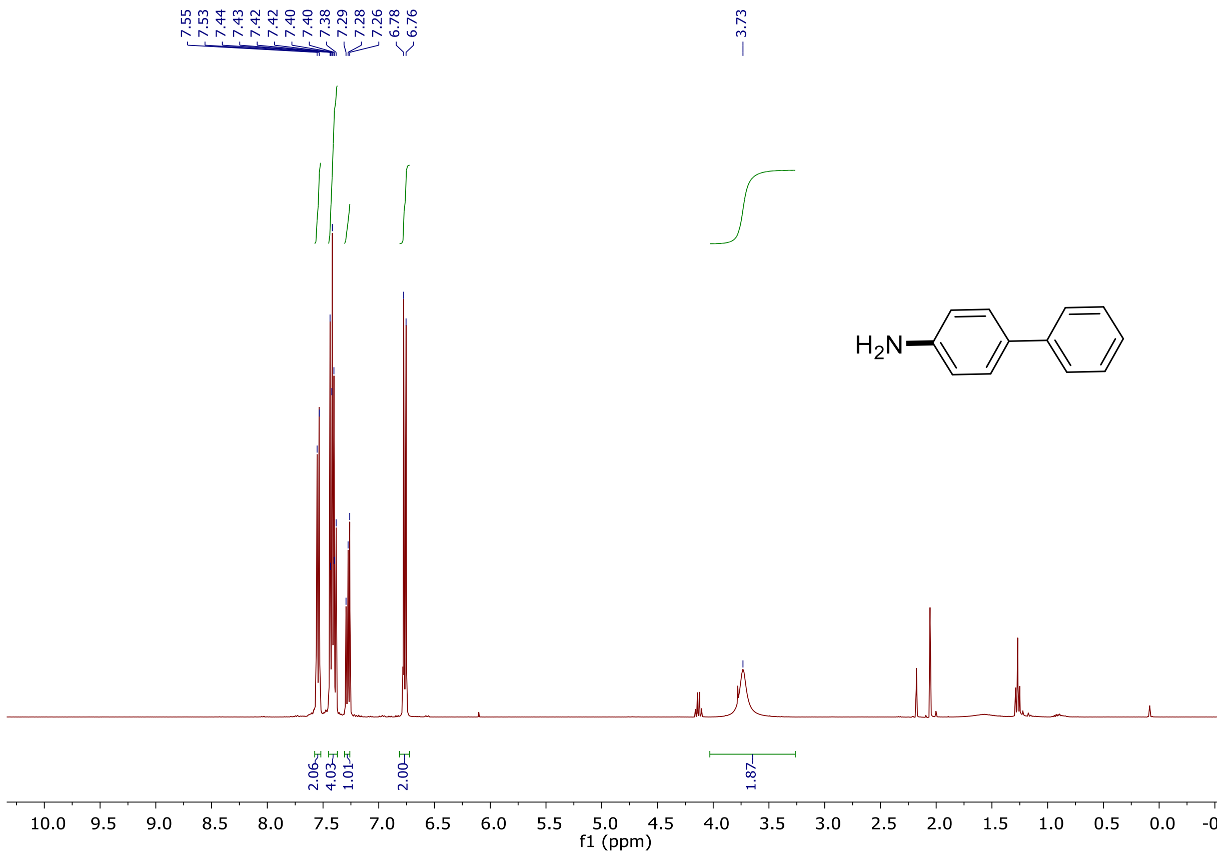
7.55
7.53
7.44
7.43
7.42
7.42
7.40
7.40
7.38
7.29
7.28
7.26
6.78
6.76
3.73
H2N
2.06
4.03
1.01
2.00
1.87
10.0 9.5 9.0 8.5 8.0 7.5 7.0 6.5 6.0 5.5 5.0 4.5 4.0 3.5 3.0 2.5 2.0 1.5 1.0 0.5 0.0
f1 (ppm)

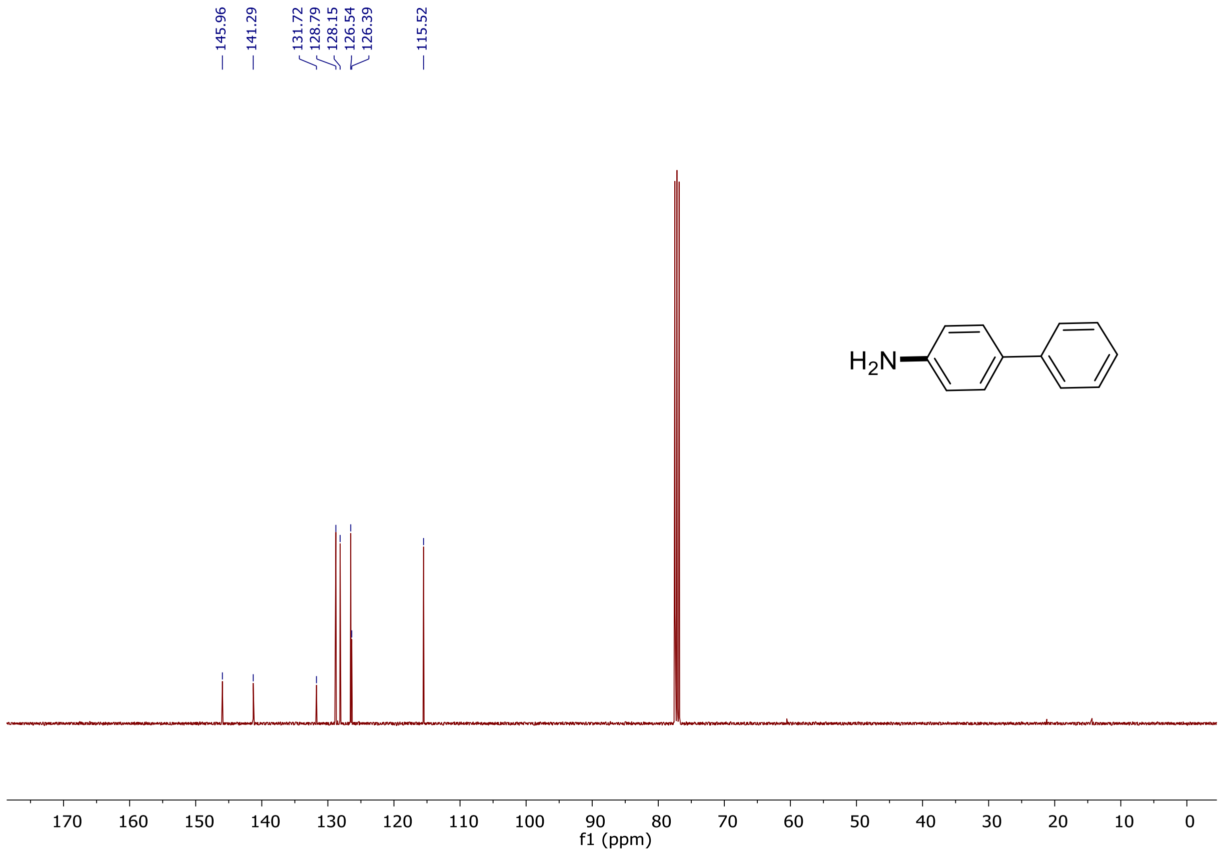
145.96
141.29
131.72
128.79
128.15
126.54
126.39
115.52
H2N
170 160 150 140 130 120 110 100 90 80 70 60 50 40 30 20 10 0
f1 (ppm)

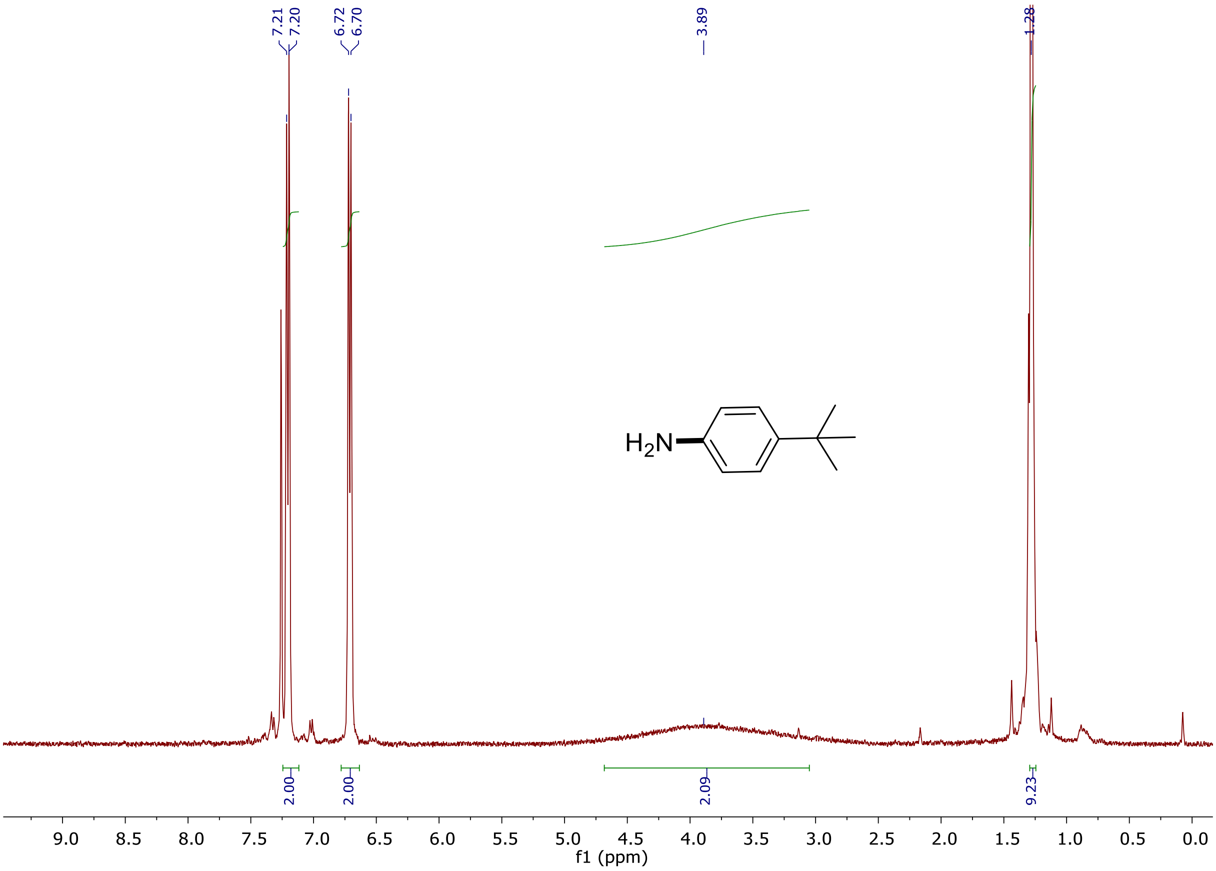
7.21
7.20
6.72
6.70
3.89
1.28
H2N
2.00
2.00
2.09
9.23
9.0 8.5 8.0 7.5 7.0 6.5 6.0 5.5 5.0 4.5 4.0 3.5 3.0 2.5 2.0 1.5 1.0 0.5 0.0
f1 (ppm)

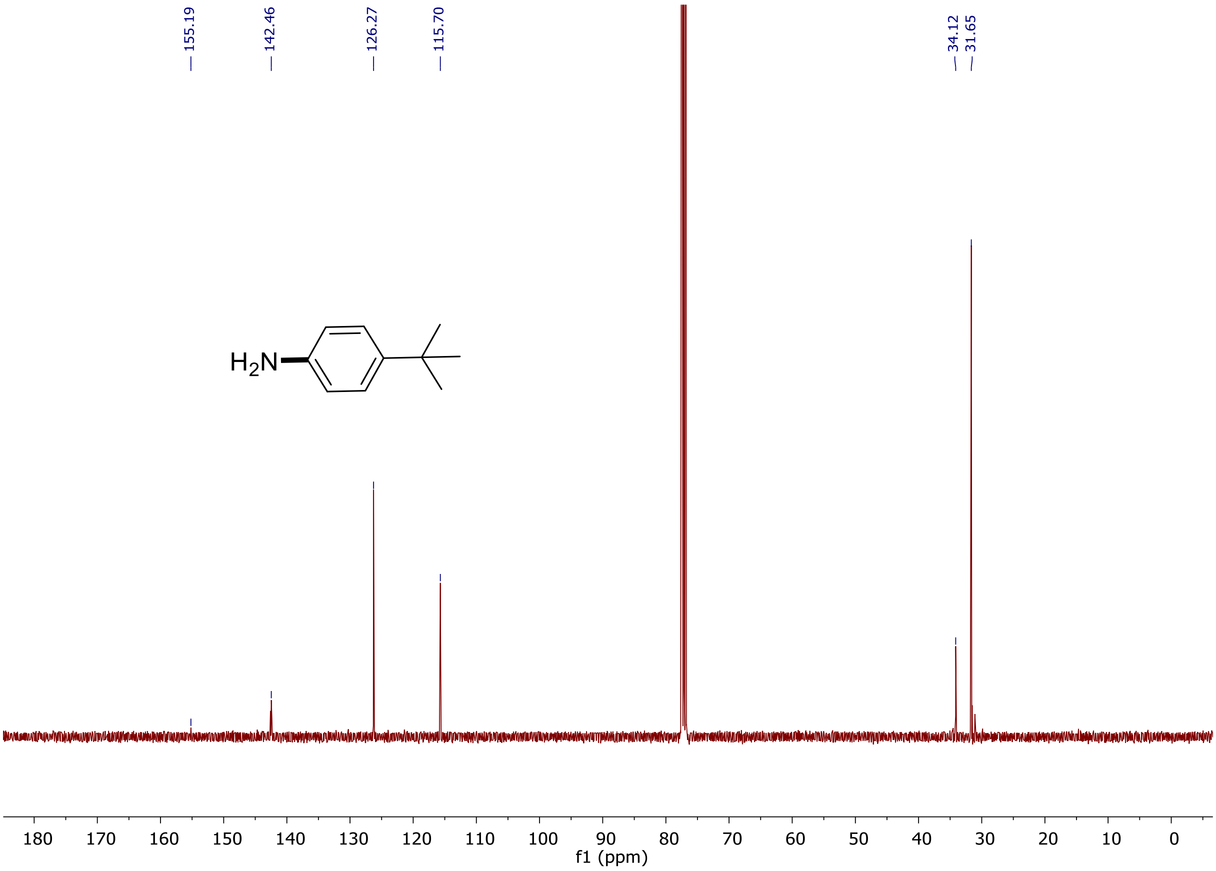
155.19
142.46
126.27
115.70
34.12
31.65
H2N
180 170 160 150 140 130 120 110 100 90 80 70 60 50 40 30 20 10 0
f1 (ppm)

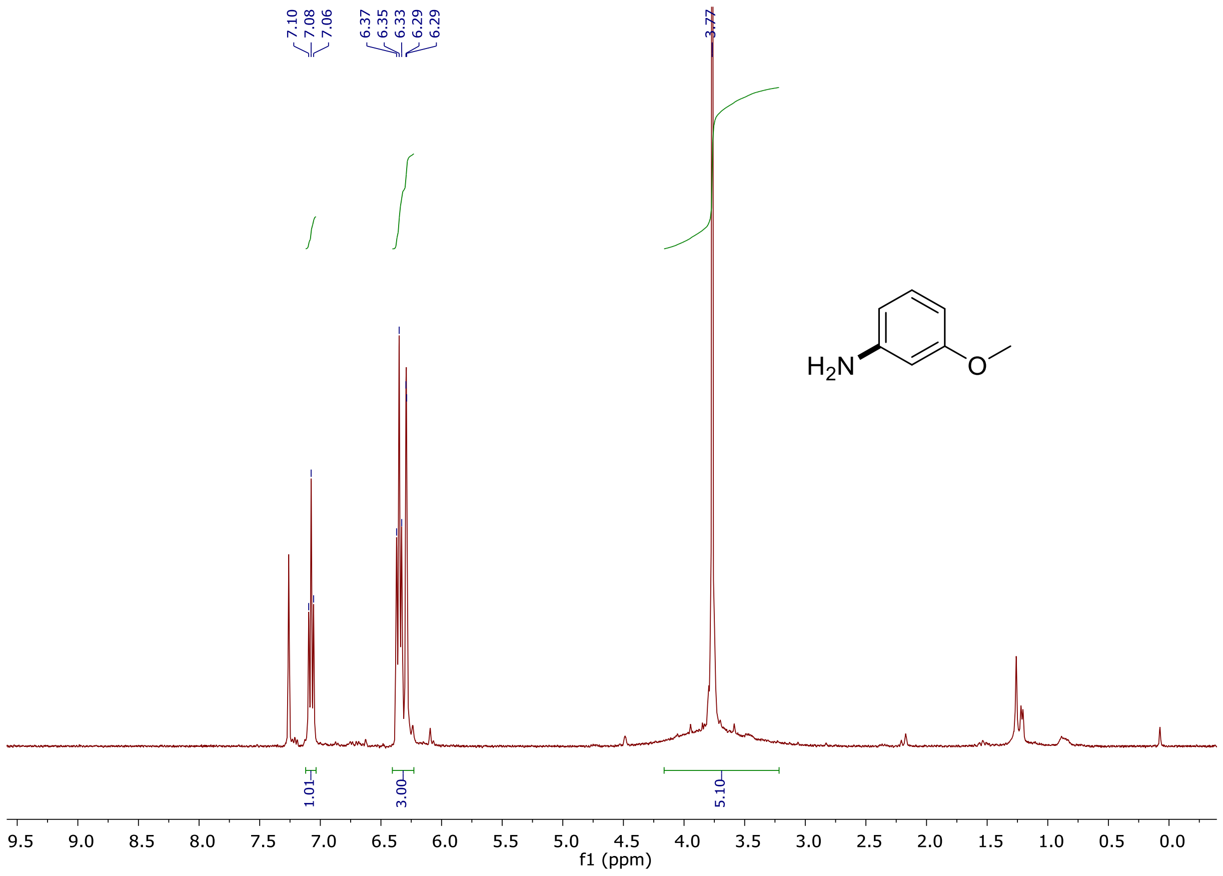
7.10
7.08
7.06
6.37
6.35
6.33
6.29
6.29
3.77
H2N
O
1.01
3.00
5.10
9.5 9.0 8.5 8.0 7.5 7.0 6.5 6.0 5.5 5.0 4.5 4.0 3.5 3.0 2.5 2.0 1.5 1.0 0.5 0.0
f1 (ppm)

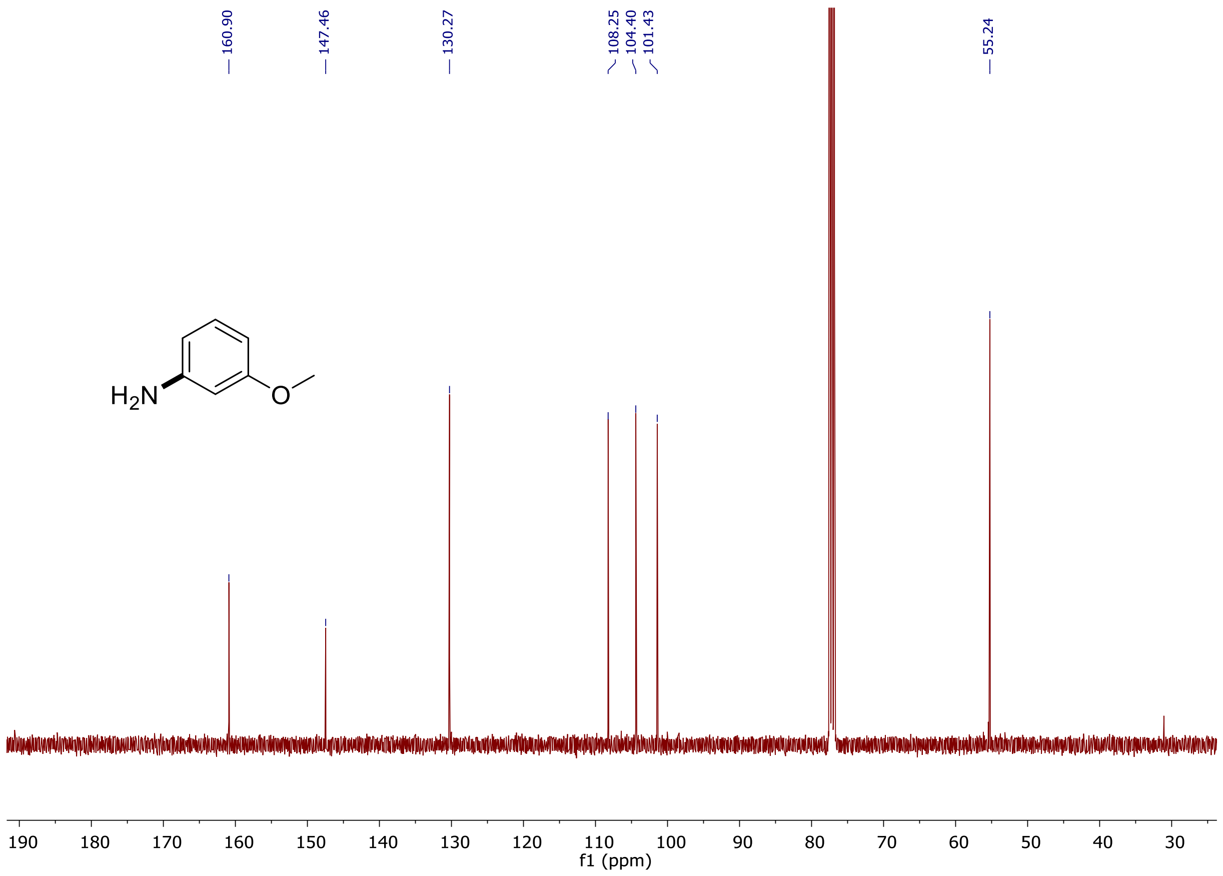
160.90
147.46
130.27
108.25
104.40
101.43
55.24
H2N
O
190 180 170 160 150 140 130 120 110 100 90 80 70 60 50 40 30
f1 (ppm)

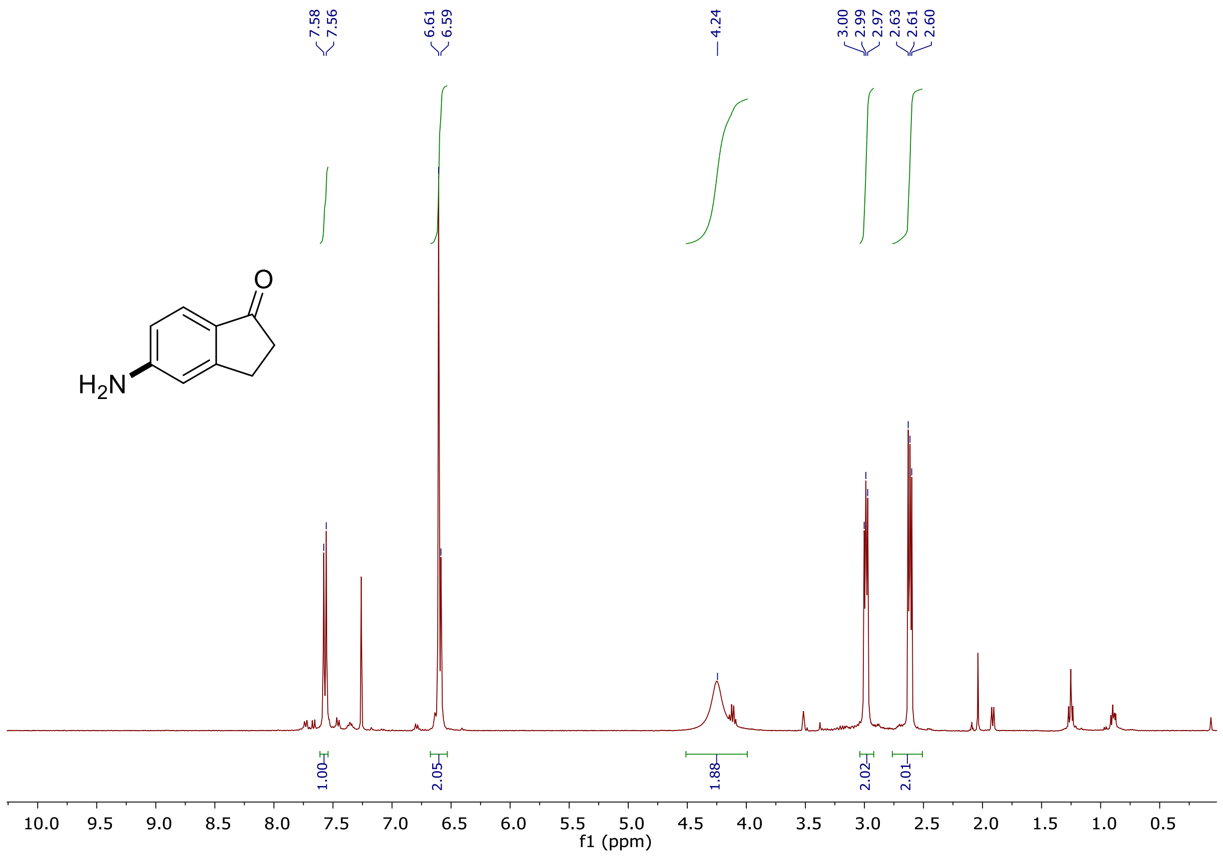
7.58
7.56
6.61
6.59
4.24
3.00
2.99
2.97
2.63
2.61
2.60
H2N
O
1.00
2.05
1.88
2.02
2.01
10.0 9.5 9.0 8.5 8.0 7.5 7.0 6.5 6.0 5.5 5.0 4.5 4.0 3.5 3.0 2.5 2.0 1.5 1.0 0.5
f1 (ppm)

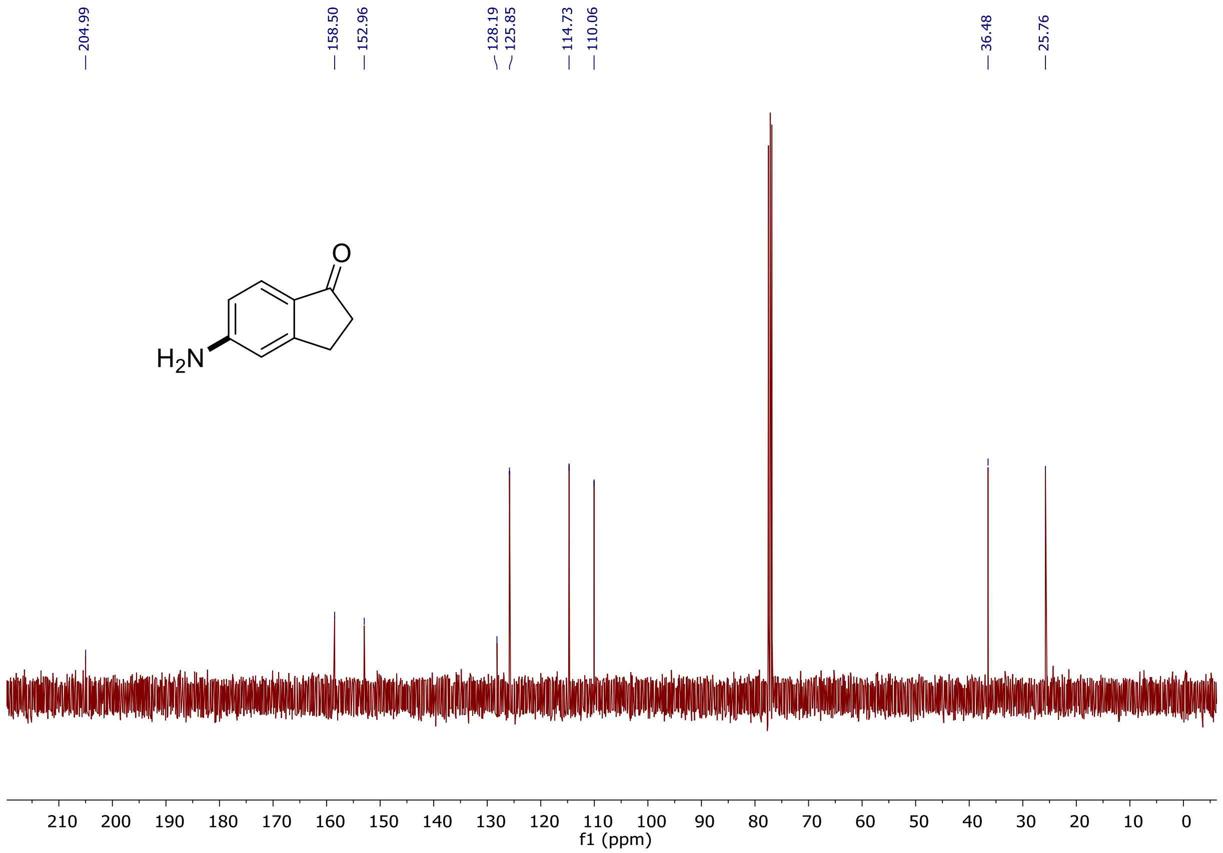
204.99
158.50
152.96
128.19
125.85
114.73
110.06
36.48
25.76
H2N
O
210 200 190 180 170 160 150 140 130 120 110 100 90 80 70 60 50 40 30 20 10 0
f1 (ppm)

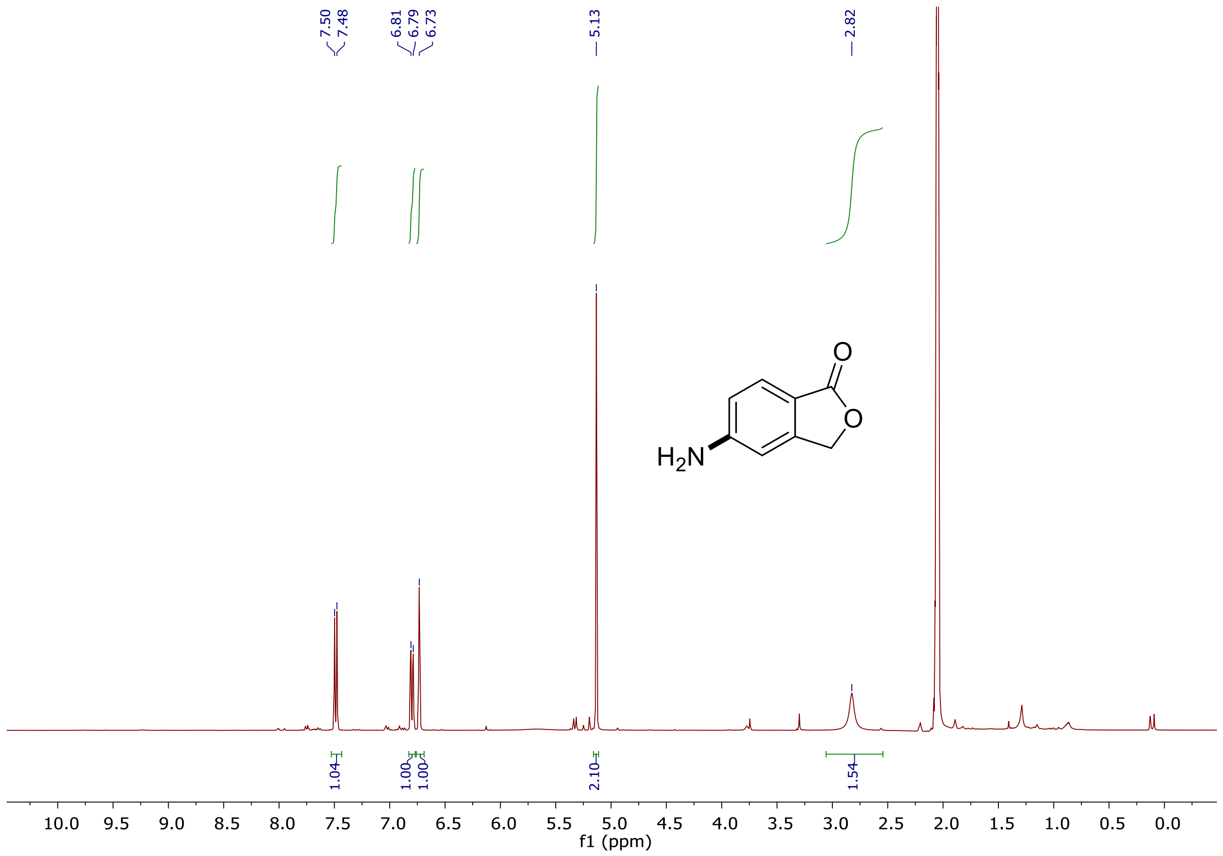
7.50
7.48
6.81
6.79
6.73
5.13
2.82
H2N
O
O
1.04
1.00
1.00
2.10
1.54
10.0 9.5 9.0 8.5 8.0 7.5 7.0 6.5 6.0 5.5 5.0 4.5 4.0 3.5 3.0 2.5 2.0 1.5 1.0 0.5 0.0
f1 (ppm)

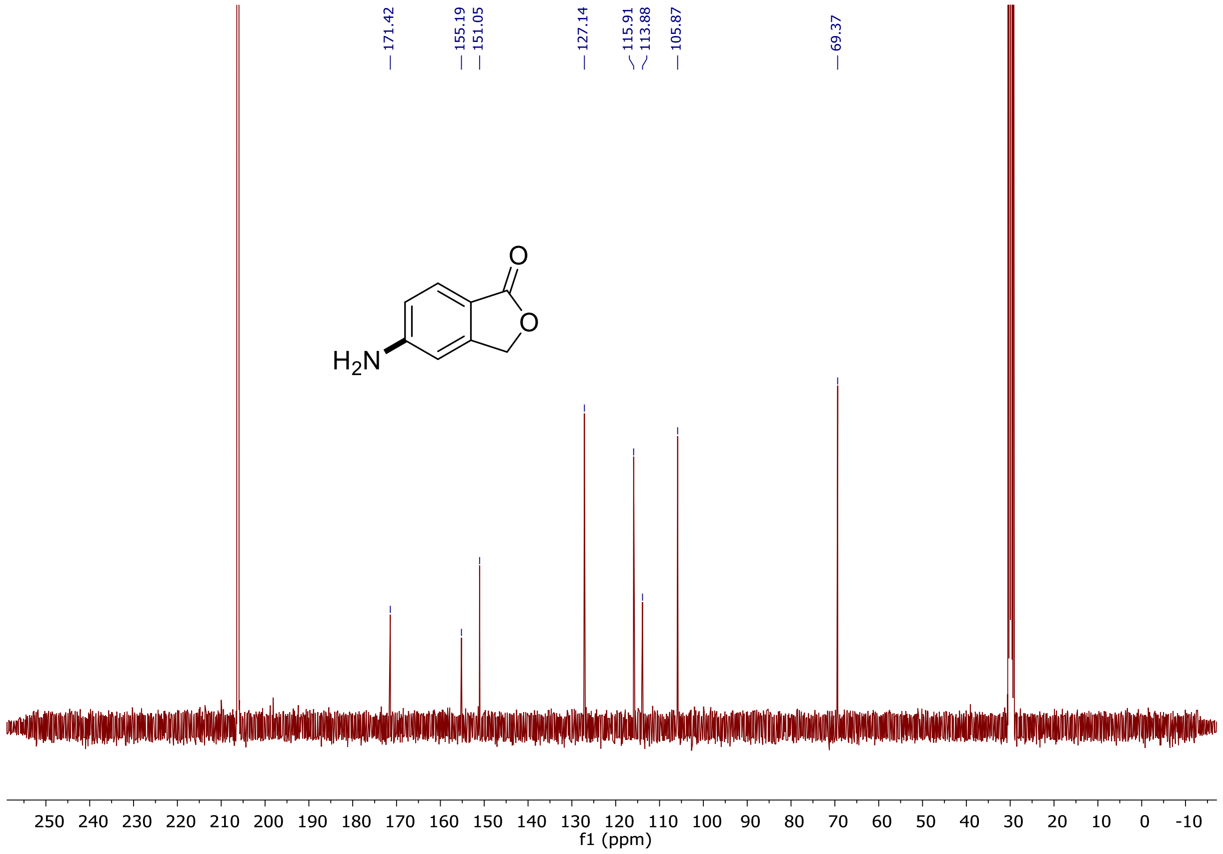
171.42
155.19
151.05
127.14
115.91
113.88
105.87
69.37
H2N
O
O
250 240 230 220 210 200 190 180 170 160 150 140 130 120 110 100 90 80 70 60 50 40 30 20 10 0 -10
f1 (ppm)

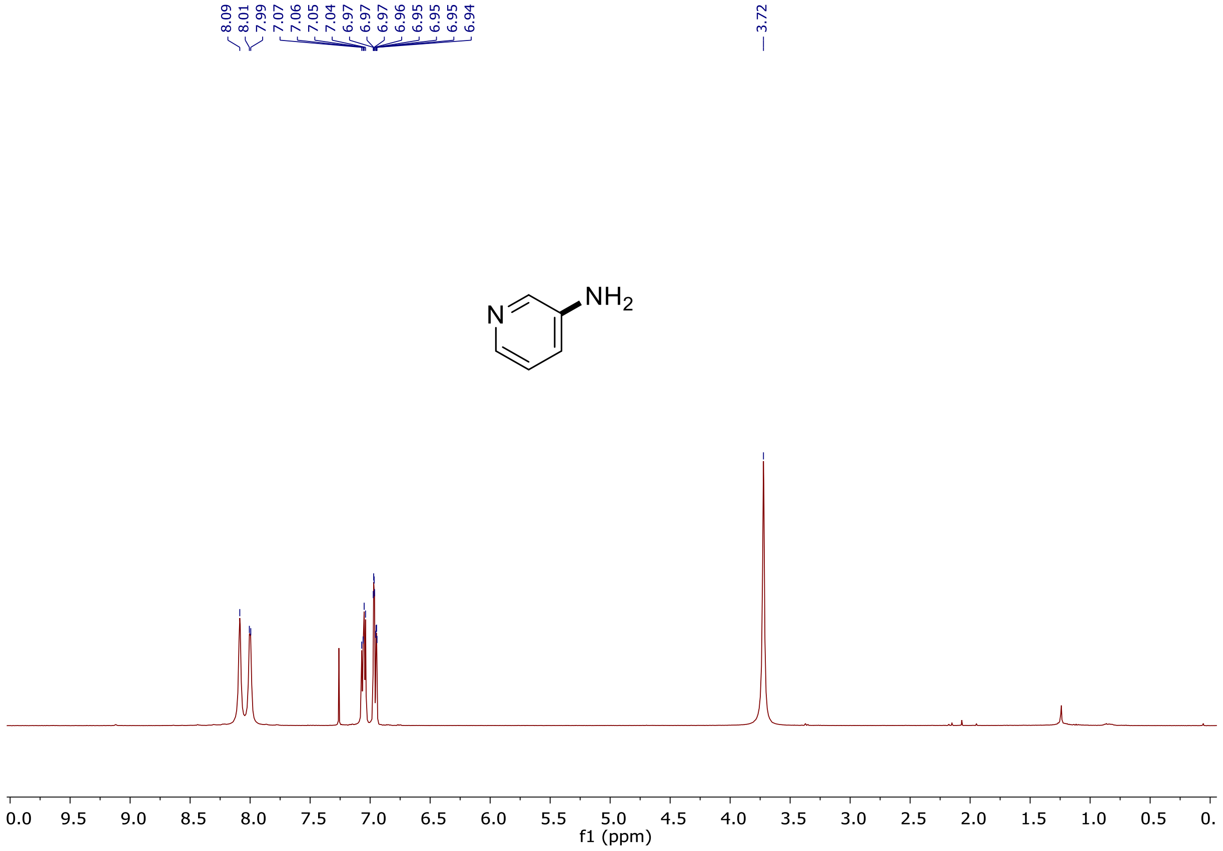
8.09
8.01
7.99
7.07
7.06
7.05
7.04
6.97
6.97
6.97
6.96
6.95
6.95
6.95
6.94
3.72
NH2
N
f1 (ppm)

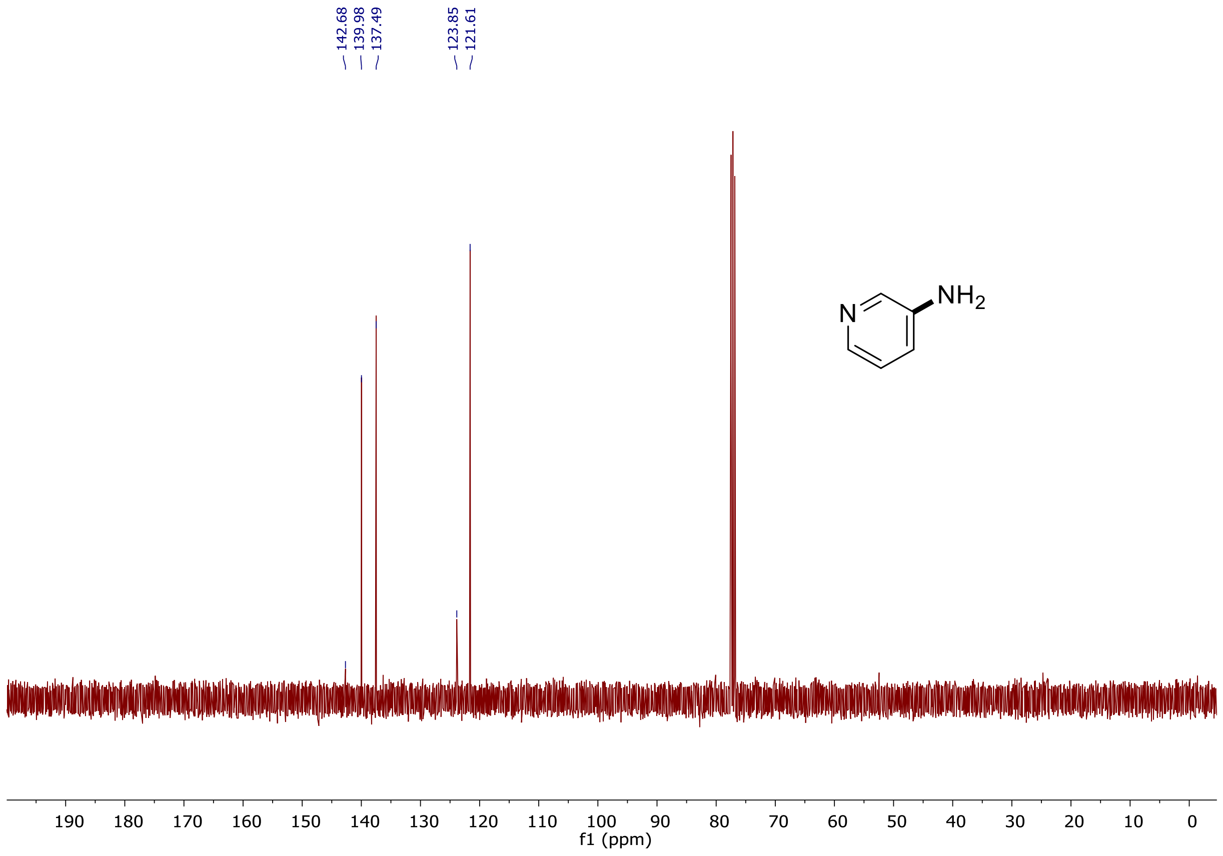
142.68
139.98
137.49
123.85
121.61
NH2
N
f1 (ppm)

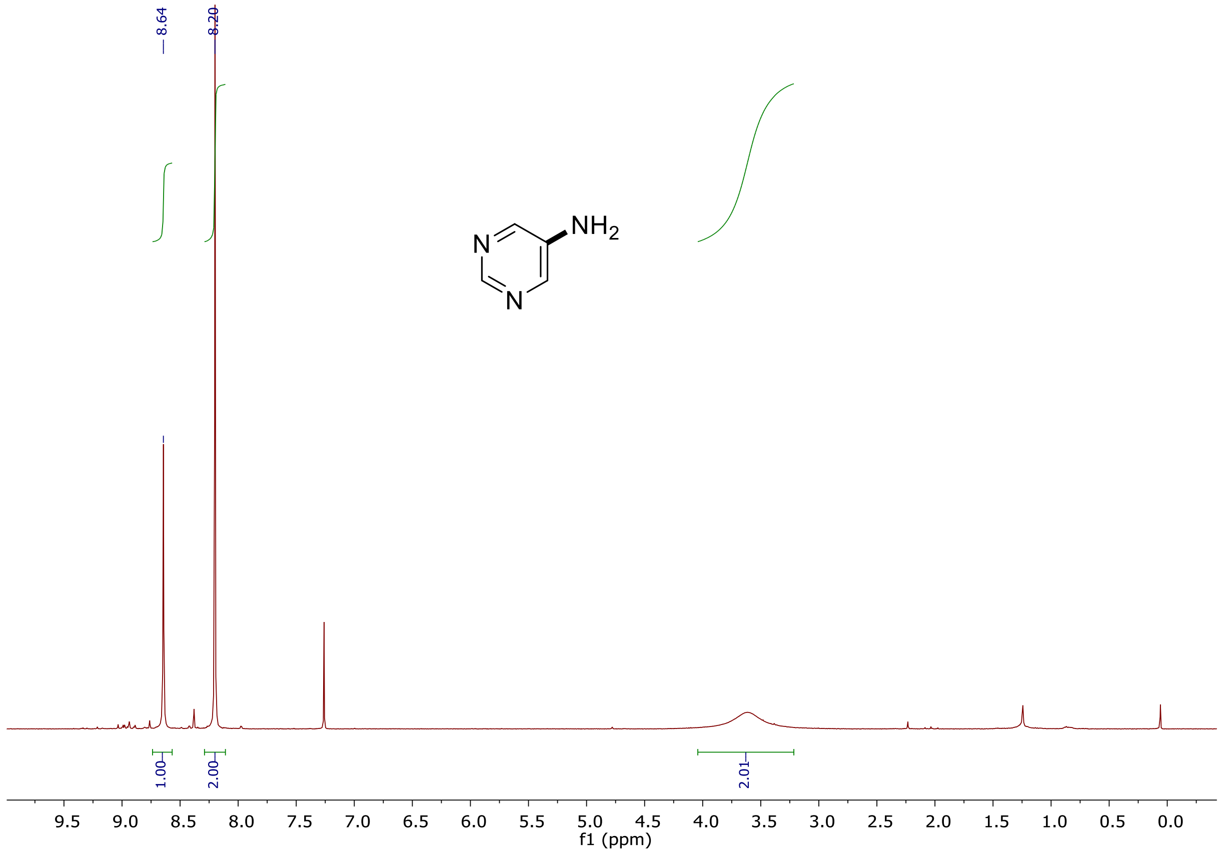
8.64
8.20
NH2
N
N
1.00
2.00
2.01
9.5
9.0
8.5
8.0
7.5
7.0
6.5
6.0
5.5
5.0
4.5
4.0
3.5
3.0
2.5
2.0
1.5
1.0
0.5
0.0
f1 (ppm)

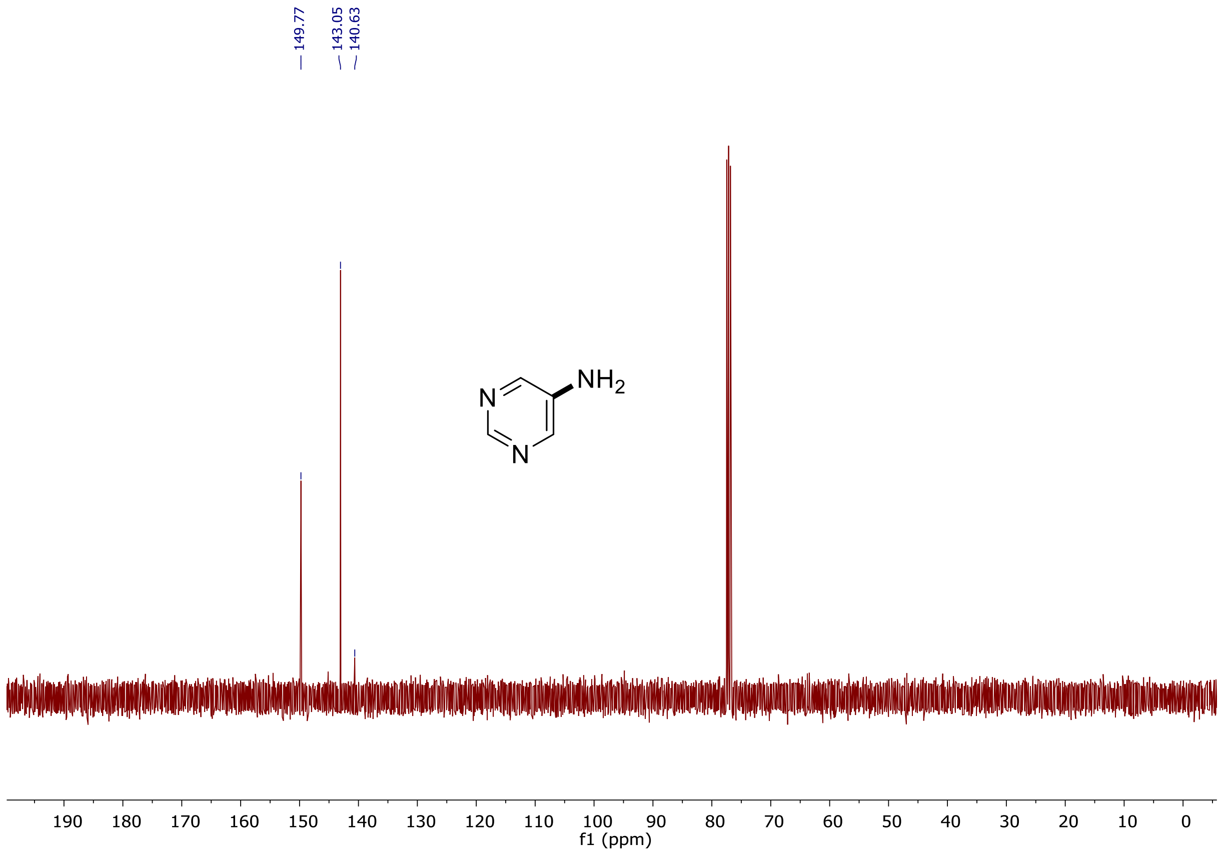
149.77
143.05
140.63
NH2
N
N
190
180
170
160
150
140
130
120
110
100
90
80
70
60
50
40
30
20
10
0
f1 (ppm)

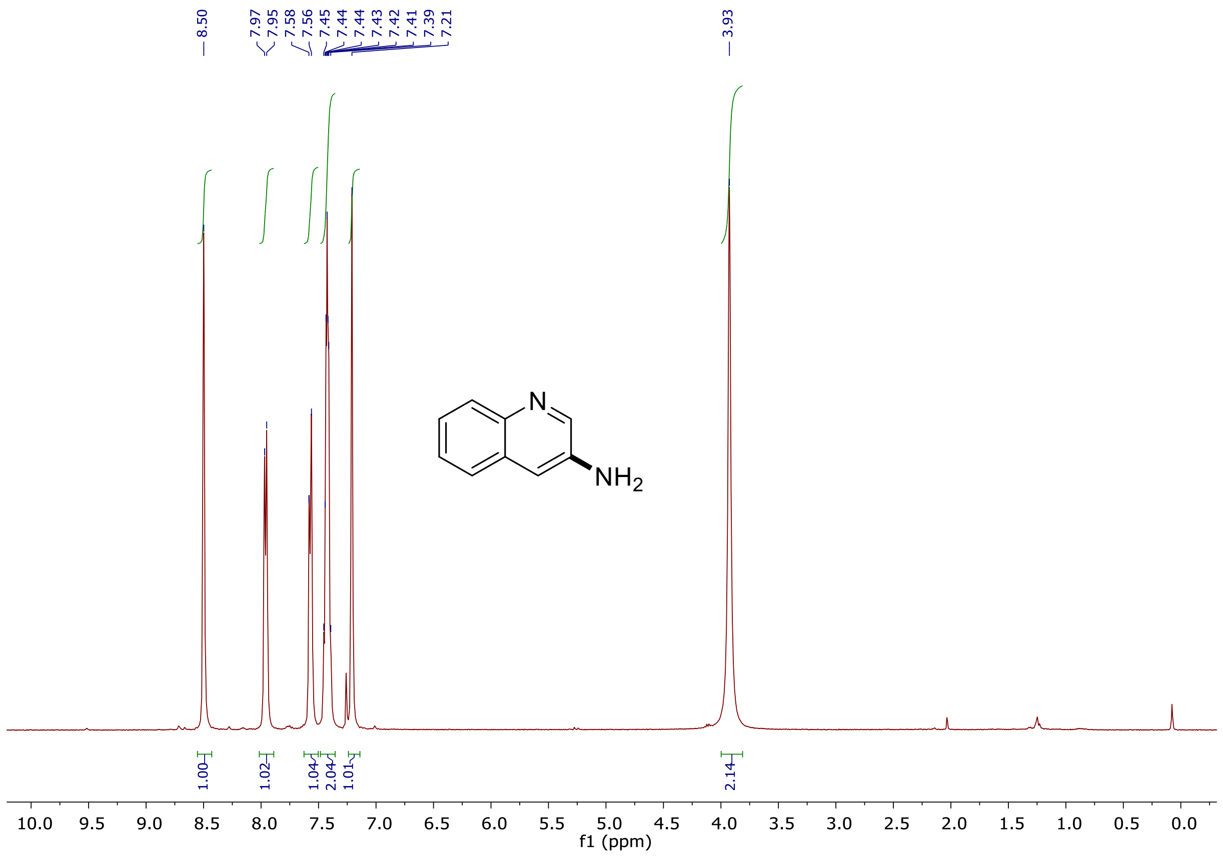
8.50
7.97
7.95
7.58
7.56
7.45
7.44
7.44
7.43
7.42
7.41
7.39
7.21
3.93
N
NH2
1.00
1.02
1.04
2.04
1.01
2.14
10.0 9.5 9.0 8.5 8.0 7.5 7.0 6.5 6.0 5.5 5.0 4.5 4.0 3.5 3.0 2.5 2.0 1.5 1.0 0.5 0.0
f1 (ppm)

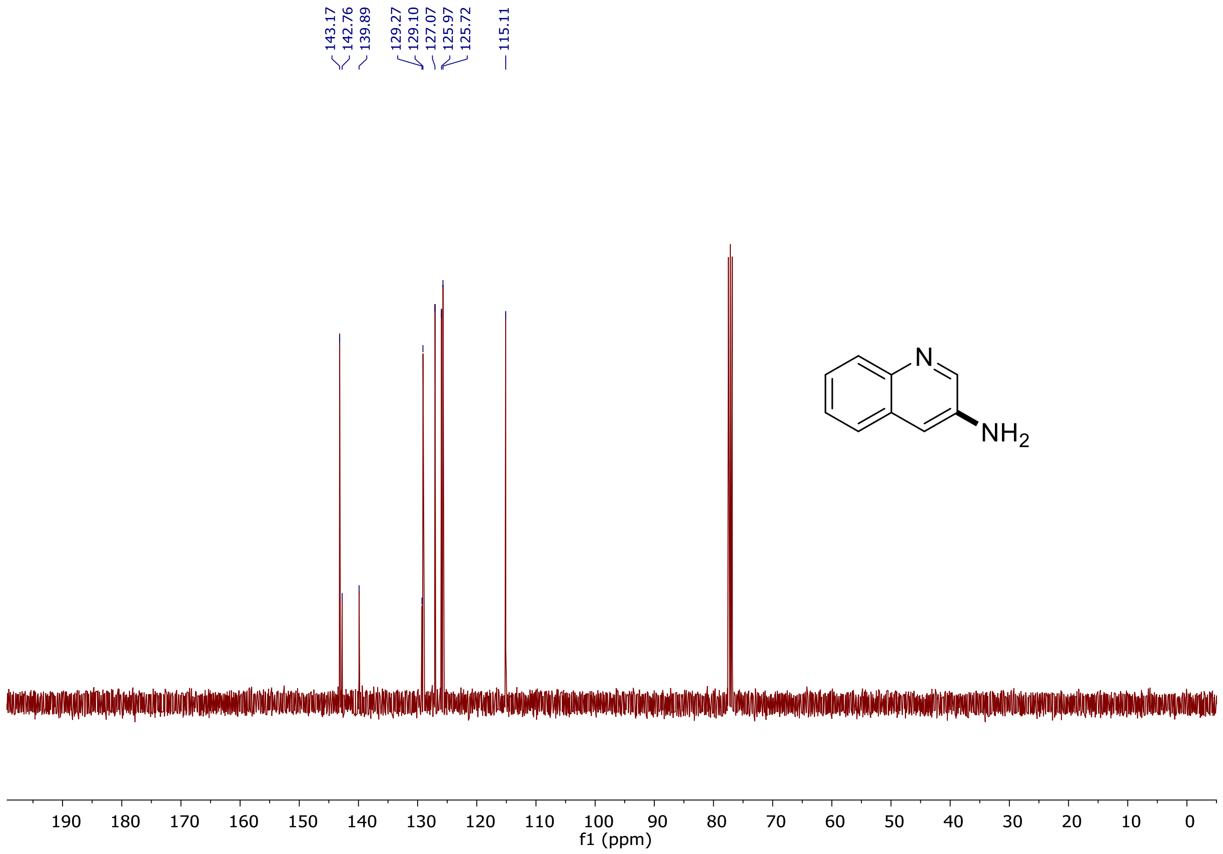
143.17
142.76
139.89
129.27
129.10
127.07
125.97
125.72
115.11
N
NH2
190 180 170 160 150 140 130 120 110 100 90 80 70 60 50 40 30 20 10 0
f1 (ppm)

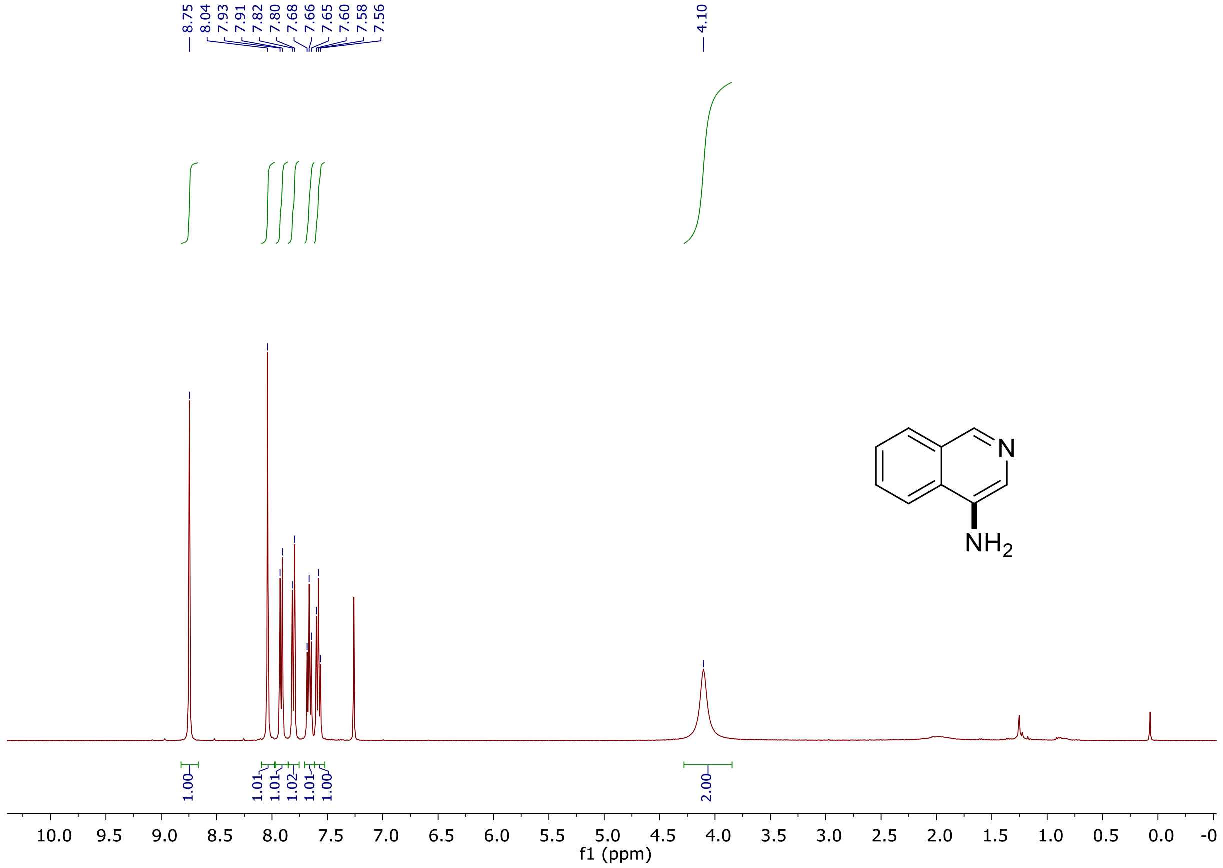
8.75
8.04
7.93
7.91
7.82
7.80
7.68
7.66
7.65
7.60
7.58
7.56
4.10
1.00
1.01
1.01
1.02
1.01
1.00
2.00
NH2
N
10.0 9.5 9.0 8.5 8.0 7.5 7.0 6.5 6.0 5.5 5.0 4.5 4.0 3.5 3.0 2.5 2.0 1.5 1.0 0.5 0.0
f1 (ppm)

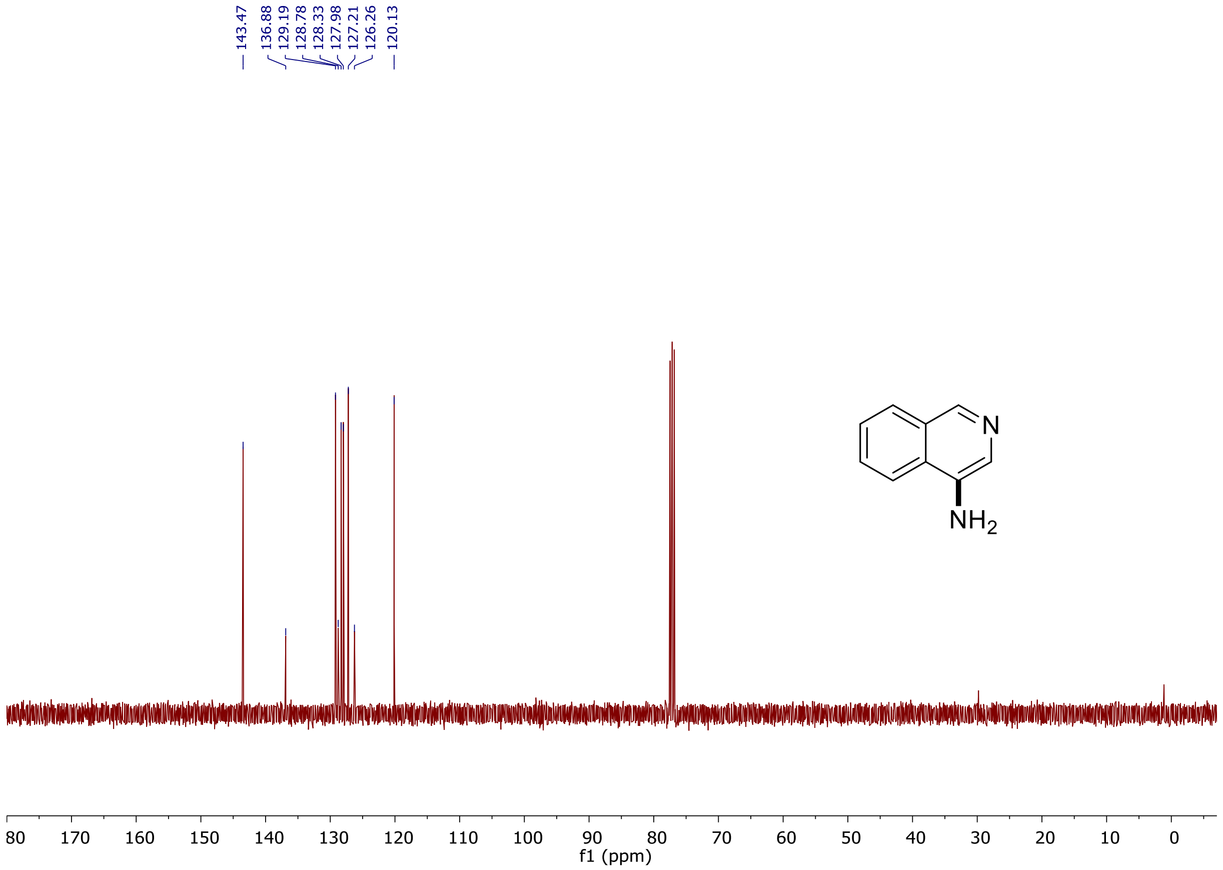
143.47
136.88
129.19
128.78
128.33
127.98
127.21
126.26
120.13
NH2
N
170 160 150 140 130 120 110 100 90 80 70 60 50 40 30 20 10 0
f1 (ppm)

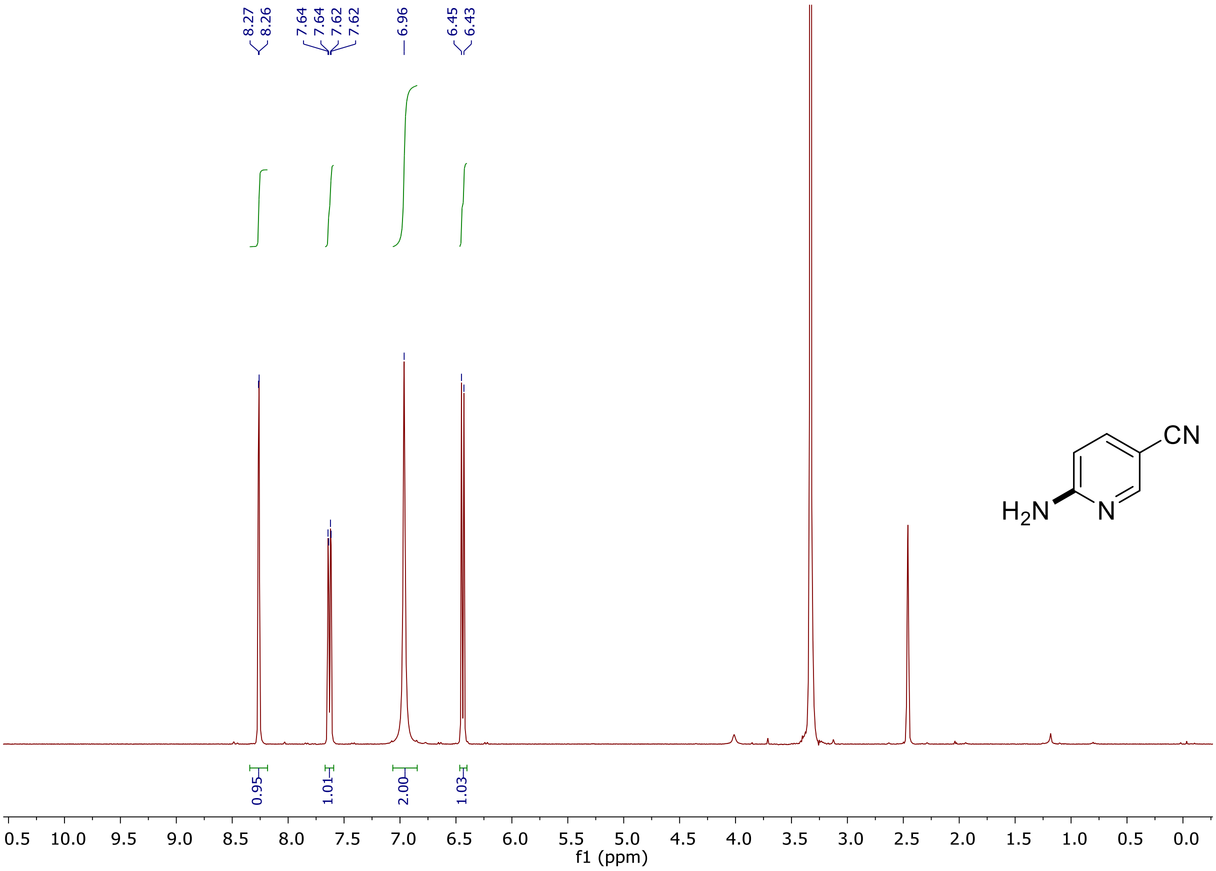

8.27
8.26
7.64
7.64
7.62
7.62
6.96
6.45
6.43
0.95
1.01
2.00
1.03
$H_2N$
N
CN
0.5 10.0 9.5 9.0 8.5 8.0 7.5 7.0 6.5 6.0 5.5 5.0 4.5 4.0 3.5 3.0 2.5 2.0 1.5 1.0 0.5 0.0
f1 (ppm)


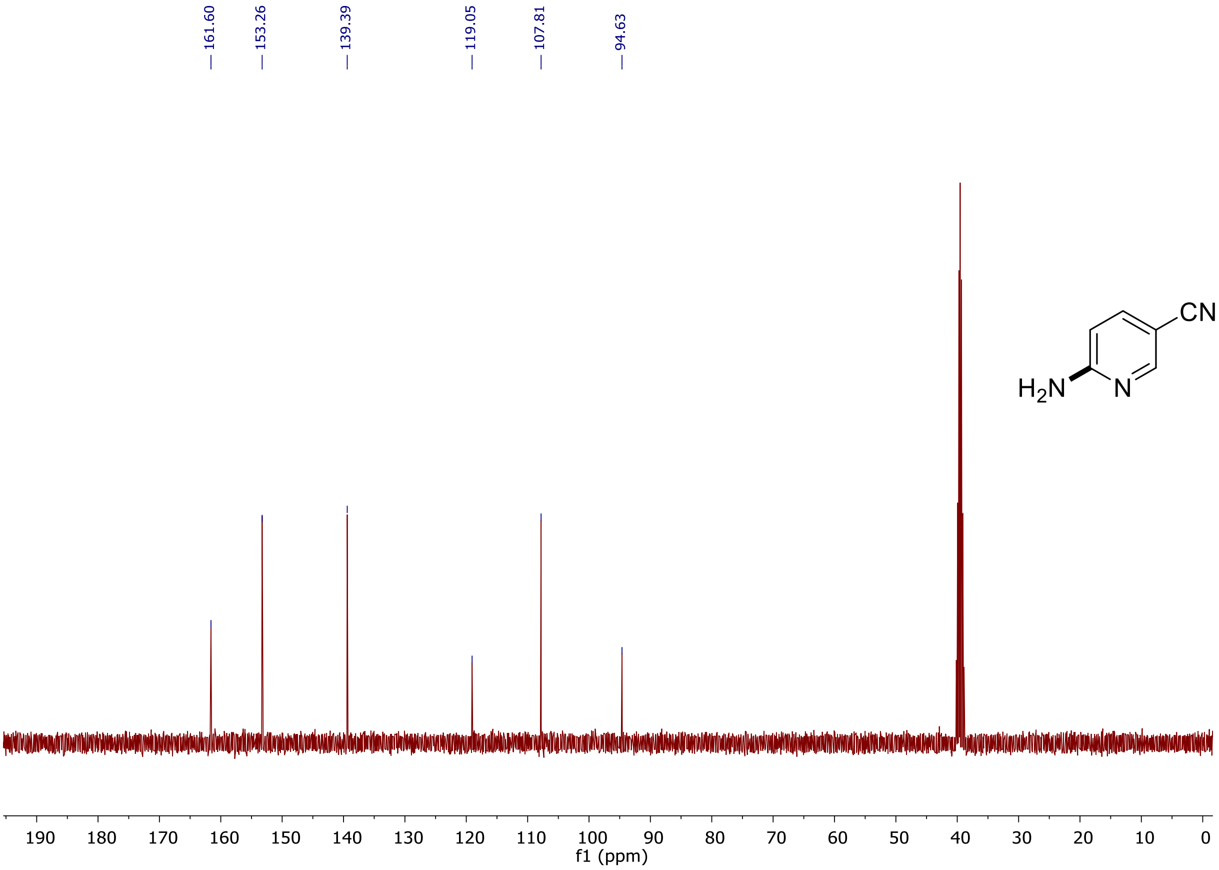

161.60
153.26
139.39
119.05
107.81
94.63
$H_2N$
N
CN
190 180 170 160 150 140 130 120 110 100 90 80 70 60 50 40 30 20 10 0
f1 (ppm)

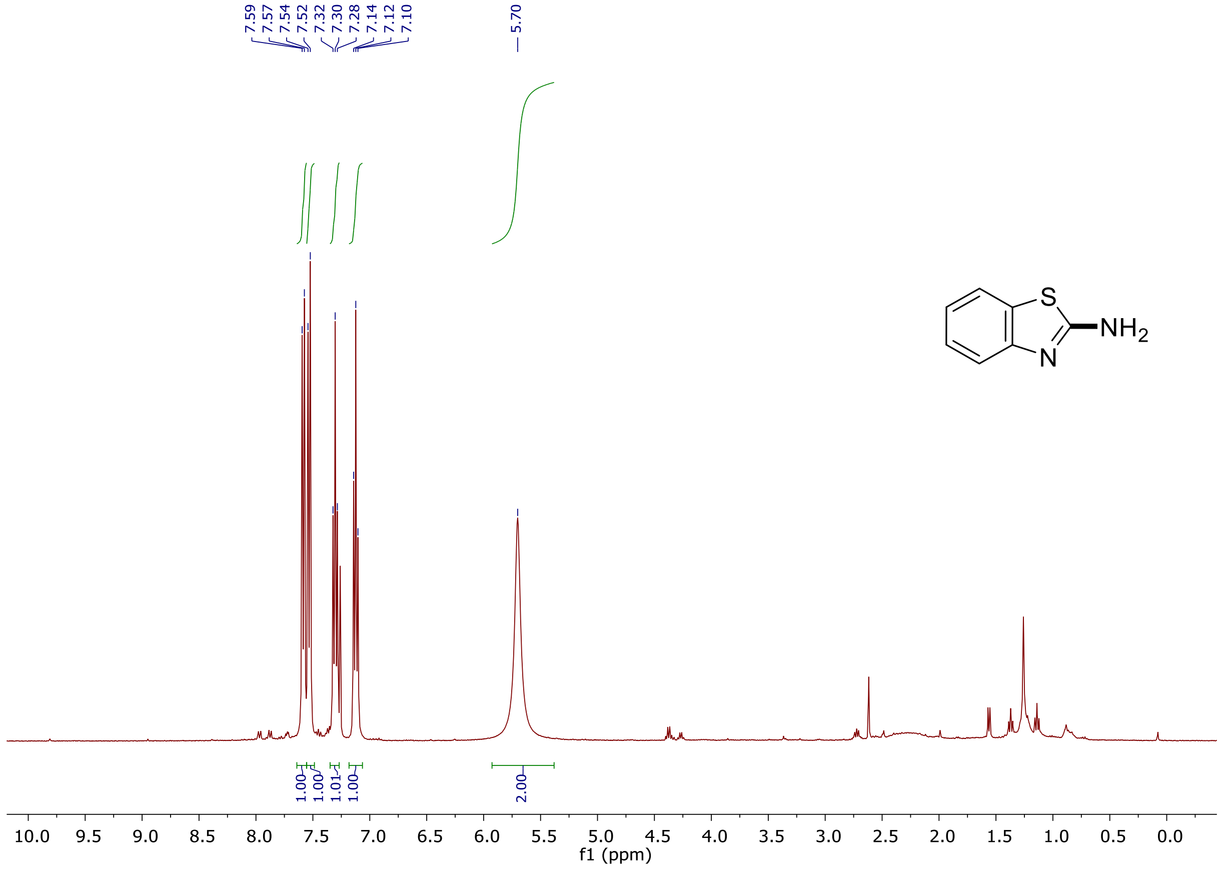
7.59
7.57
7.54
7.52
7.32
7.30
7.28
7.14
7.12
7.10
5.70
S
N
NH2
1.00
1.00
1.01
1.00
2.00
10.0 9.5 9.0 8.5 8.0 7.5 7.0 6.5 6.0 5.5 5.0 4.5 4.0 3.5 3.0 2.5 2.0 1.5 1.0 0.5 0.0
f1 (ppm)

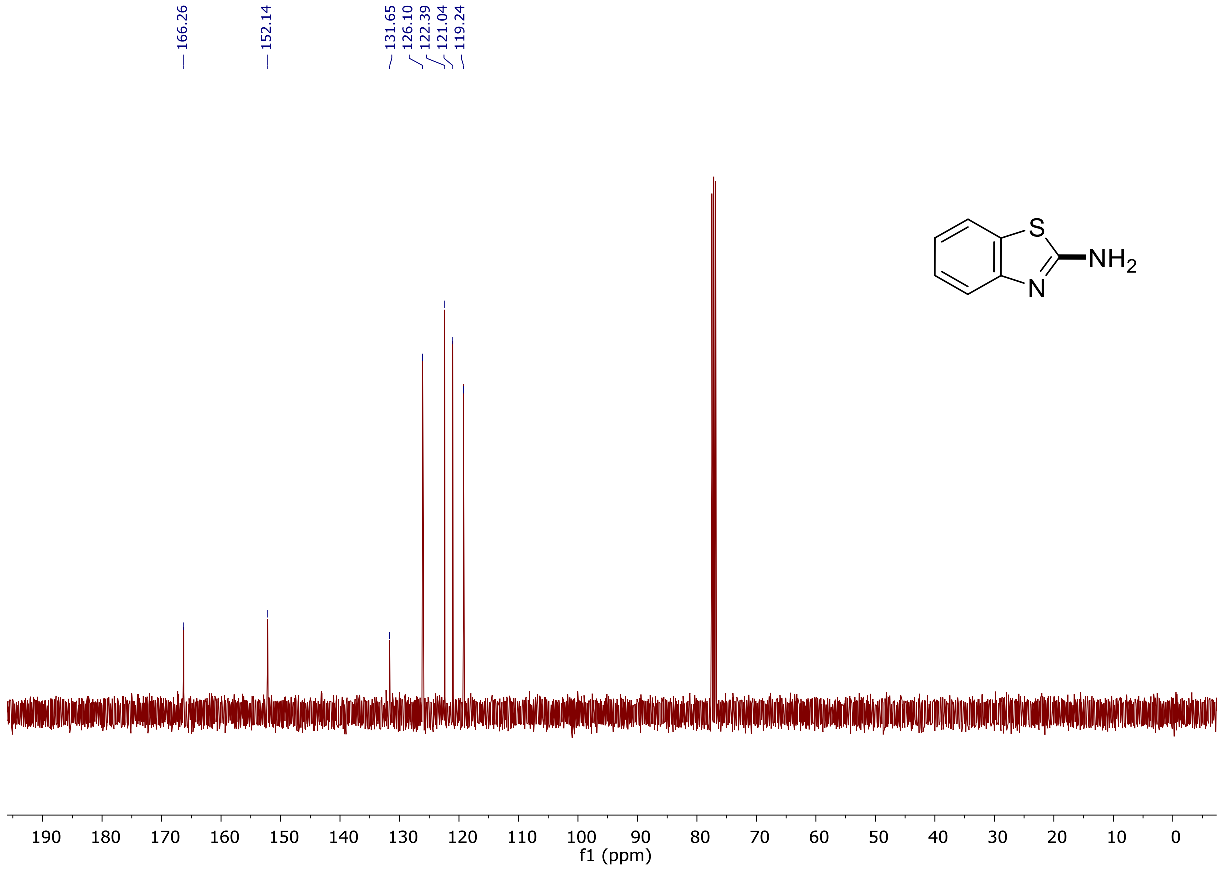
166.26
152.14
131.65
126.10
122.39
121.04
119.24
S
N
NH2
190 180 170 160 150 140 130 120 110 100 90 80 70 60 50 40 30 20 10 0
f1 (ppm)

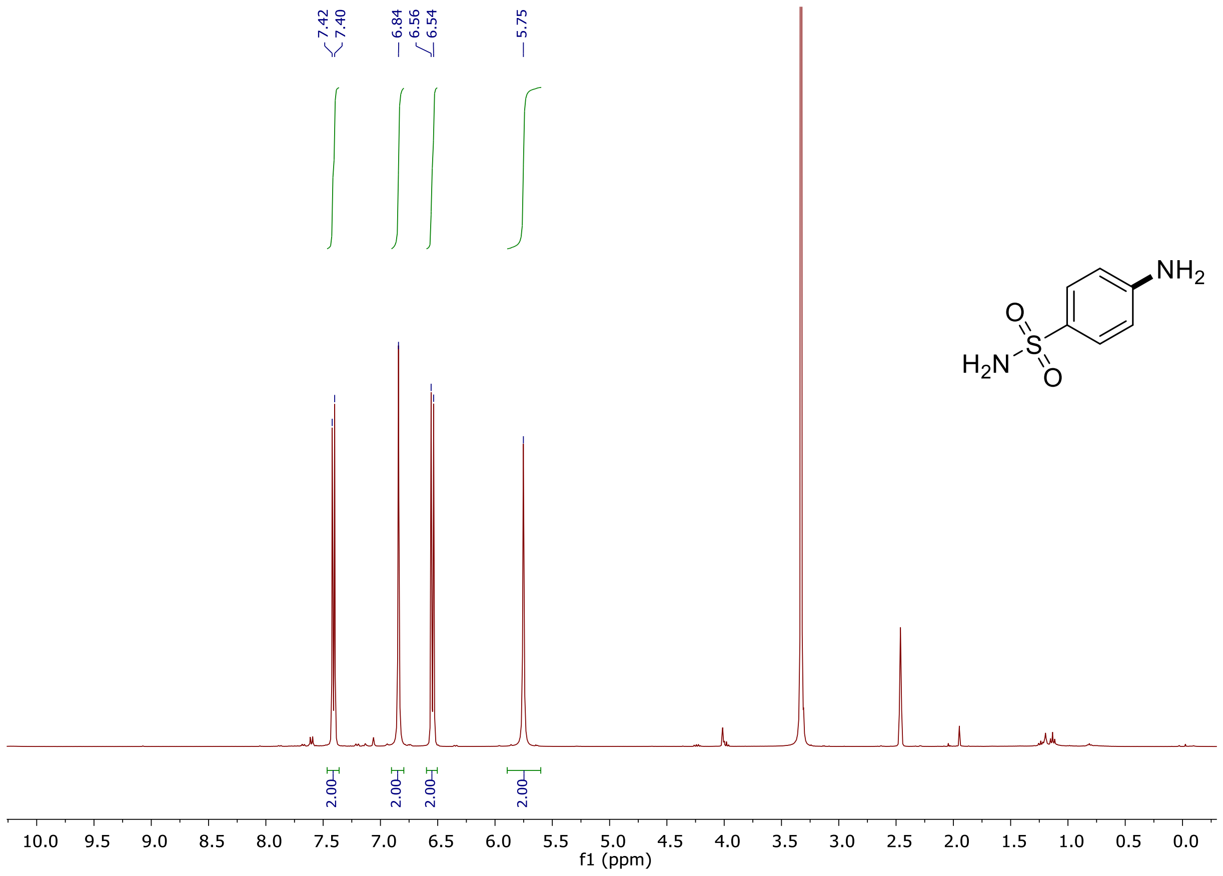
7.42
7.40
6.84
6.56
6.54
5.75
2.00
2.00
2.00
2.00
10.0 9.5 9.0 8.5 8.0 7.5 7.0 6.5 6.0 5.5 5.0 4.5 4.0 3.5 3.0 2.5 2.0 1.5 1.0 0.5 0.0
f1 (ppm)
NH2
O
S
H2N
O

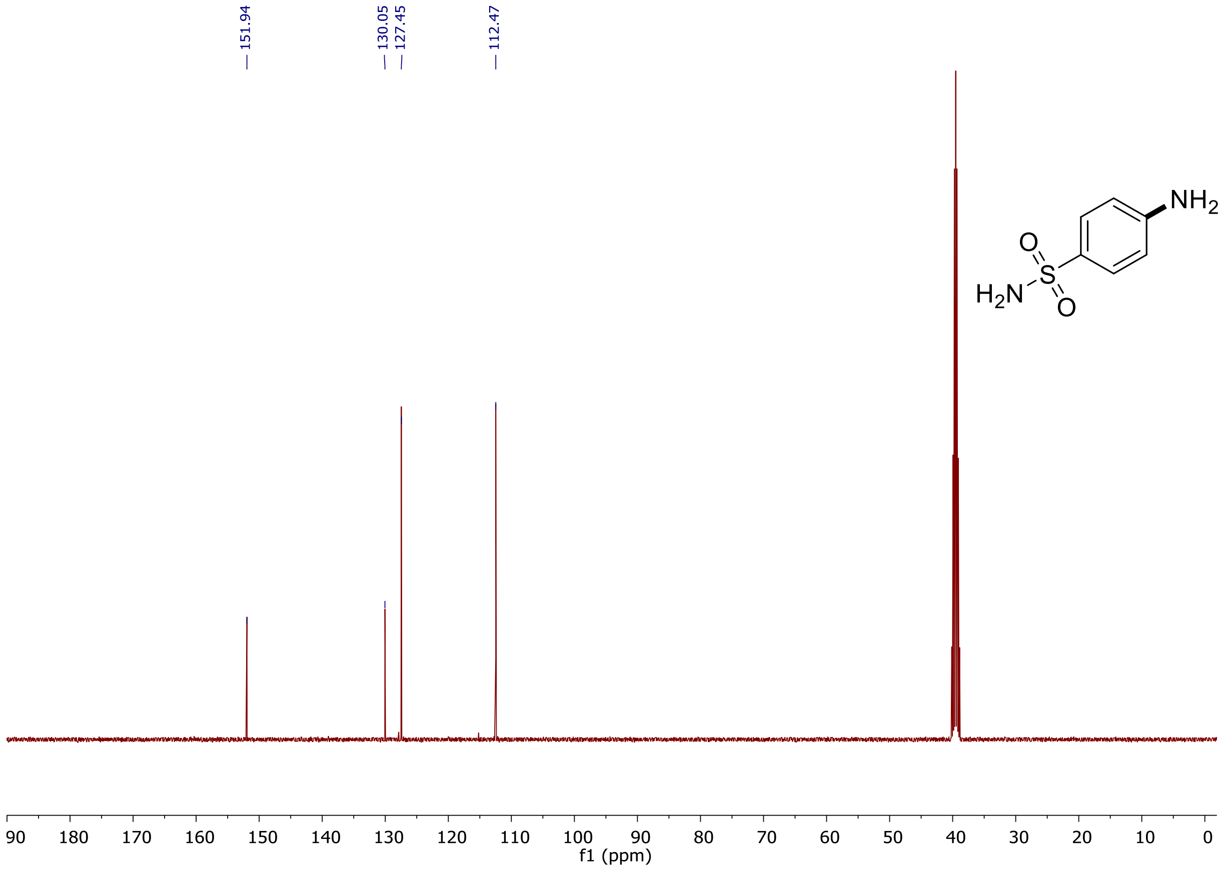
151.94
130.05
127.45
112.47
90 180 170 160 150 140 130 120 110 100 90 80 70 60 50 40 30 20 10 0
f1 (ppm)
NH2
O
S
H2N
O

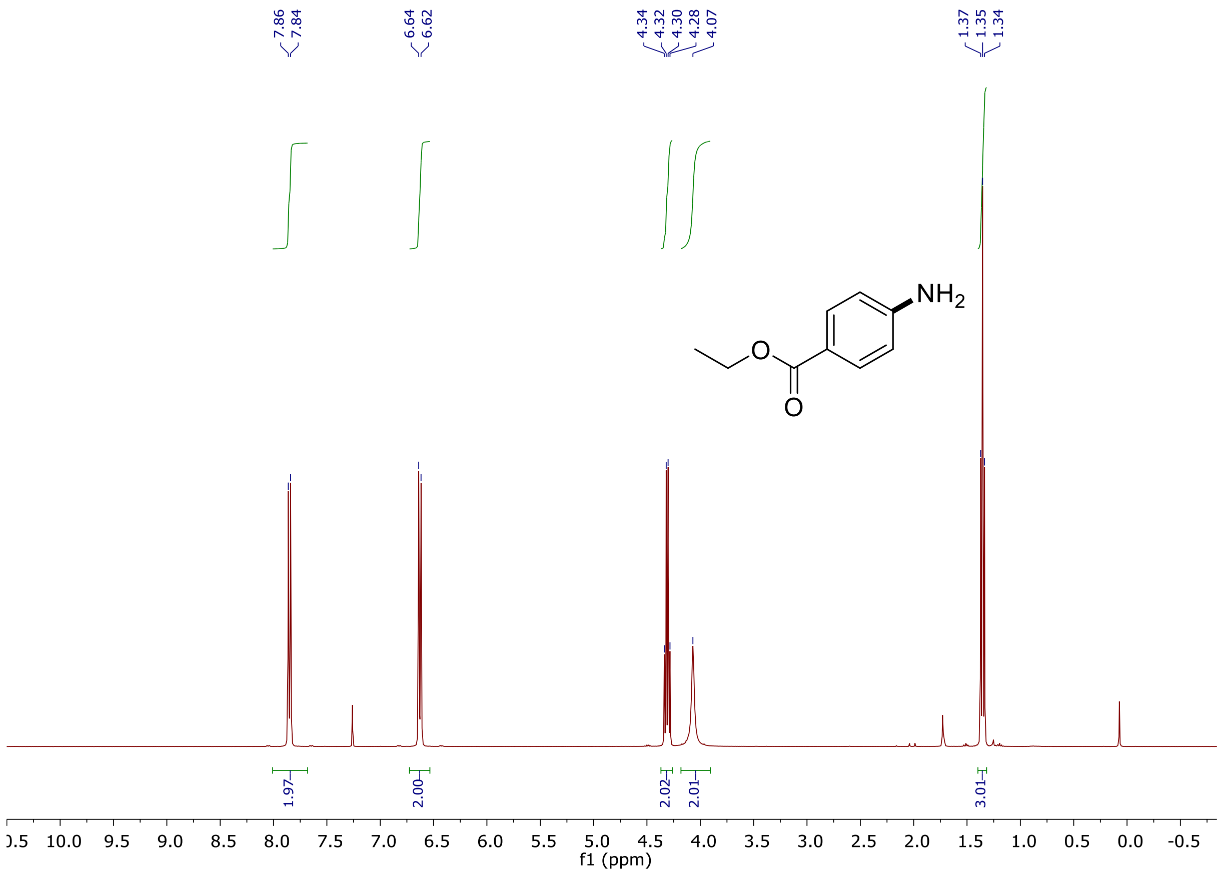
7.86
7.84
6.64
6.62
4.34
4.32
4.30
4.28
4.07
1.37
1.35
1.34
NH2
O
O
1.97
2.00
2.02
2.01
3.01
f1 (ppm)

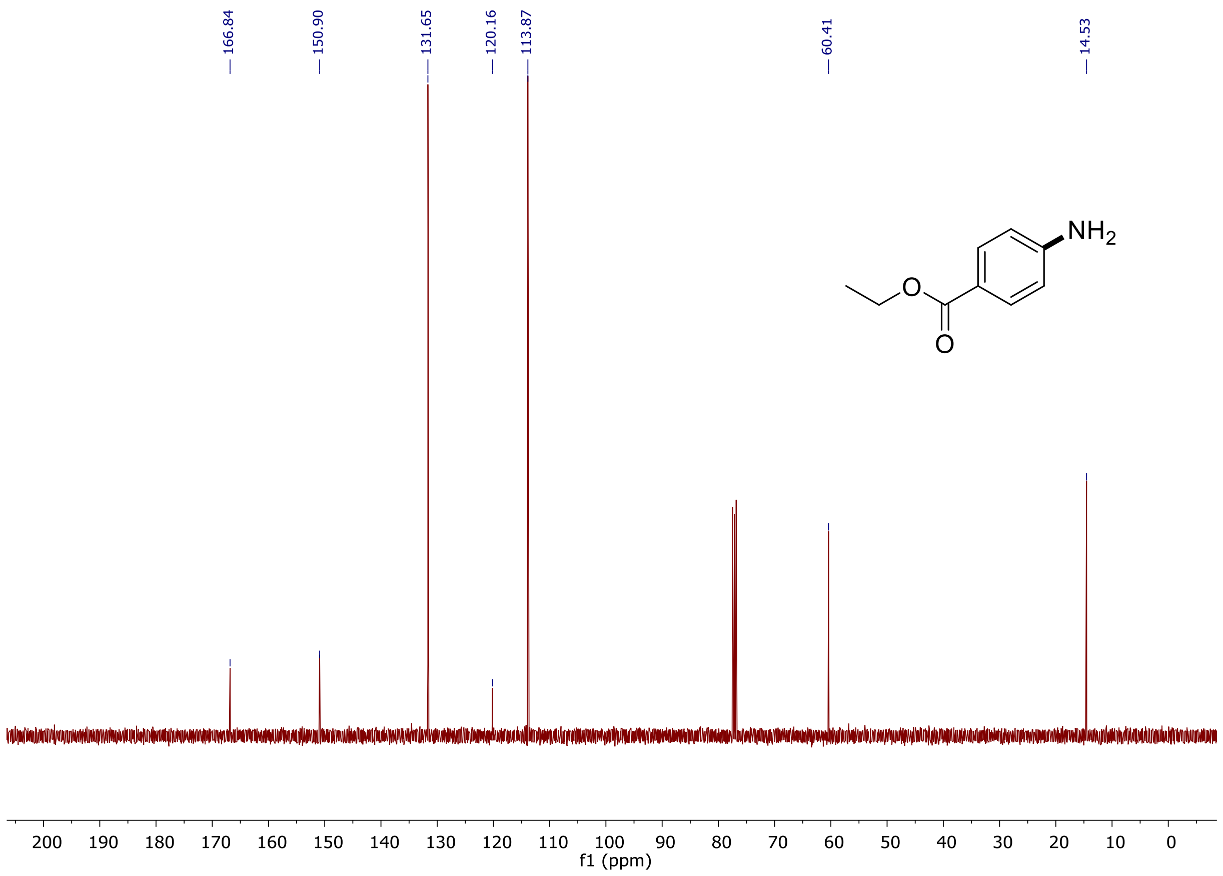
166.84
150.90
131.65
120.16
113.87
60.41
14.53
NH2
O
O
f1 (ppm)

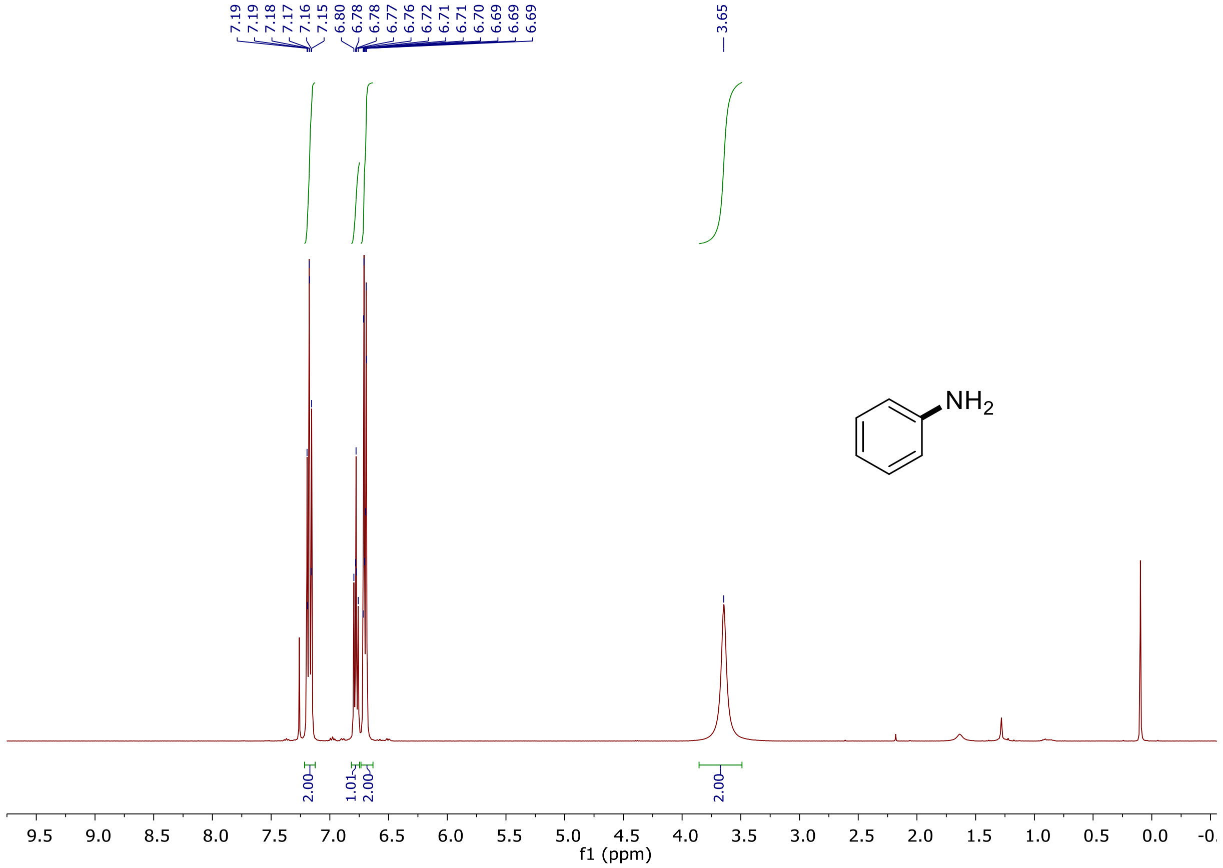
7.19
7.19
7.18
7.17
7.16
7.15
6.80
6.78
6.78
6.77
6.76
6.72
6.71
6.71
6.70
6.69
6.69
6.69
3.65
NH2
2.00
1.01
2.00
2.00
f1 (ppm)

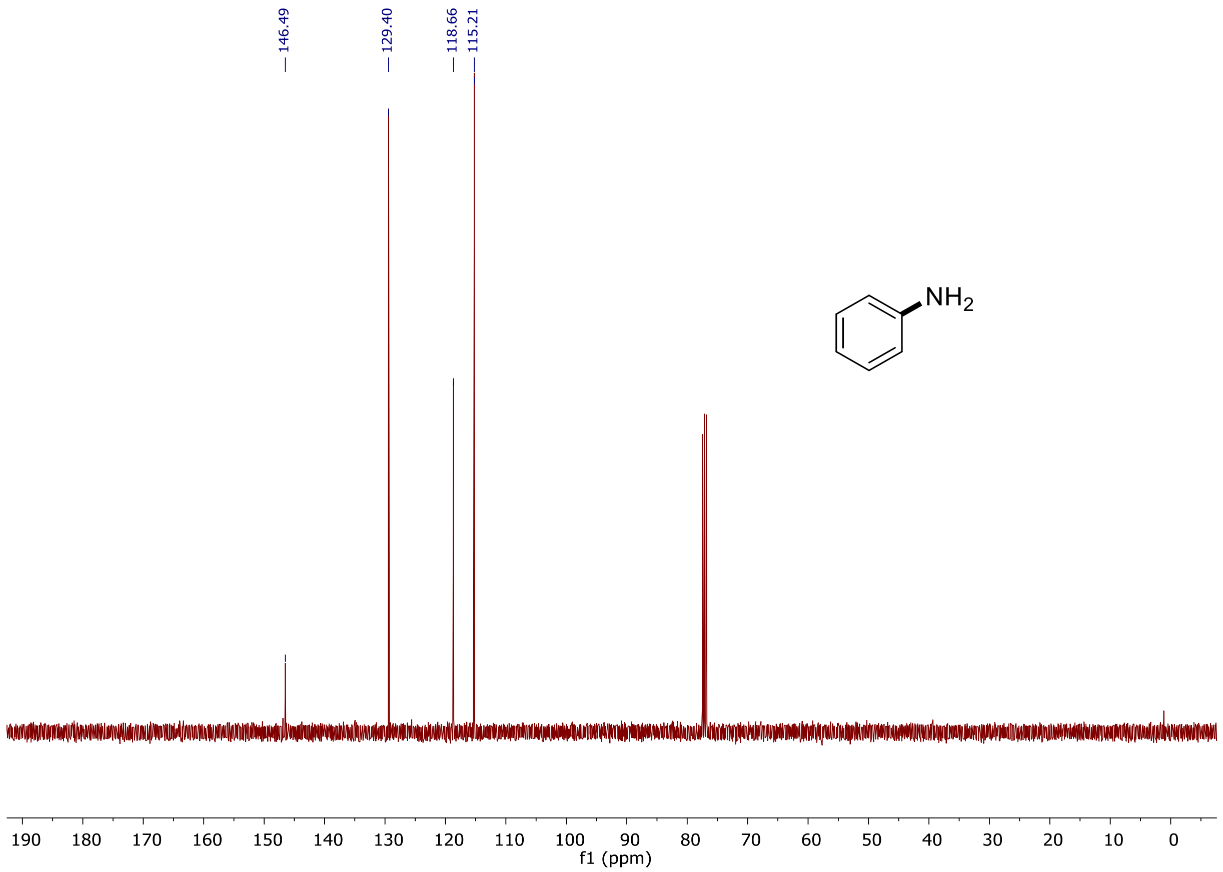
146.49
129.40
118.66
115.21
NH2
f1 (ppm)

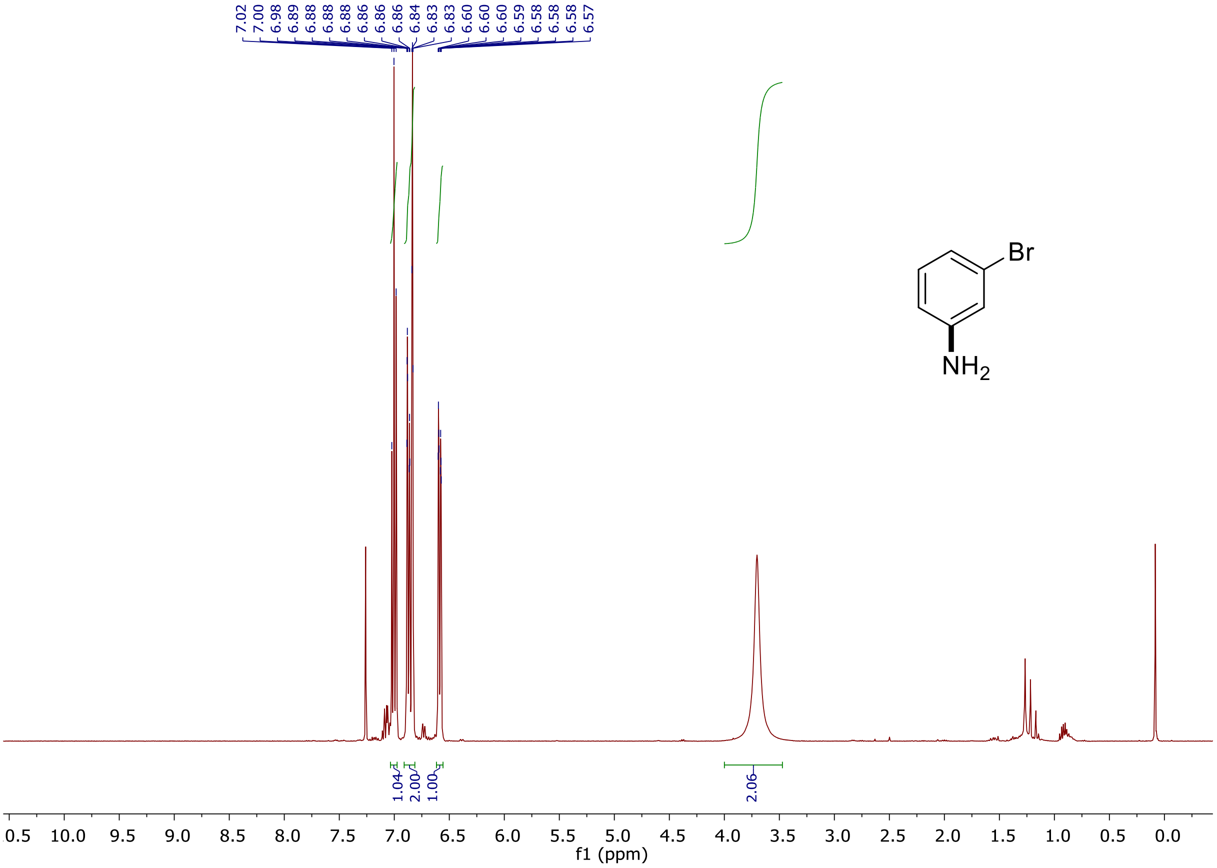
7.02
7.00
6.98
6.89
6.88
6.88
6.88
6.86
6.86
6.86
6.84
6.83
6.83
6.60
6.60
6.60
6.59
6.58
6.58
6.58
6.57
Br
NH2
1.04
2.00
1.00
2.06
0.5 10.0 9.5 9.0 8.5 8.0 7.5 7.0 6.5 6.0 5.5 5.0 4.5 4.0 3.5 3.0 2.5 2.0 1.5 1.0 0.5 0.0
f1 (ppm)

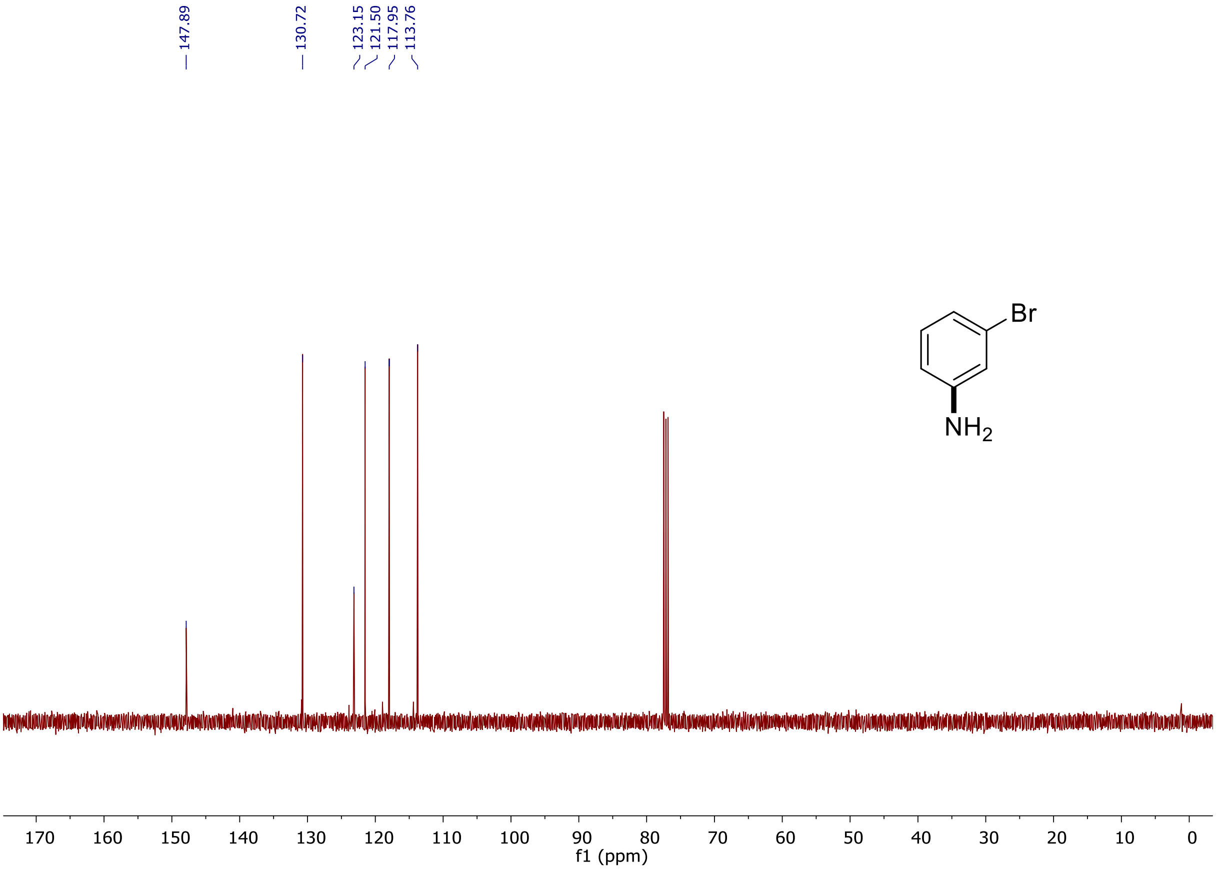
147.89
130.72
123.15
121.50
117.95
113.76
Br
NH2
170 160 150 140 130 120 110 100 90 80 70 60 50 40 30 20 10 0
f1 (ppm)

## 7. Cartesian coordinates.

**$Ni^{II}$-mpg-$CN_x$** (singlet, G = -3678.562365)

| | | | |
|---|---|---|---|
| H | -7.530275 | 4.064849 | 0.711427 |
| N | -6.619653 | 4.498292 | 0.623341 |
| C | -5.540553 | 3.752805 | 0.406007 |
| N | -5.73406 | 2.410979 | 0.313641 |
| C | -4.679642 | 1.67651 | 0.028789 |
| N | -3.438992 | 2.306304 | -0.182591 |
| C | -3.292776 | 3.687121 | 0.038496 |
| N | -4.354273 | 4.411808 | 0.317772 |
| N | -2.490446 | 0.26237 | -0.832593 |
| C | -3.619246 | -0.290052 | -0.38747 |
| N | -4.736313 | 0.332389 | -0.01575 |
| C | -2.355906 | 1.560307 | -0.619105 |
| N | -1.166223 | 2.130963 | -0.762273 |
| C | -1.069187 | 3.391249 | -0.336911 |
| N | -2.056444 | 4.218804 | -0.002038 |
| N | 0.222738 | 3.905319 | -0.235675 |
| C | 1.375735 | 3.192083 | -0.085898 |
| N | 2.523934 | 3.866141 | -0.192329 |
| C | 3.689874 | 3.202373 | -0.103693 |
| N | 3.646771 | 1.810094 | 0.101109 |
| N | 1.285831 | 1.859507 | 0.201236 |
| C | 2.440567 | 1.238718 | 0.255548 |
| C | 4.803348 | 1.001188 | 0.104967 |
| N | 4.865818 | 3.771695 | -0.219554 |
| C | 5.955027 | 2.954287 | -0.155224 |
| N | 7.140656 | 3.533527 | -0.251269 |
| N | 5.961528 | 1.58986 | -0.013602 |
| N | 4.661913 | -0.34515 | 0.199725 |
| C | 3.443965 | -0.843856 | 0.316949 |
| N | 2.339305 | -0.074805 | 0.451705 |
| N | 3.24625 | -2.202956 | 0.315395 |
| H | 4.076277 | -2.792016 | 0.266029 |
| N | 0.911745 | -2.082872 | 0.224583 |
| C | 2.0283 | -2.848005 | 0.249041 |
| N | 2.013082 | -4.167976 | 0.203789 |
| C | 0.819626 | -4.799575 | 0.091028 |
| N | 0.711981 | -6.099756 | 0.042807 |
| C | -0.551911 | -6.613499 | -0.079121 |
| N | -1.717057 | -5.915512 | -0.161896 |
| N | -0.642109 | -7.933936 | -0.121172 |
| N | -0.355421 | -4.025069 | 0.022391 |
| C | -0.279975 | -2.677878 | 0.106953 |
| C | -1.631831 | -4.605909 | -0.115381 |
| N | -2.697818 | -3.793948 | -0.215993 |
| C | -2.507215 | -2.473818 | -0.149682 |
| N | -1.308626 | -1.870675 | 0.0747 |
| N | -3.613552 | -1.67978 | -0.288243 |
| H | -6.534063 | 5.502449 | 0.718909 |
| H | 0.317015 | 4.91798 | -0.210465 |
| H | 7.211813 | 4.538 | -0.365802 |
| H | 7.986388 | 2.976254 | -0.214229 |
| H | -4.514824 | -2.146304 | -0.218761 |
| H | 0.192085 | -8.5058 | -0.059336 |
| H | -1.547284 | -8.38141 | -0.205761 |
| Ni | 0.570348 | -0.293129 | 0.178776 |

**$Ni^{I}$-mpg-$CN_x$** (doublet, G = -3678.757249)

| | | | |
|---|---|---|---|
| H | -7.525530 | -4.044609 | 0.1039083 |
| N | -6.613150 | -4.482687 | 0.0894385 |
| C | -5.512033 | -3.735316 | 0.0599232 |
| N | -5.691098 | -2.389766 | 0.0389907 |
| C | -4.605073 | -1.643842 | 0.0212072 |
| N | -3.342458 | -2.262529 | 0.0329085 |
| C | -3.233389 | -3.664372 | 0.0275408 |
| N | -4.327518 | -4.398383 | 0.0454183 |
| N | -2.280750 | -0.161683 | 0.0525033 |
| C | -3.514442 | 0.3609561 | -0.040200 |
| N | -4.671770 | -0.305318 | -0.030885 |
| C | -2.197386 | -1.485090 | 0.0557383 |
| N | -0.998149 | -2.051009 | 0.0586923 |
| C | -0.967463 | -3.390828 | -0.028601 |
| N | -2.013852 | -4.220548 | -0.018747 |
| N | 0.2710200 | -4.011696 | -0.109337 |
| C | 1.4759025 | -3.368172 | -0.290443 |
| N | 2.5837375 | -3.965441 | 0.1568068 |
| C | 3.7100292 | -3.230125 | 0.1494516 |
| N | 3.6163998 | -1.862972 | -0.186835 |
| N | 1.4011968 | -2.167555 | -0.881692 |
| C | 2.4324031 | -1.381175 | -0.666214 |
| C | 4.7054590 | -0.987992 | -0.012992 |
| N | 4.8808001 | -3.713887 | 0.5028522 |
| C | 5.9196485 | -2.834348 | 0.5523705 |
| N | 7.1108832 | -3.344258 | 0.8559282 |
| N | 5.8677784 | -1.491737 | 0.3513006 |
| N | 4.5106945 | 0.3248462 | -0.199861 |
| C | 3.2865699 | 0.7314170 | -0.550872 |
| N | 2.2511218 | -0.071562 | -0.869802 |
| N | 3.0796389 | 2.0955228 | -0.551922 |
| H | 3.8905835 | 2.6467265 | -0.281461 |
| N | 0.7607340 | 2.1241617 | -0.876013 |
| C | 1.8882729 | 2.7913930 | -0.556632 |
| N | 1.9612876 | 4.0805236 | -0.210493 |
| C | 0.8124708 | 4.7466570 | -0.029013 |
| N | 0.7722368 | 6.0142097 | 0.3303382 |
| C | -0.457003 | 6.5586131 | 0.5261345 |
| N | -1.657714 | 5.9174622 | 0.4757963 |
| N | -0.492139 | 7.8550528 | 0.8247415 |
| N | -0.402180 | 4.0566599 | -0.203358 |
| C | -0.390087 | 2.7766951 | -0.678120 |
| C | -1.639166 | 4.6495011 | 0.1271666 |
| N | -2.738272 | 3.8741068 | 0.1343238 |
| C | -2.590840 | 2.6224342 | -0.307935 |
| N | -1.500915 | 2.1077470 | -0.894340 |
| N | -3.634137 | 1.7408161 | -0.126485 |
| H | -6.542934 | -5.492271 | 0.1085383 |
| H | 0.2913556 | -5.003644 | 0.1101870 |
| H | 7.2108483 | -4.335767 | 1.0324591 |
| H | 7.9204276 | -2.740711 | 0.9244063 |
| H | -4.549142 | 2.1267870 | 0.0888276 |
| H | 0.3673563 | 8.3849757 | 0.8938544 |
| H | -1.377230 | 8.3144187 | 0.9974987 |
| Ni | 0.3432157 | 0.2370813 | -0.890283 |

**Br-$Ni^{III}$-mpg-$CN_x$** (doublet, G = -6576.954933)

| | | | |
|---|---|---|---|
| H | 8.5588949 | 1.3248023 | 2.3557194 |
| N | 8.1429254 | 0.4248237 | 2.1533368 |
| C | 6.9552223 | 0.3546802 | 1.5545803 |
| N | 6.3545996 | 1.5348430 | 1.2564498 |
| C | 5.1937284 | 1.4721066 | 0.6342488 |
| N | 4.6528427 | 0.2163375 | 0.3045435 |
| C | 5.3051212 | -0.965715 | 0.6988787 |

| | | | |
|---|---|---|---|
| N | 6.4653881 | -0.889410 | 1.3206939 |
| N | 2.8766287 | 1.2568896 | -0.832923 |
| C | 3.3519337 | 2.3975341 | -0.330251 |
| N | 4.4918865 | 2.5792629 | 0.3399311 |
| C | 3.4881861 | 0.1429890 | -0.454928 |
| N | 2.9816192 | -1.040437 | -0.772041 |
| C | 3.5577015 | -2.104837 | -0.210952 |
| N | 4.7078292 | -2.146238 | 0.4651826 |
| N | 2.8969728 | -3.319447 | -0.372356 |
| C | 1.5624226 | -3.466563 | -0.669153 |
| N | 1.1874657 | -4.612821 | -1.238695 |
| C | -0.087321 | -4.702246 | -1.647005 |
| N | -0.917003 | -3.568608 | -1.533614 |
| N | 0.7546237 | -2.437400 | -0.340309 |
| C | -0.456226 | -2.478380 | -0.832639 |
| C | -2.216052 | -3.572096 | -2.072095 |
| N | -0.576435 | -5.799619 | -2.185871 |
| C | -1.863780 | -5.756914 | -2.611739 |
| N | -2.373258 | -6.881405 | -3.101287 |
| N | -2.690329 | -4.672186 | -2.603005 |
| N | -2.917359 | -2.422551 | -2.060985 |
| C | -2.393501 | -1.389308 | -1.419240 |
| N | -1.265821 | -1.410911 | -0.678653 |
| N | -3.059441 | -0.185448 | -1.555150 |
| H | -3.909378 | -0.239056 | -2.113450 |
| N | -1.386966 | 1.2429531 | -0.749088 |
| C | -2.506281 | 1.0795784 | -1.484658 |
| N | -3.120644 | 2.0254188 | -2.178783 |
| C | -2.527016 | 3.2319048 | -2.252358 |
| N | -3.098348 | 4.2545797 | -2.839484 |
| C | -2.374124 | 5.4081665 | -2.907520 |
| N | -1.097033 | 5.5904784 | -2.487706 |
| N | -2.982832 | 6.4540934 | -3.454528 |
| N | -1.234344 | 3.3753116 | -1.717636 |
| C | -0.677770 | 2.3702036 | -0.961136 |
| C | -0.511198 | 4.5722740 | -1.892818 |
| N | 0.7654690 | 4.6211410 | -1.483488 |
| C | 1.2419546 | 3.5455992 | -0.854972 |
| N | 0.5305522 | 2.4659261 | -0.470198 |
| N | 2.5836391 | 3.5366359 | -0.553980 |
| H | 8.6387000 | -0.421539 | 2.4019906 |
| H | 3.4576811 | -4.165724 | -0.323207 |
| H | -1.808022 | -7.720281 | -3.147385 |
| H | -3.324428 | -6.902043 | -3.448001 |
| H | 3.0647848 | 4.4318912 | -0.551059 |
| H | -3.931109 | 6.3696101 | -3.799485 |
| H | -2.496325 | 7.3374010 | -3.546226 |
| Ni | -0.600625 | -0.016701 | 0.5755413 |
| C | -3.693422 | -1.314698 | 3.0164311 |
| C | -4.338004 | -0.114069 | 3.3566783 |
| C | -2.519118 | -1.282183 | 2.2649679 |
| C | -5.536779 | -0.148284 | 4.1382001 |
| H | -2.008870 | -2.209790 | 2.0306660 |
| C | -3.805136 | 1.1207552 | 2.9517006 |
| C | -2.036012 | -0.048806 | 1.8393432 |
| H | -4.295860 | 2.0471261 | 3.2326483 |
| C | -2.630953 | 1.1560139 | 2.2001641 |
| H | -2.207122 | 2.1126754 | 1.9157767 |
| H | -4.098022 | -2.265873 | 3.3472947 |
| Br | 0.7155420 | 0.0923196 | 2.4182416 |
| N | -6.510631 | -0.176032 | 4.7744494 |

**$N_3$-$Ni^{III}$-mpg-$CN_x$** (doublet, G = -4166.940708)

| | | | |
|---|---|---|---|
| H | 8.7659107 | -0.606159 | 1.8501423 |
| N | 8.1507000 | -1.381371 | 1.6388875 |
| C | 6.9256883 | -1.156747 | 1.1670520 |
| N | 6.5684676 | 0.1416008 | 0.9964610 |
| C | 5.3669058 | 0.3686933 | 0.5028013 |
| N | 4.5427926 | -0.718638 | 0.1617394 |
| C | 4.9607633 | -2.037145 | 0.4158282 |
| N | 6.1606793 | -2.250481 | 0.9189839 |
| N | 2.9337996 | 0.7523278 | -0.719448 |
| C | 3.6787398 | 1.7264545 | -0.193118 |
| N | 4.8894568 | 1.6159474 | 0.3575726 |
| C | 3.3270533 | -0.489737 | -0.474976 |
| N | 2.5522230 | -1.513982 | -0.805291 |
| C | 2.9417681 | -2.718096 | -0.383259 |
| N | 4.1109425 | -3.048352 | 0.1692085 |
| N | 2.0337174 | -3.756741 | -0.568075 |
| C | 0.6835564 | -3.603840 | -0.783681 |
| N | 0.0607895 | -4.573218 | -1.452954 |
| C | -1.222728 | -4.361831 | -1.782650 |
| N | -1.800170 | -3.109993 | -1.489168 |
| N | 0.1129379 | -2.486182 | -0.282870 |
| C | -1.100376 | -2.226305 | -0.702132 |
| C | -3.091364 | -2.789198 | -1.948473 |
| N | -1.944352 | -5.266932 | -2.408859 |
| C | -3.209981 | -4.918326 | -2.751539 |
| N | -3.953348 | -5.851533 | -3.333975 |
| N | -3.794812 | -3.699004 | -2.576136 |
| N | -3.546176 | -1.534818 | -1.764334 |
| C | -2.802938 | -0.712342 | -1.040805 |
| N | -1.673643 | -1.047468 | -0.383113 |
| N | -3.214821 | 0.6042131 | -0.980085 |
| H | -4.088261 | 0.7929317 | -1.467820 |
| N | -1.226263 | 1.5684191 | -0.185711 |
| C | -2.406985 | 1.7185761 | -0.822646 |
| N | -2.871336 | 2.8419198 | -1.348428 |
| C | -2.054502 | 3.9114408 | -1.352371 |
| N | -2.455827 | 5.0901156 | -1.761917 |
| C | -1.523528 | 6.0844027 | -1.780941 |
| N | -0.203308 | 5.9609995 | -1.497438 |
| N | -1.954941 | 7.2907770 | -2.131318 |
| N | -0.720377 | 3.7338293 | -0.946883 |
| C | -0.316749 | 2.5518722 | -0.360587 |
| C | 0.2128904 | 4.7819569 | -1.083539 |
| N | 1.5063974 | 4.5327394 | -0.830437 |
| C | 1.8071019 | 3.3157306 | -0.375759 |
| N | 0.9295355 | 2.3591389 | -0.012155 |
| N | 3.1424017 | 3.0093155 | -0.244313 |
| H | 8.4740910 | -2.328930 | 1.7857569 |
| H | 2.4128156 | -4.696157 | -0.650674 |
| H | -3.574632 | -6.776577 | -3.496094 |
| H | -4.904408 | -5.645870 | -3.614218 |
| H | 3.7963207 | 3.7868674 | -0.218018 |
| H | -2.928809 | 7.4352258 | -2.367754 |
| H | -1.309795 | 8.0699033 | -2.176863 |
| Ni | -0.709144 | -0.013069 | 0.9749749 |
| C | -3.620297 | -1.275123 | 3.6419366 |
| C | -4.141284 | -0.064137 | 4.1269276 |
| C | -2.576734 | -1.258328 | 2.7166305 |
| C | -5.209860 | -0.081100 | 5.0797005 |
| H | -2.168900 | -2.196854 | 2.3558464 |
| C | -3.615088 | 1.1630501 | 3.6932238 |
| C | -2.083140 | -0.030071 | 2.2841911 |
| H | -4.014938 | 2.0958109 | 4.0781974 |
| C | -2.568669 | 1.1807464 | 2.7701577 |
| H | -2.154778 | 2.1325507 | 2.4547958 |
| H | -4.024372 | -2.221279 | 3.9876875 |
| N | -6.078486 | -0.094293 | 5.8537538 |
| N | 0.5369040 | 0.0923332 | 2.3103629 |

| | | | |
|---|---|---|---|
| N | 0.5166329 | 0.4751756 | 3.4614243 |
| N | 0.5765385 | 0.8278335 | 4.5541937 |

**Ni^I-NArN$_2$-mpg-CN$_x$** (doublet, G = -4166.970787)

| | | | |
|---|---|---|---|
| H | 8.6311758 | 0.1615414 | 1.2624849 |
| N | 8.0810796 | -0.660662 | 1.0495991 |
| C | 6.8245823 | -0.535764 | 0.6244188 |
| N | 6.3432082 | 0.7282452 | 0.5153029 |
| C | 5.1116391 | 0.8599169 | 0.0611298 |
| N | 4.3803217 | -0.287739 | -0.294271 |
| C | 4.9231960 | -1.569847 | -0.093351 |
| N | 6.1547589 | -1.686968 | 0.3642702 |
| N | 2.6132727 | 1.0555094 | -1.072591 |
| C | 3.2888969 | 2.0805916 | -0.548306 |
| N | 4.5227101 | 2.0627041 | -0.036346 |
| C | 3.1277395 | -0.152846 | -0.886209 |
| N | 2.4408279 | -1.233823 | -1.228673 |
| C | 2.9438987 | -2.409219 | -0.843630 |
| N | 4.1568351 | -2.645645 | -0.335738 |
| N | 2.1213362 | -3.516544 | -1.012219 |
| C | 0.7499577 | -3.478476 | -1.157930 |
| N | 0.1839649 | -4.497487 | -1.808121 |
| C | -1.129252 | -4.399238 | -2.068861 |
| N | -1.797911 | -3.206483 | -1.731726 |
| N | 0.1165557 | -2.419701 | -0.620529 |
| C | -1.140208 | -2.262404 | -0.978817 |
| C | -3.130109 | -2.991096 | -2.129556 |
| N | -1.800575 | -5.361836 | -2.666129 |
| C | -3.109382 | -5.121549 | -2.938720 |
| N | -3.798516 | -6.119617 | -3.484989 |
| N | -3.786655 | -3.961319 | -2.727707 |
| N | -3.674505 | -1.781122 | -1.920462 |
| C | -2.957475 | -0.895799 | -1.233569 |
| N | -1.778744 | -1.133754 | -0.628238 |
| N | -3.476100 | 0.3883255 | -1.170507 |
| H | -4.373421 | 0.4948594 | -1.638227 |
| N | -1.573783 | 1.5324093 | -0.418087 |
| C | -2.773725 | 1.5745975 | -1.026945 |
| N | -3.350558 | 2.6593868 | -1.536422 |
| C | -2.626722 | 3.7900411 | -1.565494 |
| N | -3.129103 | 4.9346343 | -1.976068 |
| C | -2.280141 | 5.9956069 | -2.023126 |
| N | -0.948243 | 5.9867577 | -1.754889 |
| N | -2.806948 | 7.1616035 | -2.387427 |
| N | -1.274293 | 3.7313855 | -1.183376 |
| C | -0.769122 | 2.5920481 | -0.603566 |
| C | -0.429823 | 4.8480981 | -1.343655 |
| N | 0.8837924 | 4.6991633 | -1.113144 |
| C | 1.2874617 | 3.5121541 | -0.652640 |
| N | 0.4987299 | 2.4936145 | -0.263084 |
| N | 2.6481934 | 3.3139829 | -0.556734 |
| H | 8.4955837 | -1.578226 | 1.1500402 |
| H | 2.5734851 | -4.421354 | -1.107902 |
| H | -3.346789 | -7.004406 | -3.678813 |
| H | -4.774190 | -5.994564 | -3.723839 |
| H | 3.2365599 | 4.1421501 | -0.531379 |
| H | -3.791334 | 7.2283196 | -2.613696 |
| H | -2.223657 | 7.9862684 | -2.453252 |
| Ni | -0.741751 | 0.0513580 | 0.4972764 |
| C | -1.585054 | -0.523465 | 5.7945695 |
| C | -2.952085 | -0.386186 | 5.4965194 |
| C | -0.640928 | -0.471282 | 4.7767630 |
| C | -3.921443 | -0.441156 | 6.5467221 |
| H | 0.4118376 | -0.578483 | 5.0189417 |
| C | -3.366886 | -0.194537 | 4.1668177 |
| C | -1.065401 | -0.280045 | 3.4565170 |
| H | -4.421676 | -0.088415 | 3.9358864 |
| C | -2.423812 | -0.141647 | 3.1507268 |
| H | -2.722503 | 0.0064111 | 2.1186110 |
| H | -1.264875 | -0.670733 | 6.8206708 |
| N | -4.710816 | -0.484865 | 7.4010506 |
| N | -0.162271 | -0.212438 | 2.3475537 |
| N | 1.0606609 | -0.341707 | 2.6120435 |
| N | 2.1888926 | -0.441551 | 2.7191061 |

**Ni^III-NHAr-mpg-CN$_x$** (doublet, G = -4057.904463)

| | | | |
|---|---|---|---|
| H | 8.300874 | 0.9727080 | -1.649063 |
| N | 7.799037 | 0.0938000 | -1.664998 |
| C | 6.468199 | 0.0875740 | -1.641846 |
| N | 5.851418 | 1.2959610 | -1.585676 |
| C | 4.533607 | 1.2937970 | -1.599513 |
| N | 3.845385 | 0.0694510 | -1.679703 |
| C | 4.551508 | -1.146878 | -1.670596 |
| N | 5.869358 | -1.130790 | -1.661848 |
| N | 1.793603 | 1.2108030 | -1.824264 |
| C | 2.499208 | 2.3135780 | -1.571589 |
| N | 3.825998 | 2.4326190 | -1.500007 |
| C | 2.46041 | 0.0630630 | -1.809593 |
| N | 1.810044 | -1.088178 | -1.891060 |
| C | 2.531726 | -2.195223 | -1.696254 |
| N | 3.860255 | -2.298986 | -1.637880 |
| N | 1.814512 | -3.381021 | -1.564403 |
| C | 0.4799020 | -3.475178 | -1.222584 |
| N | -0.170029 | -4.575741 | -1.604868 |
| C | -1.493600 | -4.604861 | -1.387433 |
| N | -2.123349 | -3.461783 | -0.854413 |
| N | -0.038433 | -2.447253 | -0.518224 |
| C | -1.341673 | -2.428718 | -0.403985 |
| C | -3.524610 | -3.401701 | -0.734124 |
| N | -2.229306 | -5.651789 | -1.698663 |
| C | -3.566332 | -5.549514 | -1.492908 |
| N | -4.299659 | -6.620851 | -1.774789 |
| N | -4.242060 | -4.451625 | -1.049387 |
| N | -4.088044 | -2.240817 | -0.344530 |
| C | -3.280143 | -1.254166 | 0.0124180 |
| N | -1.940833 | -1.349649 | 0.1359770 |
| N | -3.875715 | -0.024575 | 0.2378489 |
| H | -4.888439 | -0.029330 | 0.1322819 |
| N | -1.960013 | 1.3291491 | 0.2184799 |
| C | -3.299046 | 1.2234601 | 0.0853609 |
| N | -4.113516 | 2.2170940 | -0.218842 |
| C | -3.564920 | 3.4063620 | -0.533233 |
| N | -4.293902 | 4.4669180 | -0.774862 |
| C | -3.626618 | 5.5994140 | -1.151671 |
| N | -2.292095 | 5.7256480 | -1.360886 |
| N | -4.369978 | 6.6842420 | -1.346517 |
| N | -2.164229 | 3.4914460 | -0.653300 |
| C | -1.369524 | 2.4421641 | -0.261926 |
| C | -1.544508 | 4.6695480 | -1.119559 |
| N | -0.221581 | 4.6536640 | -1.346609 |
| C | 0.4383249 | 3.5423450 | -1.031645 |
| N | -0.066359 | 2.4703961 | -0.381371 |
| N | 1.764606 | 3.4817480 | -1.384496 |
| H | 8.315821 | -0.775617 | -1.705800 |
| H | 2.2847330 | -4.241648 | -1.814281 |
| H | -3.861711 | -7.465490 | -2.120838 |
| H | -5.305112 | -6.597712 | -1.644861 |
| H | 2.231848 | 4.3620740 | -1.581928 |

| | | | |
|---|---|---|---|
| H | -5.372427 | 6.6449760 | -1.205832 |
| H | -3.940138 | 7.556659 | -1.624524 |
| Ni | -0.709138 | -0.024619 | 0.89077 |
| C | 2.348127 | -0.026524 | 5.3549709 |
| C | 1.1691820 | -0.209144 | 6.1300009 |
| C | 2.251547 | 0.0477027 | 3.988321 |
| C | 1.2674300 | -0.288390 | 7.5525439 |
| H | 3.1458669 | 0.1898599 | 3.3744590 |
| C | -0.108431 | -0.327168 | 5.5097999 |
| C | 0.9746459 | -0.061726 | 3.340293 |
| H | -0.995262 | -0.468649 | 6.1247159 |
| C | -0.206351 | -0.254771 | 4.1443429 |
| H | -1.177404 | -0.346136 | 3.6572089 |
| H | 3.311082 | 0.0618263 | 5.8527809 |
| N | 1.3481780 | -0.354676 | 8.7117649 |
| N | 0.8654749 | 0.0196401 | 2.007164 |
| H | 1.7685479 | 0.1431792 | 1.5468220 |

**H-Ni$^{III}$-NHAr-mpg-CN$_x$** (doublet, G = -4058.648024)

| | | | |
|---|---|---|---|
| H | 8.9833071 | 0.8893389 | 2.6828863 |
| N | 8.5087129 | 0.0160881 | 2.4927065 |
| C | 7.3280389 | 0.0159097 | 1.8759944 |
| N | 6.8209801 | 1.2272302 | 1.5344985 |
| C | 5.6659604 | 1.2316588 | 0.8979436 |
| N | 5.0448043 | 0.0090540 | 0.5839665 |
| C | 5.6034077 | -1.205537 | 1.0207280 |
| N | 6.7510487 | -1.195278 | 1.6696085 |
| N | 3.3641613 | 1.1457249 | -0.604985 |
| C | 3.9025828 | 2.2607835 | -0.106561 |
| N | 5.0419190 | 2.3772436 | 0.5792705 |
| C | 3.8930316 | 0.0000136 | -0.197435 |
| N | 3.3110497 | -1.153106 | -0.500393 |
| C | 3.8088906 | -2.245136 | 0.0835967 |
| N | 4.9346377 | -2.348242 | 0.7929702 |
| N | 3.0857798 | -3.421799 | -0.091235 |
| C | 1.7557141 | -3.497114 | -0.435150 |
| N | 1.3458455 | -4.608249 | -1.047168 |
| C | 0.0861854 | -4.619173 | -1.509459 |
| N | -0.681048 | -3.440595 | -1.414934 |
| N | 0.9854218 | -2.440015 | -0.103339 |
| C | -0.200963 | -2.396340 | -0.658552 |
| C | -1.948900 | -3.358302 | -2.020286 |
| N | -0.438981 | -5.678642 | -2.087797 |
| C | -1.696999 | -5.552353 | -2.580608 |
| N | -2.244121 | -6.639028 | -3.113536 |
| N | -2.453548 | -4.419028 | -2.603199 |
| N | -2.585062 | -2.172393 | -2.015919 |
| C | -2.041504 | -1.187127 | -1.314972 |
| N | -0.960117 | -1.291241 | -0.516835 |
| N | -2.627741 | 0.0568181 | -1.449429 |
| H | -3.453821 | 0.0645913 | -2.043724 |
| N | -0.894567 | 1.3798754 | -0.576155 |
| C | -1.999305 | 1.2872542 | -1.344868 |
| N | -2.545443 | 2.2676953 | -2.051636 |
| C | -1.886481 | 3.4385201 | -2.101919 |
| N | -2.391730 | 4.4961951 | -2.691121 |
| C | -1.609472 | 5.6109995 | -2.729372 |
| N | -0.330292 | 5.7173978 | -2.291354 |
| N | -2.153309 | 6.6965745 | -3.268734 |
| N | -0.599802 | 3.5052388 | -1.539847 |
| C | -0.117710 | 2.4644661 | -0.771020 |
| C | 0.1891232 | 4.6625174 | -1.698047 |
| N | 1.4633883 | 4.6391224 | -1.278967 |
| C | 1.8681440 | 3.5351997 | -0.650480 |
| N | 1.0907608 | 2.5043207 | -0.266289 |
| N | 3.2078303 | 3.4422205 | -0.345639 |
| H | 8.9357563 | -0.856801 | 2.7750517 |
| H | 3.6018230 | -4.295402 | -0.035366 |
| H | -1.731390 | -7.511984 | -3.129683 |
| H | -3.180427 | -6.604014 | -3.497594 |
| H | 3.7414879 | 4.3067537 | -0.330918 |
| H | -3.101113 | 6.6688261 | -3.623667 |
| H | -1.619466 | 7.5541103 | -3.337950 |
| Ni | -0.312490 | -0.009488 | 0.8295545 |
| C | -2.874888 | -1.505402 | 3.7234984 |
| C | -3.871724 | -0.519652 | 3.8241725 |
| C | -1.796180 | -1.308751 | 2.8631018 |
| C | -4.980220 | -0.712531 | 4.7082651 |
| H | -1.023210 | -2.070633 | 2.8059002 |
| C | -3.779993 | 0.6596046 | 3.0658339 |
| C | -1.718694 | -0.144119 | 2.0915571 |
| H | -4.545638 | 1.4242117 | 3.1550208 |
| C | -2.697537 | 0.8463997 | 2.2074978 |
| H | -2.631716 | 1.7696322 | 1.6406592 |
| H | -2.944965 | -2.411727 | 4.3172230 |
| N | -5.883652 | -0.866868 | 5.4259879 |
| N | 0.7123605 | 0.6648968 | 2.1640076 |
| H | 0.8816735 | 1.6672857 | 2.0505150 |
| H | 0.3990658 | 0.5408793 | 3.1279692 |

**CN-Ar-Br** (singlet, G = -2898.200356)

| | | | |
|---|---|---|---|
| C | -1.061146 | 1.2853574 | 0.0001394 |
| C | -1.794330 | 0.0866689 | -0.000024 |
| C | 0.3298099 | 1.2462007 | 0.0001652 |
| C | -3.225064 | 0.1272413 | -0.000047 |
| H | 0.8995364 | 2.1688660 | 0.0002934 |
| C | -1.130332 | -1.151678 | -0.000166 |
| C | 0.9730173 | 0.0080865 | 0.0000212 |
| H | -1.700051 | -2.075173 | -0.000291 |
| C | 0.2606158 | -1.191526 | -0.000146 |
| H | 0.7770256 | -2.145056 | -0.000258 |
| H | -1.577524 | 2.2397019 | 0.0002454 |
| Br | 2.8840528 | -0.046111 | 0.0000529 |
| N | -4.388287 | 0.1602606 | -0.000057 |

**$NaN_3$** (singlet, G = -326.6482243)

| | | | |
|---|---|---|---|
| Na | 0.1133698 | 0.0121609 | 2.1441277 |
| N | -0.322942 | -0.035261 | 0.0242128 |
| N | -0.054080 | -0.005523 | -1.136664 |
| N | 0.1915123 | 0.0206082 | -2.282045 |

**NaBr** (singlet, G = -2736.672448)

| | | | |
|---|---|---|---|
| Na | 0. | 0. | 0.9901365 |
| Br | 0. | 0. | -1.579772 |

**$N_2$** (singlet, G = -109.5768959)

| | | | |
|---|---|---|---|
| N | 0. | 0. | -3.867314 |
| N | 0. | 0. | -4.972321 |

**Ar-$NH_2$** (singlet, G = -379.9349338)

| | | | | | | | |
|---|---|---|---|---|---|---|---|
| C | -0.661289 | -1.162615 | 0.1389606 | H | 1.0028479 | 2.3444309 | -0.012647 |
| C | -1.464518 | -0.010623 | 0.0110418 | H | -1.128445 | -2.142225 | 0.1770892 |
| C | 0.7165527 | -1.052184 | 0.2112744 | N | -4.042690 | -0.217322 | -0.129208 |
| C | -2.881675 | -0.124356 | -0.066184 | N | 2.7062918 | 0.3275090 | 0.2863280 |
| H | 1.3293728 | -1.944564 | 0.3086413 | H | 3.1298990 | 1.1887560 | -0.038909 |
| C | -0.845337 | 1.2552304 | -0.042468 | H | 3.2583921 | -0.498487 | 0.0866558 |
| C | 1.3432219 | 0.2143126 | 0.1599380 | | | | |
| H | -1.455012 | 2.1480686 | -0.144696 | | | | |
| C | 0.5325095 | 1.3654866 | 0.0301406 | | | | |

## 8. Supporting References.


(1) Roy, S.; Reisner, E. Visible-Light-Driven CO2 Reduction by Mesoporous Carbon Nitride Modified with Polymeric Cobalt Phthalocyanine. *Angew. Chemie Int. Ed.* **2019**, *58*, 12180–12184.

(2) Kasap, H.; Caputo, C. A.; Martindale, B. C. M.; Godin, R.; Lau, V. W. H.; Lotsch, B. V.; Durrant, J. R.; Reisner, E. Solar-Driven Reduction of Aqueous Protons Coupled to Selective Alcohol Oxidation with a Carbon Nitride-Molecular Ni Catalyst System. *J. Am. Chem. Soc.* **2016**, *138*, 9183–9192.

(3) Liu, J.; Liu, Y.; Liu, N.; Han, Y.; Zhang, X.; Huang, H.; Lifshitz, Y.; Lee, S.-T.; Zhong, J.; Kang, Z. Metal-Free Efficient Photocatalyst for Stable Visible Water Splitting via a Two-Electron Pathway. *Science.* **2015**, *347*.

(4) Lau, V. W.; Moudrakovski, I.; Botari, T.; Weinberger, S.; Mesch, M. B.; Duppel, V.; Senker, J.; Blum, V.; Lotsch, B. V. Rational Design of Carbon Nitride Photocatalysts by Identification of Cyanamide Defects as Catalytically Relevant Sites. *Nat. Commun.* **2016**, *7*, 12165.

(5) Vijeta, A.; Casadevall, C.; Roy, S.; Reisner, E. Visible-Light Promoted C–O Bond Formation with an Integrated Carbon Nitride–Nickel Heterogeneous Photocatalyst. *Angew. Chemie Int. Ed.* **2021**, *60*, 8494–8499.

(6) Call, A.; Casadevall, C.; Acuña-Parés, F.; Casitas, A.; Lloret-Fillol, J. Dual Cobalt–Copper Light-Driven Catalytic Reduction of Aldehydes and Aromatic Ketones in Aqueous Media. *Chem. Sci.* **2017**, *8*, 4739–4749.

(7) Chandna, N.; Kaur, F.; Kumar, S.; Jain, N. Glucose Promoted Facile Reduction of Azides to Amines under Aqueous Alkaline Conditions. *Green Chem.* **2017**, *19*, 4268–4271.

(8) Gaussian 16, Revision C.01, M. J. Frisch, G. W. Trucks, H. B. Schlegel, G. E. Scuseria, M. A. Robb, J. R. Cheeseman, G. Scalmani, V. Barone, G. A. Petersson, H. Nakatsuji, X. Li, M. Caricato, A. V. Marenich, J. Bloino, B. G. Janesko, R. Gomperts, B. Mennucci, H. P. Hratchian, J. V. Ortiz, A. F. Izmaylov, J. L. Sonnenberg, D. Williams-Young, F. Ding, F. Lipparini, F. Egidi, J. Goings, B. Peng, A. Petrone, T. Henderson, D. Ranasinghe, V. G. Zakrzewski, J. Gao, N. Rega, G. Zheng, W. Liang, M. Hada, M. Ehara, K. Toyota, R. Fukuda, J. Hasegawa, M. Ishida, T. Nakajima, Y. Honda, O. Kitao, H. Nakai, T. Vreven, K. Throssell, J. A. Montgomery, Jr., J. E. Peralta, F. Ogliaro, M. J. Bearpark, J. J. Heyd, E. N. Brothers, K. N. Kudin, V. N. Staroverov, T. A. Keith, R. Kobayashi, J. Normand, K. Raghavachari, A. P. Rendell, J. C. Burant, S. S. Iyengar, J.

Tomasi, M. Cossi, J. M. Millam, M. Klene, C. Adamo, R. Cammi, J. W. Ochterski, R. L. Martin, K. Morokuma, O. Farkas, J. B. Foresman, and D. J. Fox, Gaussian, Inc., Wallingford CT, **2016**.

(9) Becke, A. D. Density-functional Thermochemistry. III. The Role of Exact Exchange. *J. Chem. Phys.* **1993**, *98*, 5648–5652.

(10) Lee, C.; Yang, W.; Parr, R. G. Development of the Colle-Salvetti Correlation-Energy Formula into a Functional of the Electron Density. *Phys. Rev. B* **1988**, *37*, 785–789.

(11) Dolg, M.; Wedig, U.; Stoll, H.; Preuss, H. Energy-adjusted a b i n i t i o Pseudopotentials for the First Row Transition Elements. *J. Chem. Phys.* **1987**, *86*, 866–872.

(12) Hariharan, P. C.; Pople, J. A. The Influence of Polarization Functions on Molecular Orbital Hydrogenation Energies. *Theor. Chim. Acta* **1973**, *28*, 213–222.

(13) Hehre, W. J.; Ditchfield, R.; Pople, J. A. Self–Consistent Molecular Orbital Methods. XII. Further Extensions of Gaussian–Type Basis Sets for Use in Molecular Orbital Studies of Organic Molecules. *J. Chem. Phys.* **1972**, *56*, 2257–2261.

(14) Marenich, A. V.; Cramer, C. J.; Truhlar, D. G. Universal Solvation Model Based on Solute Electron Density and on a Continuum Model of the Solvent Defined by the Bulk Dielectric Constant and Atomic Surface Tensions. *J. Phys. Chem. B* **2009**, *113*, 6378–6396.

(15) Svensson, M.; Humbel, S.; Froese, R. D. J.; Matsubara, T.; Sieber, S.; Morokuma, K. ONIOM: A Multilayered Integrated MO + MM Method for Geometry Optimizations and Single Point Energy Predictions. A Test for Diels−Alder Reactions and Pt(P( t -Bu) 3 ) 2 + H 2 Oxidative Addition. *J. Phys. Chem.* **1996**, *100*, 19357–19363.

(16) Dunning, T. H. Gaussian Basis Sets for Use in Correlated Molecular Calculations. I. The Atoms Boron through Neon and Hydrogen. *J. Chem. Phys.* **1989**, *90*, 1007–1023.

(17) Hashimoto, T.; Hirao, K.; Tatewaki, H. Comment on Dunning's Correlation-Consistent Basis Sets. *Chem. Phys. Lett.* **1995**, *243*, 190–192.

(18) Winget, P.; Cramer, C. J.; Truhlar, D. G. Computation of Equilibrium Oxidation and Reduction Potentials for Reversible and Dissociative Electron-Transfer Reactions in Solution. *Theor. Chem. Acc.* **2004**, *112*, 217–227.

(19) Vo, G. D.; Hartwig, J. F. Palladium-Catalyzed Coupling of Ammonia with Aryl Chlorides, Bromides, Iodides, and Sulfonates: A General Method for the Preparation of Primary Arylamines. *J. Am. Chem. Soc.* **2009**, *131*, 11049–11061.

(20) Han, D.; Li, S.; Xia, S.; Su, M.; Jin, J. Nickel-Catalyzed Amination of (Hetero)Aryl Halides Facilitated by a Catalytic Pyridinium Additive. *Chem. A Eur. J.* **2020**, *26*, 12349–12354.

End of Supporting Information
